# Age-related processes impacting cognitive processing speed across an older adult cohort: A drift diffusion model analysis

**DOI:** 10.1371/journal.pone.0361899

**Published:** 2026-11-13

**Authors:** Clara Sanches, Noah Cryns, J. Clayton Young, Heather Romero-Kornblum, Chenyu Wang, Yann Cobigo, Maria Luisa Mandelli, Winston Chiong

**Affiliations:** 1 Memory and Aging Center, Department of Neurology, University of California, San Francisco, California, United States of America; 2 Rady School of Management, University of California, San Diego, United States of America; Chhatrapati Shahu Ji Maharaj University, INDIA

## Abstract

The time we take to perform a cognitive task increases with age, which is a principal contributor to cognitive aging. The drift diffusion model is widely applied to decompose processes that may contribute to age-related slowing, with the *drift rate* parameter reflecting the rate of information accumulation until a decision is made. This age-related slowing is hypothesized to relate to changes in cerebral white matter microstructure. Our aim in this preregistered study (https://osf.io/yhn4m) was to evaluate the cross-sectional age-related differences in processes underlying age-related slowing across an older adult cohort and explore the role of white matter change in such age-related slowing. We found age to be associated with *drift rate* in an *emotion recognition* and a *memory* task but not in a *delay discounting* task; in an exploratory analysis, an indirect association involving global fractional anisotropy was observed for the *emotion recognition* task, although this did not survive multiple-comparison correction. Our results suggest age-related differences in information processing across cognitive contexts, although formal comparisons between tasks were not conducted. A fuller understanding of the mechanisms of cognitive slowing with age will help tailor intervention strategies and could inform environmental modifications promoting older adults’ functional independence and quality of life.

## Introduction

Age-related changes in cognitive capacities impact individuals’ functional independence in everyday tasks such as managing finances, grocery shopping, driving and social engagement. The time it takes an individual to perform a cognitive task is used as a measure of the individual’s cognitive processing speed, but it includes different components such as identifying the stimulus, manipulating information and deciding how to respond and, finally, responding to the information received [1]. Age-related slowing is observed across a similarly wide and heterogenous range of cognitive operations. Reaction time (RT) has been shown to increase with age, even in healthy adults [2]; and the general slowing hypothesis, which proposes that cognitive aging can be regarded as a general slowing of information processing due to increased neural noise [3], is widely accepted in the literature [4,5].

It has been suggested that measures based solely on RTs may lack specificity as measures of cognitive processing speed, as RT is also influenced by changes in non-cognitive processes [1,6]. The drift diffusion model (DDM) represents one widely applied approach to the temporal decomposition of contributing processes in decision-making. The DDM takes both response choices and RT distributions into account and allows for the statistical separation of different components of binary decisions that reflect psychologically meaningful characteristics of the decision process [7] as: those associated with the speed of information accumulation and decision-making (*drift rate*), those associated with decision thresholds to reach a response (representing speed-accuracy tradeoff; *boundary separation*), and those associated with sensory encoding and motor execution (*non-decision time*) [8]. In this model, the *drift rate* may be considered a purer measure of cognitive processing speed than RT [1,6]. For a measure to be considered pure from the point of view of representing a specific cognitive process it must show both convergent and divergent validity [9]. The convergent validity of this model has been consistently demonstrated by studies using recognition memory tasks [9] but also on slow-RT paradigms [10]. These studies performed experimental manipulations to target only one of the three main parameters of the DDM at a time and showed that the manipulations affected mainly the intended parameter (convergent validity), even though the manipulations also had a slight influence on other parameters, showing a less robust divergent validity [9,10]. Moreover, test-retest reliability was shown to be satisfactory for *drift rate* and *boundary separation* and, even though at a lower degree, for *non-decision time,* on recognition memory and lexical decision tasks and on an associative priming paradigm [11]. Of note, this study also showed that, while reliability of parameter estimation increases with increasing trial number, increasing trial numbers from 100–200 trials has a low effect on reliability [11], suggesting that parameters of the DDM can be reliably estimated even with a relatively low number of trials.

Whereas *boundary separation* and *non-decision time* have been consistently shown to increase with age, the literature shows inconsistent results regarding age effects on *drift rate* [12]. For example, while significant differences in *drift rate* between younger and older participants have been found for letter and brightness discrimination tasks [13], for complex rectangle area judgement and statement generation tasks [5] and for episodic retrieval tasks [14], no differences were found between such two groups in signal detection [13], recognition memory [13], lexical decision [4,13] or semantic retrieval tasks [14]. These findings from diffusion model analyses have challenged the view that information processing generally declines with age [12].

Cognitive efficiency relies on information transfer between brain regions [1], which itself depends on the organization of white-matter pathways [15]. Congruently, Turken and colleagues (2008) [16] and Kuznetsova and colleagues (2016) [17] have demonstrated that cognitive processing speed is associated with the structural integrity of white-matter tracts. Other findings indicate that white matter changes contribute to age-related declines in cognitive processes, including processing speed [2,18]. Very few studies have investigated the association between white matter microstructure measures and the different parameters of the DDM. However, Madden and colleagues (2009) [19] applied the DDM to a task switching paradigm, comparing the DDM parameter estimates in young versus older adults and found that the association between age and *drift rate* showed a significant indirect effect through white matter measures within the frontoparietal network, but the association between age and the *non-decision time* parameter did not show such effects. Conversely, Yang and colleagues (2015) [20] explored the association between white matter microstructure measures and DDM parameters in a perceptual task across the lifespan and reported that white-matter changes with age contributed to the associations between age and the *non-decision time* parameter only.

A fuller understanding of the neurobiological mechanisms for cognitive slowing in aging will contribute to our understanding of age-related cognitive decline, will help tailor intervention strategies to specific mechanisms, and could inform environmental modifications to promote older adults’ functional independence and quality of life [21]. The DDM has proved useful in distinguishing between different processes involved in task performance and thus may facilitate further insight into how processing speed and its specific components change with age.

Most studies cited above using the DDM have compared groups of young adults to older adults. Because younger cohorts are often recruited through different mechanisms than older cohorts (e.g., recruiting younger adults from the same undergraduate college and recruiting older adults from community organizations), comparison across two samples can introduce uncontrolled differences. Comparison across two separate cohorts also does not permit observation of the cross-sectional age-related differences of *drift rate*, *boundary decision* and *non-decision times* with age. Of the studies that have analyzed age as a continuous variable, most included participants only until 80 years old, limiting the study of such effects among the oldest old, a group predicted to be particularly impacted. One study focused on differences across young and mid-adulthood (ages 18–62 years) and reported linear correlations of age with both *boundary separation* and *non-decision times* but no correlations with *drift rates* [5], across a large range of tasks and domains, including figural, numerical and verbal tasks. However, a second study from the same group explored this question on a larger sample spanning from 10–80 years old; while reporting similar results as the previous ones for *boundary separation* and *non-decision times*, they also reported that *drift rates* only begin showing a linear decrease with age from 60 years onwards [22] but this time applying a unique race implicit association task. Yang and colleagues (2015) [20] reported decreases in *drift rate* and increases in both *boundary separation* and *non-decision time* with age, in a sample spanning from 18–82 years of age in a perceptual task. Three studies explored cohorts from 18–79 years of age and reported either no influence of age in any of the DDM parameters [23], increases in *non-decision time* with increasing age [24] or increases in *non-decision time* and decreases in *drift rate* with age [25], all in visual feature search tasks. Bolam and colleagues (2024) [26], in a cohort spanning 18–87 years of age, reported negative associations between *drift rate* and age across all sensory conditions in a perceptual (audiovisual) decision-making task while reporting positive associations for *boundary separation* and *non-decision time* in specific conditions only. A recent study using a cohort of 68 participants spanning in age from 24–80 years old showed negative associations between age and *drift rate* and positive associations between age and *boundary separation* in a visual working memory task [27]. Finally, Kelber and colleagues (2025) [28] used an extended version of the DDM in a larger cohort of 1800 participants divided into 6 age groups between 21 and 80 years of age to show that *drift rates* decreased with age while *boundary separation* and *non-decision time* increased in a cognitive control conflict task. In this preregistered study (https://osf.io/yhn4m), we used the DDM to model the performance of three different tasks – *emotion recognition*, *memory*, and *delay discounting* – focusing on a cohort of older adults from middle age to older age (53–95 years of age), using a continuous measure of age and, importantly, in a cohort including participants across the continuum of aging, including the youngest old (conventionally, 65–74) and the oldest old (over 85). We primarily evaluated the effects of age on the *drift rate* parameter, as it reflects the rate of information accumulation during decision-making. Although *drift rate* provides a common mathematical parameter of information accumulation across the three tasks, the nature of the information being accumulated varies with task: perceptual/social information in the *emotion recognition* task, mnemonic information in the *memory* task, and information related to subjective valuation in the *delay discounting* task. Secondarily, we also looked at such effects on the other two parameters, *boundary separation* and *non-decision time*. We also assessed relationships with traditional RT-based processing speed measures and these parameters, to examine why older adults are generally slower in responding to tasks and to validate the proposed relationship between DDM parameters and more conventional measures of processing speed. Finally, given the hypothesis that processing slowing with age is due to age-related changes in white matter microstructure, we assessed the relations among age, white matter measures, and these parameters.

Our preregistered hypotheses were: i) In healthy older adults, age is negatively associated with *drift rate*. (Preregistered ancillary analyses were also conducted to examine associations between age and *boundary separation* and *non-decision time.)* ii) In healthy older adults, conventional RT-based measures of processing speed are positively associated with *drift rate*. (Preregistered ancillary analyses were also conducted to examine associations between RT-based measures and *boundary separation* and *non-decision time.)* iii) Exploratorily, if age is associated with any drift diffusion parameter this association is partially accounted for by changes in white matter microstructure.

## Materials and methods

### Participants

Neurologically healthy, community-dwelling older adults (53−95 years old) were recruited from a cohort enrolled in the Brain Aging Network for Cognitive Health (BRANCH) study at the Memory and Aging Center, University of California, San Francisco. This cohort includes in-person behavioral and neuroimaging measurements and online web-based behavioral tasks. Participants are verified as neurologically normal based on a multidisciplinary assessment, including neurological examination and neuropsychological and cognitive testing. During a consensus conference, physicians and clinicians used the patient history (derived in neurologic exam), informant rated Clinical Dementia Rating (CDR), neuropsychological test results, brain Magnetic Resonance Imaging (MRI), and all available fluid and positron emission tomography (PET) biomarkers to establish the diagnosis. Exclusion criteria for the BRANCH cohort include history or current evidence of: large vessel stroke, diagnosis of DSM-5 major psychiatric disorders, multiple sclerosis, symptomatic neurodegenerative disease, epilepsy, significant memory concerns or related diagnoses, active substance abuse, hepatitis C, HIV, syphilis, blindness, or deafness.

### Study design

Task data for this study were collected online. Members of the cohort received emails through Qualtrics, an online task platform, inviting them to participate in the online instruments. One included a *delay discounting* task, another included an *emotion recognition* task, and another included a verbal *memory* task. Available participant data for each experimental task is as follows: n = 167 for the *delay discounting* task, n = 195 for the *emotion recognition* task and n = 173 for the *memory* task. Measures underlying the RT-based speed composite (described below) were acquired in person as part of the neuropsychological testing for all participants included in the BRANCH cohort.

Given that online data collection for the experimental tasks in this cohort was separate from in-person standard neuropsychological testing (including those included in the RT-based speed composite) and neuroimaging acquisition, participants’ experimental task responses were linked to available data from in-person neuropsychological and neuroimaging testing. These in-person measures are collected on each participant in the cohort at roughly 2-½-year intervals however, due to the outbreak of the world pandemic of COVID-19, our study data include longer gaps between online and in-person data collection. Linkage of associated neuropsychological tests was limited to those acquired within three years of experimental task performance, and linkage of neuroimaging measures was limited to those acquired within four years of experimental task performance.

The Brain Aging Network for Cognitive Health study was reviewed and approved by the UCSF Committee on Human Research (IRB number: 10–02076). The present study was conducted in full compliance with the ethical principles set forth by the Declaration of Helsinki and all participants provided written informed consent. Data for this study was first accessed on September 8^th^, 2022. Investigators had access to the Personal Identification Numbers of each participant, needed to link experimental task data to neuroimaging and neuropsychological data.

### RT-based speed composite

As mentioned above, RT-based measures of processing speed are acquired as part of neuropsychological testing for all participants included in the Brain Aging Network for Cognitive Health. The RT-based speed composite includes six computerized, visuospatial processing speed tests, normalized using a healthy young adult reference group, as detailed by Kerchner et al. (2012) [2]. The resultant measure, calculated as a z-score relative to young healthy controls, reflects response latencies, with higher values corresponding to higher response times and thus slower speed. The normalization to a young adult reference group was retained to maintain consistency with the validated composite measure described by Kerchner et al. (2012) [2], providing a standardized metric of processing speed rather than an age-adjusted measure of impairment. The six tests used to calculate this composite measure all require binary decisions and include Length Judgment, Visual Search, Distance Judgment, Abstract Matching 1, Abstract Matching 2, and Shape Judgment tests [2,29].

### MRI acquisition

As part of the BRANCH study, Magnetic Resonance Imaging (MRI) scans were acquired at the UCSF Neuroscience Imaging Center using a 3T Siemens Magnetom Prisma scanner. T1-weighted images were acquired using magnetization-prepared rapid acquisition with gradient echo acquisition (MPRAGE) with the following parameters: 160 sagittal slices, isotropic voxel size = 1mm^3^, repetition time (TR)/echo time (TE)=2300/2.9ms; inversion time = 900 ms; flip angle = 9°; field of view (FoV) read of 256 mm. A single-shot spin-echo echo-planar imaging (SE-EPI) sequence was used to acquire a diffusion weighted imaging (DWI) sequence with 69 contiguous axial slices within-plane resolution = 2 × 2mm^2^, slice thickness of 2 mm, TR/TE = 2420/72.20ms, flip angle = 85°, FoV of 220 × 220mm^2^, 96 non collinear diffusion sensitization directions at b = 2500 s/mm^2^, 10 additional volumes at b = 0. Additionally, to perform distortion correction, two volumes were acquired at b = 0 with opposite phase-encoding directions (anterior/posterior and posterior/anterior).

All MR were visually inspected to assess image quality or the presence of anomalies, and images with excessive motion or other artifacts were excluded.

### MRI pre-processing

Initial preprocessing of the diffusion data was performed using the FMRIB Software Library (https://fsl.fmrib.ox.ac.uk/fsl/fslwiki/). Susceptibility-induced off-resonance fields were estimated from the two volumes acquired in the opposite phase encoding directions [30] for each participant. Eddy current distortion and motion correction were then performed by registering each diffusion volume to the first b = 0 volume using affine transformations. Single-subject tensor maps were generated and spatially normalized to MNI space with a tensor-based registration algorithm [31,32] using DTI-TK (https://dti-tk.sourceforge.net/pmwiki/pmwiki.php). After tensor diagonalization, fractional anisotropy (FA) and mean diffusivity (MD) maps were obtained for each subject in native space. Average values of FA and MD were extracted from the maps using the white matter mask obtained from the segmentation of WM tissues from the structural images using the Unified segmentation method in SPM12 [33]. Structural and diffusion maps were co-registered using the epi_reg function in FSL with the b0 image as reference, which provides a good alignment for intra-subject registration. Each diffusion map was then normalized to MNI space with the same affine transformations as used for the tensor realignment [34]. A custom template was used for this step to take into account our aging cohort. All the pre-processed images were visually checked for registration errors. After looking at the reports generated by the FSL QUAD and SQUAD tools (https://fsl.fmrib.ox.ac.uk/fsl/fslwiki/eddyqc/UsersGuide), participants with mean displacement greater than 1 mm were excluded from the analysis. Among the retained participants mean displacement was 0.52 ± 0.37 mm.

### Experimental tasks

In the *delay discounting* task, participants performed an intertemporal choice task involving 92 rounds of choices between smaller immediate rewards ($2.50-$38.25) and larger delayed rewards ($5-$40, 4 days to 11 months). Within trials, three parameters were varied independently: percent penalty (the relative difference in magnitudes between the smaller immediate and larger delayed reward), delay length (time required to wait for the delayed reward), and absolute reward magnitudes (expressed in terms of the delayed reward magnitude). Participants were asked to choose between a smaller reward with an immediate payout (smaller soon – SS response) and a larger reward with a delayed payout (larger later – LL response). After selecting the chosen reward in that round, the next round was presented. As a control condition to ensure task comprehension, 12 trials were interspersed throughout the task either asking participants to correctly indicate which reward would be paid out sooner, or asking participants to correctly indicate which reward was the larger reward. Participants received a real monetary incentive based upon their choice in one randomly selected trial. Trial order was fixed for every participant, there was no constraint of time to answer and no fixation between trials (each trial started as soon as the participant answered the previous trial). Instructions to the participants were as follows: ‘We are interested in how people make decisions about money now and in the future. As a token of our appreciation, you will receive an Amazon gift card worth at least $5. We will ask you 104 questions about rewards that you could receive, either today or in the future. There are three types of questions... ‘Which reward is paid SOONER?’ ‘Which reward is LARGER?’ ‘Which reward do you WANT?’ Most of the questions will be about which reward you WANT. At the end, we will randomly choose one of these questions to count for real money. You will receive the reward (either today or in the future) that you chose.’

For the *emotion recognition* task, validated images of facial expressions were morphed [35] for anger, disgust, fear, happiness, sadness, and surprise to produce samples ranging from neutral to each fully expressed emotion in 5% increments. For each of the 160 trials, participants were presented with a 2-choice discrimination task in which a morphed sample of varying intensity was displayed on the screen, and they were asked to identify which emotion the model expressed, choosing between the correct response and a lure (with positions randomized between left and right). The subsequent trial difficulty was adaptively tuned by increasing or decreasing the intensity of the expressions presented, using a one-up, two-down staircase algorithm to ensure performance across individuals ranging from 66–70% accuracy, reversal occurred after 1 incorrect response or 2 correct responses in a row. Starting difficulty was fixed at 30 on a scale of 5–95 and the step size was fixed at 5, with no separate staircases by emotion. Stopping was not based on reversal counts but once 160 trials had been reached. Emotions were superimposed on 10 unique faces and the number of trials for each emotion was 28 for anger, 26 for happiness, 28 for surprise, 24 for disgust, 25 for sadness and 29 for fear. The emotion shown on each trial was randomized, there was no constraint of time to answer and no fixation between trials (each trial started as soon as the participant answered the previous trial). Instructions to the participants were as follows: ‘This is a study about recognizing emotions. As a token of our appreciation, you will receive a $15 Amazon gift card. This task has multiple trials, and each trial will begin with a choice like this: You’ll click on which emotion you think the model is expressing. You will answer these questions about 160 faces with different expressions. Some of the emotions may be easy for you to identify, and some may be hard for you to identify. Please be sure to try your best throughout the experiment! Let’s try a few practice rounds before we begin. Click the button below when you are ready to start.’

The *memory* task was based on the McCurdy et al. (2013) [36] task; lists of words from the Medical Research Council Psycholinguistic Database [37] were generated, containing 4–8 letters and 1–3 syllables, and familiarity, concreteness, and imageability ratings between 400–700. Participants viewed a list of words ranging from 6–20, with a duration spanning from 8–28 seconds to memorize the presented list (study slide). Following the time allotted to memorize the word list, participants had to wait another 2–22 seconds (wait slide) before answering six 2-alternative forced-choice recognition questions per list. Study slide and wait slide were always 30 seconds combined. Each question contained a word from the list participants saw, and the other word was a lure (with positions randomized between left and right). The difficulty of word lists presented to subjects was adaptively tuned based on how many out of the six word recognition questions for the preceding list were correctly answered. Two features of the subsequent word list were increased or decreased: study time and the number of words presented, to maintain participant accuracy around 66–70%. Regarding study time, for more than 4 correct responses, there was no change in study time. For 5 or 6 correct responses, study time decreased by 4 seconds. For less than 4 correct responses, study time increased by 4 seconds. If study time dropped below 12 seconds, then word count increased by one level and study time was reset to 16 seconds. If study time went above 20 seconds, word count went down one level and study time was reset to 16 seconds. In cases where a participant had all 6 correct responses on a current trial and an overall accuracy of > 70% for all trials or, inversely, less than 3 correct responses on current trial and an overall accuracy of < 60%, the step value was doubled. Delay time was adjusted according to the increase or decrease in study time to keep the combined time at 30 seconds. Participants answered a total of 144 2-alternative forced-choice questions. There was no constraint of time to answer and no fixation between trials (each trial started as soon as the participant answered the previous trial). Instructions to the participants were as follows: ‘This is a study about memory. As a token of our appreciation, you will receive a $15 Amazon gift card. Please scroll through to the bottom of this page to view the structure of this task. Once you reach the bottom of the page, you will find a button to proceed to the next page. This task has multiple trials. For each trial, you will receive an allotted amount of time to memorize the list of words displayed. For example: Next, you’ll wait for an amount of time before answering a series of questions about the list of words. Once the waiting time has concluded, we will test your memory for some of the words you have just memorized. We will show you two words at a time, one of which was on the list you just memorized and one that was not. You will answer questions about 24 different lists of words. Some of the lists may be easy to remember, and some may be hard to remember. Please be sure to try your best throughout the experiment! Let’s try a few practice rounds before we begin. Click the button below when you are ready to start.’

### Statistical analyses

Data were collected from participants at home; because the setting of testing was unconstrained, there is a particular concern that trials with particularly slow RTs may represent environmental distractors or other off-task processes. Therefore, before fitting the DDM and performing any statistical analysis, for each participant the slowest 5% responses in each task were discarded. Moreover, since fast outliers can be more problematic than slow outliers [38,39], for each participant, trials with RT faster than 350ms were also discarded.

Power calculations were performed prior to any analysis, as described in the study preregistration (https://osf.io/yhn4m). All statistical analyses were conducted using R 4.1.0 [40] except for the tract-based spatial statistics (TBSS) analysis, which was conducted in FSL [34].

Because our preregistered primary hypothesis was evaluated across three tasks, p-values for the *drift rate* analyses were adjusted for multiple comparisons using the Benjamini–Hochberg false discovery rate (FDR) procedure. Associations involving *boundary separation* and *non-decision time* were considered ancillary analyses and are reported as effect sizes with 95% confidence intervals.

#### Drift diffusion model fitting.

In the context of value-based decision-making tasks, while these do not have an external or objective standard of “correctness” as in perceptual or memory decisions, one accepted approach is to analogize correct choices to those that maximize a subjective value/utility function. Different subjects have different temporal preferences and so, for the *delay discounting* task, before fitting the DDM we applied maximum likelihood estimation (MLE) to the hyperbolic discounting model, V = A/(1 + kD), to determine a parameter *k* for each subject that reflects the degree of discounting the value of a future reward (A) depending on the delay (D), yielding the present value attributed to the reward (V) [41]. We used the optim() function from base R to perform the MLE, using the Brent method, often suited for univariate optimization problems, setting the boundaries to very small and very large values (-1e10, 1e10 for lower and upper boundaries respectively) to avoid imposing constraints on the numerical optimization. Although this allowed negative *k* estimates, inspection of the estimated values showed that 25 negative estimates were extremely small (roughly 10^-15^) and were considered numerical approximations to zero, while two participants had negative estimates (*k* = −0.019). The larger the value of *k*, the larger the effect of delay in reducing the present value of a future reward [41]. We then coded the responses for this task as consistent or inconsistent with the predictions of the hyperbolic model given the subject specific parameter *k*. Therefore, we conceptualize *drift rate* in this case to reflect the evidence accumulation towards the subject’s preferred response given their individual overall tendency to weigh future and present rewards. However, because this tendency was estimated from the same choices subsequently classified as consistent or inconsistent based on the estimated *k*, the resulting *drift rate* could differ from that obtained when response accuracy is defined independently of participants’ choices.

We fitted DDMs to RT distributions, obtaining estimates of diffusion model parameters separately for each participant and each experimental task. For parameter estimation, the optimization criterion Maximum Likelihood (ML), implemented in the program *fast-dm-30* [39,42,43], was used. A simple model was fitted, estimating *drift rate* (*v*), *boundary separation* (*a*), *non-decision time* (*t0*), and the *inter-trial variability of non-decision time* (*st0*). This last parameter was estimated given its impact on the shape of the RT distribution [39]; however, these parameter estimates were not used for subsequent between-subject analyses. Since no a priori bias regarding responses was predicted, the starting point was fixed at the center between the two boundaries. The upper and lower boundaries were associated with consistent and inconsistent responses for the *delay discounting* task and with correct and error responses for both the *emotion recognition* and the *memory* tasks, respectively. All other parameters were set to zero.

Tasks with slow RT have not often been analyzed with the DDM, and so a thorough investigation of model fit is necessary. Graphical displays of model fit are important, but its interpretation can be ambiguous since there is no clear criterion on which to exclude participants considered to have a bad model fit [38]. We then chose to use a double-process of inspecting model fit to ensure that the model fit our data, applying an objective criterion for excluding participants. We first assessed the model fit using Monte Carlo simulations. Following Voss et al. (2013, 2015) [38,39], for each task we first created 1000 random parameter values from a multivariate normal distribution defined by the mean values and the variance-covariance matrix of the previously estimated DDM parameters, to ensure that the simulated data would match the characteristics of our empirical data. Then, for each of these 1000 parameter values we generated one random data set using each of these parameter values and the same trial number present in our tasks (using the *construct-samples* tool from *fast-dm*-*30*). Finally, parameter estimates for these simulated data sets were obtained by using again *fast-dm-30* to fit a diffusion model, using the same settings previously used for the empirical data. By doing this we obtained fit-values for models that were based on data following a diffusion process [38]. A distribution of fit-values is provided by *fast-dm*, with higher likelihood values indicating a better fit. Following Lerche et al. (2020) [6] and Voss et al. (2013) [38], we took the 1% percentile of this distribution as a critical value to assess model fit of the empirical models. For each task, participants with poor model fit – participants with fit values worse than this 1% criterion – were excluded from the subsequent analyses. We also performed a graphical check by plotting predicted values against empirical values for the response consistency/accuracy and for the 0.1, 0.25, 0.50, 0.75 and 0.9 quantiles of RT distributions. Lastly, parameter recovery was assessed by comparing the empirical DDM parameter estimates (*drift rate*, *boundary separation* and *non-decision time*) with those re-estimated after fitting the model to simulated datasets generated from the original parameter estimates, using Spearman correlations, to assess preservation of the rank ordering of parameter values.

#### Behavioral analyses.

For our preregistered hypothesis i and ii, the association between age and *drift rate* and between the RT-based speed composite and *drift rate* was tested by computing the bivariate correlations (Spearman correlations) between these two pairs of variables, separately for each task. Spearman’s correlation was selected a priori as a non-parametric approach to assess monotonic associations without assuming linear relationships. Because each primary hypothesis was evaluated across three tasks, p-values for 6 associations were adjusted for multiple comparisons using the Benjamini–Hochberg false discovery rate (FDR) procedure. Additionally, we examined the association between age and the other two diffusion model parameters (*boundary-separation* and *non-decision time*) and between the RT-based speed composite and these two parameters, as prespecified ancillary analyses. These ancillary analyses were not included in the FDR correction, as they were treated as ancillary and are reported descriptively as effect sizes with 95% confidence intervals, without p-values or significance-based inference. A sensitivity analysis was performed without the exclusion of the 5% slowest RT to check for the robustness of our results. Moreover, for the *delay-discounting* task, a sensitivity analysis for the associations of *drift rate* with age and the RT-based speed composite was performed after excluding the two participants with *k* = −0.019. Additionally, we conducted exploratory pairwise Spearman correlations between *drift rate* estimates from the three tasks to assess the degree of correspondence in *drift rate* across task contexts. These are reported descriptively as effect sizes with 95% confidence intervals. As a further check, to assess the potential influence of the adaptive difficulty procedures of the *emotion recognition* and the *memory* tasks on *drift rate*, we examined the distributions of achieved task difficulty and its associations with age and *drift rate* for both tasks. These are reported as effect sizes with 95% confidence intervals. For the *memory* task, although both word count and study time were adjusted by the adaptive procedure, all participants reached the maximum word count. Therefore, individual differences in final achieved difficulty were characterized using final study time, with shorter study times indicating greater difficulty. In a sensitivity analysis, we performed partial Spearman correlations between age and *drift rate* while controlling for achieved task difficulty. These associations were adjusted for multiple comparisons using the Benjamini–Hochberg FDR procedure correcting for the same number of associations as in the main analyses).

To explore whether white matter changes can account for age effects on *drift rate,* when significant associations were found between these two variables mediation models were created, separately for each task. In these mediation models, age was the primary predictor variable and *drift rate* was the outcome variable. The difference (in days) between DTI acquisitions and task performance was used as a covariate. White matter microstructure measures Fractional Anisotropy (FA) and Mean Diffusivity (MD) within the total white matter were used as the mediators in these models. One single FA and one single MD value were extracted per participant by averaging the FA and MD over all the voxels within the white matter. P-values for these exploratory analyses were adjusted for 4 comparisons using the Benjamini–Hochberg FDR procedure. The *mediation* package for R was used to perform all mediation analyses [44].

To assess the potential influence of intervening changes in clinical status during the time gap between the online task evaluation and the RT-based speed composite and neuroimaging acquisitions, sensitivity analyses for hypothesis ii and exploratory hypothesis iii were performed including only participants for whom a subsequent longitudinal evaluation following completion of the online tasks was available and confirmed that they remained cognitively and neurologically healthy. All sensitivity analyses followed the same FDR adjustments for multiple comparisons as the main analyses. All p-values are reported after adjustment for multiple comparisons unless explicitly stated otherwise.

To directly assess the potential influence of temporal mismatch between measurements, additional sensitivity analyses were performed utilizing progressively narrower time windows for linkage to in-person neuropsychological and neuroimaging testing. For analyses involving the RT-based speed composite, these time windows were ≤1 year, ≤ 2 years, and (as originally preregistered) ≤3 years; for analyses involving neuroimaging measures, these time windows were ≤1 year, ≤ 2 years, ≤ 3 years, and (as originally preregistered) ≤4 years. These are presented graphically with Confidence Intervals.

#### Tract-based spatial statistics (TBSS).

In addition to our preplanned main and exploratory analyses, we performed voxel-wise statistics of the diffusion tensor data using TBSS. Briefly, the previously created single-subject FA maps were used to generate a mean FA image of the full sample, which was then thinned to create the mean FA skeleton with a threshold of 0.2, representing the common white matter tracts across all participants [34]. Each participant’s aligned FA map was projected onto this skeleton, and the resulting data was fed into voxel-wise cross subject statistics, using FSL *randomise* [45], to examine the effects of age on FA and the effects of FA on each DDM parameter. The same procedure was used to analyze the effects of age on MD and the effects of MD on the DDM parameters. For the effects of age on FA and MD the full sample was used in the model (N = 171). For the analysis regarding the effects of FA and MD on the DDM parameters, age and the difference (in days) between DTI acquisitions and task performance were used as covariates. A value of *p <* 0.001, corrected, based on 5000 permutations and threshold-free cluster enhancement was set as the threshold for significance [46].

## Results

Three participants were excluded due to missing demographic data (two for the *delay discounting* task and one for the *memory* task). Descriptive statistics of the final sample included in each task are provided in Table 1. For the subsample that completed the *delay discounting* task, participants’ mean age was 76.8 years old, 77.1 years old for the *emotion recognition* task and 77.5 years old for the *memory* task. For each of the three tasks there were ~13 middle-aged adults, ~ 47 youngest-old adults, ~ 90 middle-old adults and ~26 oldest-old adults. The study sample was highly educated (17.4 years on average) and predominantly White and non-Hispanic (~86%).

**Table 1:** Demographic variables for participants included in each task; means (standard deviation) provided for age and education.

Demographics	Delay discounting (N = 164)	Emotion recognition (N = 194)	Memory (N = 171)
Age (years)	76.77 (7.59)	77.09 (8.06)	77.47 (7.7)
Middle-age (45–64), N	11	16	12
Youngest-old (65–74), N	46	51	45
Middle-old (75–84), N	88	96	85
Oldest-old (85+), N	19	31	29
Female Sex	58%	60%	59%
Education (years)	17.31 (2.14)	17.39 (1.94)	17.36 (2.01)
Race			
Hispanic	4.9%	4.2%	4.1%
White, non-Hispanic	87%	85%	86%
Asian, non-Hispanic	6.7%	8.3%	7.7%
Black, non-Hispanic	1.2%	1.6%	1.8%
Other, non-Hispanic		0.5%	

Given the exclusion of the 5% slowest trials for each participant in each task, the final number of trials was as follows: 87 trials for the *delay discounting* task, 152 trials for the *emotion recognition* task and 136 trials for the *memory* task. No participants had RTs shorter than 350ms in any of the tasks. Visual inspection confirmed that the characteristic positively skewed RT distributions were preserved after the exclusion of the 5% slowest trials (S1 Fig).

### Diffusion model fit

After examination of model fit based on the Monte Carlo simulations, two participants were excluded for the *delay discounting* task and one participant was excluded for the *emotion recognition* task. No participants showed a poor model fit for the *memory* task (S2 Fig). Graphical check of model fit also suggested a good fit for our data, with most data points positioned near the main diagonal for all estimated quantiles (0.1, 0.25, 0.50, 0.75 and 0.9) of the empirical and predicted RT distributions and for accuracy (S3 Fig). Parameter recovery analyses further demonstrated that the rank ordering of the estimated DDM parameters was well preserved across all three tasks, with strong correlations between the original and re-estimated values for *drift rate* (ρ = 0.84–0.95), *boundary separation* (ρ = 0.93–0.98), and *non-decision time* (ρ = 0.96–0.97) (all p < 0.001) (S4 Fig).

Mean DDM parameter values and mean RT for each task are displayed in Table 2 and S5 Fig. S6 Fig shows mean DDM parameter values per age group (age groups can be found in Table 1 – demographic table). S7 Fig shows the pairwise Spearman correlations between *drift rate* estimates from the three tasks.

**Table 2:** Means (± standard deviations) of mean drift-rate, boundary-separation, non-decision time (in seconds), RT (in seconds) and accuracy rates (%) for each experimental task. For the *delay discounting task,* ‘consistency’ represents the % of responses that were consistent with the hyperbolic discounting model given the participant’s estimated *k* (discount rate) value.

	Drift rate	Boundary separation	Non-decision time (s)	Reaction time (s)	Consistency / Accuracy (%)
**Delay discounting (N = 162)**	0.73 (0.43)	3.79 (1.61)	2.24 (1.13)	4.65 (2.47)	88.06 (7.79)
**Emotion recognition (N = 193)**	0.29 (0.11)	3.71 (1.14)	3.73 (1.15)	6.9 (2.14)	72.64 (1.47)
**Memory (N = 171)**	0.65 (0.23)	2.15 (0.62)	3.04 (0.74)	4 (0.81)	77.84 (4.84)

### Association between age and *drift rate* (hypothesis i)

The final sample size was N = 162, N = 193 and N = 171, for the *delay discounting*, *emotion recognition* and *memory tasks*, respectively.

There was no association between age and response consistency for the *delay discounting* task (ρ = − 0.056, p = 0.48) and there was a negative association between age and accuracy for the *emotion recognition* (ρ = −0.263, p < 0.001) and the *memory tasks* (ρ = −0.23, p = 0.002).

Age was significantly negatively associated with *drift rate* for both the *emotion recognition* (ρ = −0.19, p = 0.016) and the *memory* tasks (ρ = −0.18, p = 0.026) (Fig 1 and Table 3). Additionally, age was positively associated with *boundary separation* in the *emotion recognition* task (ρ = 0.16, 95% CI [0.017–0.29]), whereas little evidence of an association was observed in the other tasks. Age was also positively associated with *non-decision time* across all three tasks (*delay discounting:* ρ = 0.33, 95% CI [0.19–0.46]; *emotion recognition*: ρ = 0.37, 95% CI [0.24–0.49]; *memory*: ρ = 0.39, 95% CI [0.25–0.51]) (Table 3 and S8 Fig). S1 Table shows results for the sensitivity analysis, without excluding the 5% slowest trials. Results remain mainly unchanged. Excluding the two participants with clearly negative *k* estimates did not noticeably alter the *delay-discounting* task results (ρ = −0.097 against ρ = −0.098, p = 0.27).

**Table 3:** Spearman correlations – rho (95% Confidence Interval) [p-value] – between age and the drift diffusion model parameters and between age and mean RTs for each task. P-values for *drift rate* are Benjamini–Hochberg FDR-adjusted.

	Drift rate	Boundary separation	Non-decision time	Reaction time
**Delay discounting (N = 162)**	−0.098 (−0.25, 0.057) [0.25]	0.075 (−0.08, 0.23)	0.33 (0.19, 0.46)	0.23 (0.079, 0.37)
**Emotion recognition (N = 193)**	−0.19 (−0.32, −0.052) [0.016]	0.16 (0.017, 0.29)	0.37 (0.24, 0.49)	0.37 (0.24, 0.49)
**Memory (N = 171)**	−0.18 (−0.32, −0.033) [0.026]	0.043 (−0.11, 0.19)	0.39 (0.25, 0.51)	0.4 (0.27, 0.52)

**Fig 1:**
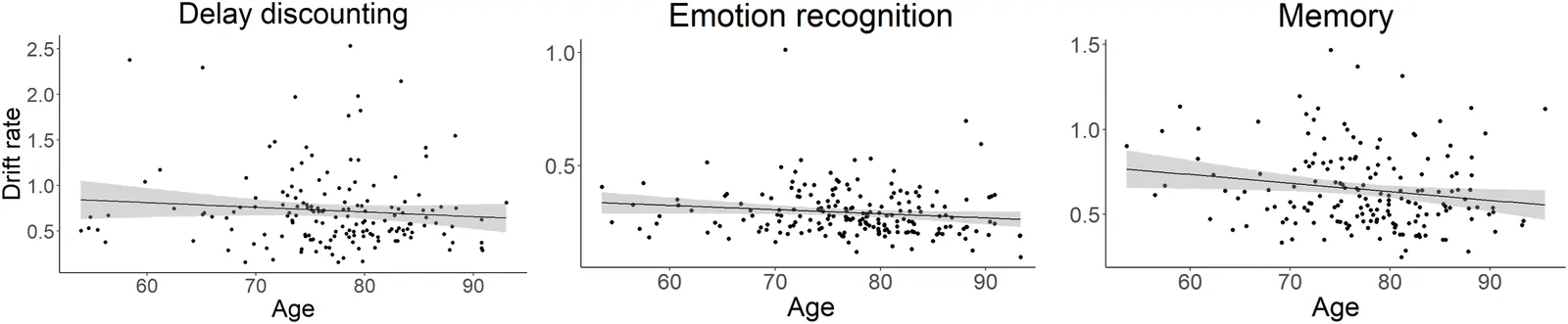
Plots for the association between age and *drift rate* for the *delay discounting*, *emotion recognition* and *memory* tasks. Spearman correlations were computed, and associations were statistically significant at an FDR-adjusted p-value of 0.05 using the Benjamini–Hochberg procedure for the *emotion recognition* and *memory* tasks.

Given the adaptive nature of the *emotion recognition* and *memory* tasks, we additionally examined whether the task difficulty achieved by participants was associated with age and *drift rate*. In both tasks, lower values of the adaptive parameter represented greater task difficulty. In the *emotion recognition* task, age was positively associated with the final adaptive level (ρ = 0.21, 95% CI [0.08,0.35]) whereas *drift rate* was negatively associated with the final adaptive level (ρ = −0.15, 95% CI [−0.29, −0.01]). In the *memory* task, the association between age and final adaptive level was ρ = 0.12 (95% CI [−0.03, 0.27]), while *drift rate* was negatively associated with the final adaptive level (ρ = −0.24, 95% CI [−0.38, −0.10]). In sensitivity analyses, partial Spearman correlations controlling for achieved task difficulty showed that the negative association between age and *drift rate* remained significant for both *emotion recognition* (ρ = −0.16, p = 0.044) and *memory* (ρ = −0.16, p = 0.048) tasks. The distributions of achieved task difficulty are displayed in S9 Fig.

### Association between the RT-based speed composite and *drift rate* (hypothesis ii)

After excluding participants that did not have the in-person neuropsychological measures underlying the RT-based speed composite acquired within three years of experimental task performance, the final sample sizes to compute associations between the RT-based speed composite and drift diffusion model parameters were N = 157, N = 106 and N = 95 for the *delay discounting*, *emotion recognition* and *memory* tasks, respectively. The RT-based speed composite was significantly negatively associated with *drift rate* for both the *delay discounting* (ρ = −0.274, p = 0.003) and the *memory* tasks (ρ = −0.271, p = 0.016). In addition, a positive association was found between the RT-based speed composite and *boundary separation* for the *delay discounting* (ρ = 0.272, 95% CI [0.12–0.41]) and the *memory* tasks (ρ = 0.223, 95% CI [0.022–0.41]), and the association between the RT-based speed composite and *non-decision time* was positive across all three tasks (*delay discounting*: ρ = 0.19, 95% CI [0.03–0.33]; *emotion recognition*: ρ = 0.33, 95% CI [0.15–0.49]; *memory*: ρ = 0.25, 95% CI [0.047–0.43]) (Table 4). S2 Table shows results for the sensitivity analysis, without excluding the 5% slowest trials. Excluding the two participants with clearly negative *k* estimates did not noticeably alter the *delay-discounting* task results (ρ = −0.27, p = 0.005). S3 File shows results for the sensitivity analysis, including only participants for whom a subsequent longitudinal evaluation was available following completion of the online tasks, at which they were confirmed to remain cognitively and neurologically healthy. In this sensitivity analysis, associations between the RT-based speed composite and *drift rate* were attenuated relative to the primary analyses for the *delay discounting* and *memory* tasks, while the association for the *emotion recognition* task was weak and changed direction. Confidence intervals were also wider, reflecting the substantially reduced sample sizes, and none of the associations were statistically significant. S10 Fig demonstrates the sensitivity analysis focusing on temporal mismatch between task performance and in-person neuropsychological testing for the elements included in the RT-based speed composite. Because of restrictions on in-person evaluation during the COVID-19 pandemic, no participants had in-person evaluations within 1 year of the emotion recognition or memory tasks and few had in-person evaluations within 2 years of these tasks.

**Table 4:** Spearman correlations – rho (95% Confidence Interval) [p-value] – between RT-based speed composite and the drift diffusion model parameters and between RT-based speed composite and mean RTs for each task. P-values for *drift rate* are Benjamini–Hochberg FDR-adjusted.

	Drift rate	Boundary separation	Non-decision time	Reaction time
**Delay discounting (N = 157)**	−0.27 (−0.41, −0.12) [0.003]	0.27 (0.12, 0.41)	0.19 (0.03, 0.33)	0.41 (0.28, 0.54)
**Emotion recognition (N = 106)**	−0.094 (−0.28, 0.099) [0.34]	0.054 (−0.14, 0.24)	0.33 (0.15, 0.49)	0.27 (0.08, 0.44)
**Memory (N = 95)**	−0.27 (−0.45, −0.073) [0.016]	0.22 (0.022, 0.41)	0.25 (0.047, 0.43)	0.42 (0.24, 0.57)

### Mediation models and tract based spatial statistics (exploratory hypothesis iii)

After excluding participants that did not have DTI measures acquired within four years of experimental task performance, and after exclusion of participants with images with excessive motion or other artifacts, the final sample sizes for the mediation analysis were N = 109, N = 104 and N = 96 for the *delay discounting*, *emotion recognition* and *memory* tasks, respectively.

An exploratory indirect association involving global FA was observed between age and *drift rate* in the *emotion recognition* task. Unstandardized indirect effects were computed for each of 10000 bootstrapped samples. The bootstrapped unstandardized indirect effect was −1.69e-3 (uncorrected p = 0.036) but did not remain statistically significant after correcting for multiple comparisons (p = 0.134). Neither global FA nor global MD showed significant indirect effects in the associations between age and *drift rate* for any other experimental task. For the sensitivity analysis, including only participants for whom a subsequent longitudinal evaluation was available following completion of the online tasks, at which they were confirmed to remain cognitively and neurologically healthy, the sample size was reduced to N = 77 and bootstrapped unstandardized indirect effect was −3.3e-3 (p = 0.003), after correcting for multiple comparisons. S11 Fig demonstrates the sensitivity analysis focusing on temporal mismatch between task performance and in-person neuroimaging; the direction and magnitude of indirect effects were not substantially changed by the use of narrower time windows.

Voxel-wise analysis showed, as expected, a decrease in FA in various white-matter tracts – left and right corticospinal tract, left and right internal and external capsules, left and right anterior thalamic radiation, left inferior fronto-occipital fasciculus, left superior an inferior longitudinal fasciculus, forceps minor and body of corpus callosum – and an increase in MD with increased age in widespread white matter regions (Fig 2). This voxel-wise analysis also showed a positive association between MD and *non-decision time* in the *delay discounting* task in several white-matter tracts, including the left and right anterior thalamic radiation, left and right corticospinal tract, the cingulate gyrus, the forceps major and minor, left and right inferior fronto-occipital fasciculus, left and right inferior and superior longitudinal fasciculus, and the left and right uncinate fasciculus (S12 Fig). No other significant associations were found between regional FA nor regional MD with any DDM parameters for any of the tasks.

**Fig 2:**
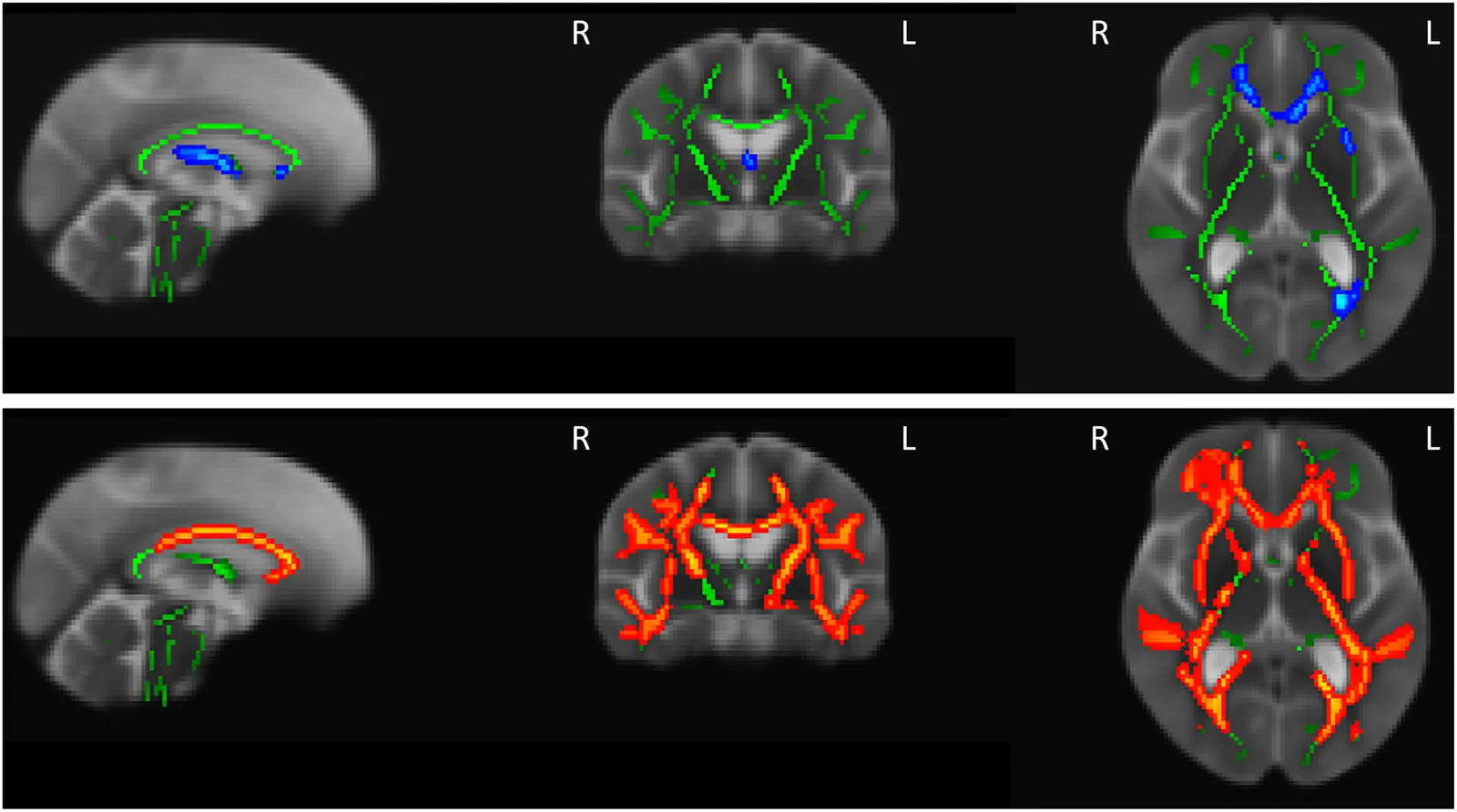
Whole brain voxel-wise analysis for the effects of age on DTI-WM measures: fractional anisotropy (FA) and mean diffusivity (MD). Age correlates negatively with FA (p < 0.001, corrected, threshold-free cluster enhancement) (top row) whereas it correlates positively with MD (p < 0.001, corrected, threshold-free cluster enhancement) (bottom row). The significant areas (blue and orange) are superimposed on both the mean skeleton (green) and mean FA image of the full sample. Results are thickened for ease of visualization. All slices are illustrated in radiological (left-is-right) orientation.

## Discussion

In this preregistered study examining age as a continuous variable in a well-characterized sample of healthy older adults, we found: first, a negative association between age and *drift rate* both in an *emotion recognition* and a *memory* task; second, a negative association between an RT-based speed composite and *drift rate* on a *delay discounting* and a *memory* task; and, finally, our exploratory analysis for the association between age and *drift rate* in the *emotion recognition* task identified an indirect association involving global FA, although this did not remain statistically significant after correction for multiple comparisons.

We showed a negative association between *drift rate* and age for both the *emotion recognition* and the *memory* tasks but not for the *delay discounting* task. The results of our first aim are generally congruent with findings drawn largely from two-group comparisons of older and younger adults, suggesting declines in *drift rate* with age (reflecting slower information accumulation within a given task context) with evidence for this association observed in two of the three cognitive domains examined.

In a two-group comparison of older and younger adults, Chard and colleagues (2022) [47] applied both a signal detection paradigm and the DDM to tasks examining either configural or featural differences in pairs of face stimuli, reporting that older adults had difficulties in detecting changes in faces due to reduced evidence accumulation (*drift rate*). Emotion recognition tasks require processing of configural and featural information in a face to derive emotional contents [48] so our results across a sample of older adults are consistent with these findings, suggesting that older individuals require more time to process face information to distinguish emotions conveyed by faces.

Our results support the belief that age differences in memory tasks may be attributable to age-related changes in cognitive processes such as recollection or associative encoding [14] and are also in line with Spaniol and colleagues’ (2006) [14] results. However, the decrease in *drift rate* in our *memory* task appears incongruent with findings from Ratcliff and colleagues (2007) [13], that used a similar word recognition memory task but showed no differences between a group of college-age, 65–75 years old and 75–90 years old participants. The procedural differences between experiments, particularly regarding on how test stimuli were presented, might in part explain this divergence, since in Ratcliff’s (2007) [13] study the participants had only to recollect if a particular word had been seen or not whereas in our study the task was to discriminate between a target or a lure, which slightly increases task difficulty, a moderator of age differences in *drift rate* [12].

Age was not associated with *drift rate* for the *delay discounting* task, which may suggest that increased RTs in older adults when performing this kind of task are not tied to a slowing in information accumulation, inviting the interpretation that the association between age and *drift rate* is task dependent. However, such interpretation needs to be taken with caution, particularly because the nonsignificant association between age and *drift rate* in this task was in the same direction as the significant association between age and *drift rate* in the *emotion recognition* and *memory* tasks; that one association is nonsignificant and two associations were significant does not demonstrate a significant difference between them. Moreover, because *k* was estimated from the same choices that were subsequently classified as consistent or inconsistent, the resulting *drift rate* may not be equivalent to one estimated from independently defined correct and incorrect responses. Finally, we used a two-stage procedure for this task, applying maximum likelihood estimation to the hyperbolic discounting model to estimate *k* – for posterior response coding – before fitting the DDM, making the *delay discounting* DDM conditional on this first stage. Future work using joint modeling approaches such as hierarchical Bayesian DDMs might lead to more robust conclusions [49,50].

The adaptive procedures used in the *emotion recognition* and *memory* tasks also deserve consideration when interpreting *drift rate*. These procedures were implemented to maintain participant performance within a comparable accuracy range (approximately 66–70%), thereby reducing floor and ceiling effects and maintaining a similar level of task difficulty across individuals with different abilities. Consequently, although participants were not presented with identical stimulus difficulties, *drift rate* should be interpreted as reflecting the efficiency of evidence accumulation under task conditions individually adjusted to achieve comparable levels of performance. The additional analyses showed that participants with higher *drift rates* tended to reach more difficult task conditions in both tasks, confirming that achieved difficulty is associated with individual differences in *drift rate* estimates. Older age was also associated with reaching less difficult conditions in the *emotion recognition* task, although the confidence interval for the association between age and difficulty included zero in the *memory* task. Importantly, however, the negative associations between age and *drift rate* remained significant after accounting for achieved task difficulty in both tasks, suggesting that differences in achieved difficulty alone did not account for the observed age–*drift rate* associations. Nevertheless, because participants were exposed to different task conditions, direct comparison of *drift rate* estimates across individuals should be made with caution.

Most importantly, taking age as continuous variable instead of simply comparing two groups allowed us to observe age-related differences in certain abilities that could otherwise not be seen. Hartshorne and colleagues (2015) [21] performed a study on the peak age for different cognitive functions across the lifespan; while some peak in early adulthood, many peak in mid-life. They note that a study comparing a cohort of college freshmen to a cohort of 65-year-olds on an emotion recognition task would find no differences (as both groups are on either side of the peak), which would support an erroneous conclusion that there are no age-related changes in such tasks. Hence the importance of the approach taken here. To our knowledge, eight studies to date have explored the association between age and *drift rate* in cohorts spanning in age from 60 years old on [20,22–26], with six of them reporting a decrease with age in *drift rate* [20,22,25–28]*.* However, most of these studies included participants until 80 years of age, most with 10 or fewer participants and only one with 20 participants over 70 years-old and 2 participants between 80–87 years-old; the only study with a large cohort divided it into age groups as opposed to using age as a true continuous variable. Moreover, the studies explored only one task each, with two of them being a perceptual task [20,26] and three a visual feature search task, with two studies only looking at higher-level cognitive tasks [27,28]. We add to these studies by exploring this question in a cohort with a larger sample of older adults (~87 participants aged 70–80 years old and ~63 participants older than 80 years old), and in three different tasks and showing a similar decrease of *drift rate* with age in two of them.

An RT-based speed composite was associated with all DDM parameters in both the *delay discounting* task and the *memory* task, and with *non-decision time* for the *emotion recognition* task, suggesting that the drift diffusion model captures the slowing of task performance efficiently and supporting the claims that it allows for the identification of different mechanisms associated with such slowing. However, these associations were attenuated or changed direction in the restricted sensitivity analysis, warranting caution in interpreting these findings, particularly given the temporal separation between assessments.

Finally, our TBSS analysis showed, as expected, widespread age-related differences in white matter microstructure [51,52]. Fractional anisotropy decreased with age whereas mean diffusivity increased. Accounting for the important role of white matter pathways in the transfer of information between brain regions, it is expected that structural alterations of these pathways may affect cognitive processes. Our mediation analysis, with age as the predictor variable, *drift rate* as the outcome and FA or MD as mediator variables identified an exploratory indirect association involving global FA in the association between age and *drift rate* in the *emotion recognition* task. This finding is consistent with the suggestion that information processing speed is likely to be related to global brain connectivity rather than to specific tracts [53] and hence, since white matter is affected in wide-spread regions of the brain, global FA may be particularly relevant for investigating the relationship between age and *drift rate*. However, given that the finding we report did not survive multiple comparisons correction, this result should be interpreted with caution and be seen as exploratory rather than definite, needing further research to reach more precise conclusions.

Two contributions of our study to the literature are (1) modeling of age as a continuous parameter within a common cohort (rather than relying on group-level comparisons across young and old cohorts), and (2) estimation of task-specific DDM parameters across heterogeneous operations within the same cohort and with an overlapping set of demographic, neuropsychological, and neuroimaging measures.

Globally, our results are important in showing that different mechanisms might contribute to age-related slowing of information processing across cognitive contexts and that these task-specific processes should be considered when performing studies on age and cognition to not fall in a simplistic overgeneralization of cognitive decline with age. It is important to acknowledge that these mechanisms are different and that different strategies might be used to increase optimal performance by older adults, depending on the components most affected. For example, the identification of the reasons behind emotion recognition deficits in older adults is of particular concern, given the problems it poses, notably regarding social isolation [47]. Knowing that higher evidence accumulation (*drift rate*) might be needed for older individuals to infer emotions from faces might help train health professionals and other professionals dealing with the older adult population to be more expressive [47] and potentially engage body language when addressing this population. Regarding memory retrieval, our results, along with the results from Spaniol et al. (2006) [14] and Ratcliff et al. (2007) [13] suggest that different encoding and retrieval strategies are associated with differences in older adults’ performance thus, strategies that maximize their ability to recall, such as simplifying tasks by minimizing the stimuli used in a recall phase, might be preferred to those that use different stimuli.

There are some limitations to the present study. The experimental tasks were conducted online, allowing for little control over the conditions in which tasks are performed, a factor that could particularly influence RTs, an important variable for this study. RTs observed in our experiments were high when compared to similar tasks used in other DDM studies. Most studies applying the DDM are conducted in a laboratory setting, often in constrained conditions and with explicit instructions on motor responses, which increases the speed of motor processes [54]. As data collection for our study was conducted online in unconstrained home environments, motor responses would be expected to be slower and more variable than for studies conducted in laboratory settings. Such factors would be reflected in the *non-decision time* parameter. Indeed, as is observed in Table 2, the mean non-decision times in our three tasks are above 2 seconds. Importantly, however, Lerche and Voss (2019) [10] showed that the DDM can be applied to slow-RT tasks, using a paradigm where participants showed mean RT of around 7s. As with this earlier study, we performed a thorough analysis of model fit, using both graphical checks and a simulation study, and we showed that the model fitted our data relatively well, suggesting that online collection of data can be employed in studies for which RTs are the basis of the analysis [55]. A related issue was the exclusion of the slowest 5% of responses, that was intended to reduce the influence of lapses and environmental distractions inherent to unsupervised home testing. However, some of these responses may also reflect genuine age-related variability. Nevertheless, our sensitivity analyses, including all responses, yielded largely comparable results. Another constraint inherent to our at-home testing design is the inability to control for variables such as sleep quality, time-of-day testing, and stimulant intake, that have been shown to have an influence on cognitive performance [56–58], thus having the potential to skew *non-decision time* and *drift rate* in our tasks, two of the DDM parameters analyzed in this study. Further studies should directly control such variables in order to exclude possible influences on the results obtained.

The tasks used were limited to three domains, financial decision making (delay discounting), emotion recognition and memory retrieval for words, so we cannot generalize the results to a broader spectrum of cognitive areas to pinpoint which are most impacted and how. Moreover, the nature of the *delay discounting* task and the two-stage procedure used to fit the DDM to this task prevent a direct comparison across tasks and results regarding this task should be interpreted with caution. However, our interest in testing predictions about changes in DDM parameters with aging across heterogeneous cognitive operations does not depend on the claim that these operations are psychologically equivalent. Indeed, the modest and inconsistent pairwise associations observed between *drift rates* across tasks suggest that these parameters may be interpreted as task-specific indices of information accumulation rather than as interchangeable measures of a unitary processing-speed construct. We interpret these findings within the context (1) that age-related slowing is observed across a heterogeneous range of cognitive operations (2) that the DDM has been productively used to model putative cognitive processes underlying a similarly heterogeneous range of cognitive operations, and (3) that investigators have begun to investigate whether age-related cognitive slowing can be mechanistically decomposed using the DDM.

In our analysis of white matter, we employed widely used scalar measures: FA and MD. Although these measures are broadly applied, they lack specificity and may indicate changes in various tissue properties—such as myelination degree, axonal density, and organization—without distinguishing among these mechanisms [59]. Fanning, bending and intersection of white matter tracts will make FA insensitive to microstructural changes within a particular voxel where such occurrences are present [60]. Furthermore, the interpretation of changes in FA as indicators of ‘integrity’ in white matter microstructure can be misleading, particularly when considering the complex multifactorial nature of diffusion signals [59]. This often leads to oversimplified or incorrect interpretations. Nevertheless, FA and MD are sensitive to alterations in tissue microstructure, irrespective of the underlying cause, providing valuable preliminary insights and paving the way for more detailed investigations [59]. An additional challenge arises from increased in-scanner head motion with age, which can affect FA and MD measures [61,62]. To address this, we utilized mean displacement values from the QUAD tool in FSL. Our results showed no significant association between mean displacement and age (S13 Fig). These findings might be specific to our cohort and thus we underscore the necessity of controlling for head motion in diffusion measures, particularly in studies on aging. Despite these complexities, the consistent relationships observed between age and both FA and MD across various studies support a robust age-dependent effect on these measures [63].

Recognizing the constraints of cross-sectional age-mediated analysis is also necessary. Mediation involves the sequential unfolding of causal processes over time and cross-sectional mediation models often yield significantly biased estimates of longitudinal parameters [64] and inconsistencies between longitudinal and cross-sectional results have been found in studies of cognitive aging [65]. For such analysis to be potentially justifiable it is essential that the temporal ordering of variables in the causal chain of mediation is clear.

Finally, even though we used age as a continuous variable to overcome the limitations of a simple group comparison and to be able to look at age-related associations in DDM parameters, particularly in the oldest age range, and even though our sample ranged from 53–95, the majority of these participants were above 65 years old and only 5 above 90 years old, which still limits our ability to characterize these associations in middle age and in later older adulthood. Moreover, our cohort was predominantly White and highly educated, which may limit the generalizability of our findings to more socioeconomically, educationally, and ethnically diverse aging populations.

## Conclusion

Our results suggest that there is a slowing of information processing with age, reflected in task-specific patterns of evidence accumulation across cognitive domains. An exploratory indirect association involving global FA, although not statistically significant after multiple-comparison correction, suggests that the potential role of age-related differences in white matter microstructure warrants further investigation. Larger studies, with an increased sample size and number of cognitive tasks are needed for a fuller understanding of the mechanisms of cognitive slowing with age, which might help tailor intervention strategies to specific mechanisms and could inform environmental modifications to promote older adults’ functional independence and quality of life.

## Supporting information

S1 Fig Histograms showing Reaction Time (RT) distributions pooled across participants for each task before (upper panel) and after (bottom panel) the exclusion of the 5% slowest RT for each participant. Histograms were generated using 0.5 second bins for the *delay discounting* task (left panel), 0.75 second bins for the *emotion recognition* task (middle panel) and 0.25 second bins for the *memory* task (right panel). (TIF)

S2 Fig Boxplots show the log-likelihood values for the simulated data sets (on the right) and empirical data sets (on the left) for each of the three experimental tasks. The lower boundary of the box indicates the 25% percentile, a black line within the box labels the median, and the upper boundary of the box indicates the 75% percentile of all values. Whiskers above and below the box indicate maximum and minimum values falling no more or no less than 1.5 times the length of the box. The horizontal line represents the 1% percentile of the fit values from the simulated data sets. Participants with values below this line were excluded from the analysis.
(TIF)

S3 Fig Plots show model fit based on the quantile-probability plots of empirical and predicted consistency/accuracy and RT distributions. From top to bottom scatterplots show predicted data (y-axis) plotted against empirical data (x-axis) for consistency/accuracy and for the 0.10, 0.25, 0.50, 0.75 and 0.90 quantiles of RT distribution for the *delay discounting*, *emotion recognition* and *memory* tasks (from left to right). Each point represents one participant. The diagonal line represents perfect model fit.
(TIF)

S4 Fig Plots show the parameter recovery analysis, with the empirical DDM parameter estimates and the recovered parameter estimates obtained after fitting the diffusion model to simulated datasets generated from the empirical parameter estimates. Results are shown for the *delay discounting* task (left panel), *emotion recognition* task (middle panel), and *memory* task (right panel) for *drift rate* (top row), *boundary separation* (middle row), and *non-decision time* (bottom row). Spearman correlation coefficients are shown for each comparison.
(TIF)

S5 Fig Boxplots show the values for mean *drift-rate*, *boundary separation*, *non-decision time* and RT for each of the three experimental tasks. The lower boundary of the box indicates the 25% percentile, a black line within the box labels the median, and the upper boundary of the box indicates the 75% percentile of all values. Whiskers above and below the box indicate maximum and minimum values falling no more or no less than 1.5 times the length of the box. Points falling outside above and below the upper and lower whiskers, respectively, represent possible outliers.
(TIF)

S6 Fig Boxplots show the values for mean *drift-rate*, *boundary separation* and *non-decision time* for each of the three experimental tasks, per age group. The lower boundary of the box indicates the 25% percentile, a black line within the box labels the median, a black diamond inside the box labels the mean, and the upper boundary of the box indicates the 75% percentile of all values. Whiskers above and below the box indicate maximum and minimum values falling no more or no less than 1.5 times the length of the box.
(TIF)

S7 Fig Plots for the pairwise associations between the *drift rates* across the three tasks. Spearman correlations were computed between *drift rate* estimates for each pair of tasks. Rho (95% Confidence Interval) is reported inside each plot with the sample size (N) on the top of each plot.
(TIF)

S8 Fig Plots for the association between age and *boundary separation* (top row) and age and *non-decision time* (bottom row) for the *delay discounting*, *emotion recognition* and *memory* tasks (from left to right). (TIF)

S9 Fig Histograms showing distributions of achieved task difficulty for the *emotion recognition* (left panel) and the *memory* (right panel) tasks. For the *memory* task, all participants reached the maximum word count; therefore, only final study time was used to represent individual differences in achieved difficulty. Lower values mean greater difficulty for both tasks.
(TIF)

S10 Fig Plots showing the estimates (rho) and 95% Confidence Intervals for the sensitivity analysis focusing on temporal mismatch between task performance and in-person neuropsychological testing for the elements included in the RT-based speed composite. Time windows are ≤1 year, ≤2 years, and (as originally preregistered) ≤3 years. No participants had in-person evaluations within 1 year of the *emotion recognition* or *memory* tasks.
(TIF)

S11 Fig Plots showing the bootstrapped unstandardized indirect effect and percentile bootstrapped Confidence Intervals for the sensitivity analysis focusing on temporal mismatch between task performance and neuroimaging measures. Time windows are ≤1 year, ≤2 years, ≤3 years, and (as originally preregistered) ≤4.
(TIF)

S12 Fig Whole brain voxel-wise analysis for the association between Mean Diffusivity (MD) and *non-decision time* in the *delay discounting* task. *Non-decision time* correlates positively with MD (p < 0.05, corrected, threshold-free cluster enhancement) in several white-matter tracts. The significant areas (orange) are superimposed on both the mean skeleton (green) and mean FA image of the full sample. Results are thickened for ease of visualization. All slices are illustrated in radiological (left-is-right) orientation.
(TIF)

S13 Fig Spearman correlation for the association between age and mean displacement (ρ = 0.043, p = 0.576). (TIF)

S1 Table Sensitivity analysis including all trials (without exclusion of the 5% slowest reaction times for each participant). Spearman correlations – rho (95% CI) [p-value] – between age and the drift diffusion model parameters and between age and mean RTs for each task. P-values for *drift rate* are Benjamini–Hochberg FDR-adjusted.
(DOCX)

S2 Table Sensitivity analysis including all trials (without exclusion of the 5% slowest reaction times for each participant). Spearman correlations – rho (95% Confidence Interval) [p-value] – between RT-based speed composite and the drift diffusion model parameters and between RT-based speed composite and mean RTs for each task. P-values for *drift rate* are Benjamini–Hochberg FDR-adjusted.
(DOCX)

S3 Table Spearman correlations – rho (95% Confidence Interval) [p-value] – between RT-based speed composite and the drift diffusion model parameters and between RT-based speed composite and mean RTs for each task, including only participants for whom a subsequent longitudinal evaluation was available following completion of the online tasks, at which they were confirmed to remain cognitively and neurologically healthy. P-values for *drift rate* are Benjamini–Hochberg FDR-adjusted.
(DOCX)
