# Supplementary material for: Age-related processes impacting cognitive processing speed across an older adult cohort: A drift diffusion model analysis
Source: PLoS One. 2026 Nov 13;21(11):e0361899. doi: 10.1371/journal.pone.0361899 (PMC13766253; doi:10.1371/journal.pone.0361899)
Supplement: Supplementary file 1 [file pone.0361899.s014.docx]

**Table A1.** Sensitivity analysis including all trials (without exclusion of the 5% slowest reaction times for each participant). Spearman correlations – rho (95% CI) [p-value] – between age and the drift diffusion model parameters and between age and mean RTs for each task. P-values for *drift rate* are Benjamini–Hochberg FDR-adjusted.

|  | **Drift rate** | **Boundary separation** | **Non-decision time** | **Reaction Time** |
| --- | --- | --- | --- | --- |
| **Delay discounting (N=162)** | -0.0035 (-0.16, 0.15) [0.96] | 0.12 (-0.033, 0.27) | 0.29 (0.15, 0.43) | 0.21 (0.053, 0.35) |
| **Emotion recognition (N=193)** | -0.17 (-0.3, -0.028) [0.038] | 0.12 (-0.021, 0.26) | 0.38 (0.25, 0.49) | 0.32 (0.19, 0.45) |
| **Memory (N=171)** | -0.25 (-0.39, -0.1) [0.0059] | 0.11 (-0.036, 0.26) | 0.39 (0.26, 0.51) | 0.38 (0.24, 0.5) |
