# Supplementary material for: Age-related processes impacting cognitive processing speed across an older adult cohort: A drift diffusion model analysis
Source: PLoS One. 2026 Nov 13;21(11):e0361899. doi: 10.1371/journal.pone.0361899 (PMC13766253; doi:10.1371/journal.pone.0361899)
Supplement: Supplementary file 2 [file pone.0361899.s015.docx]

**Table A2.** Sensitivity analysis including all trials (without exclusion of the 5% slowest reaction times for each participant). Spearman correlations – rho (95% Confidence Interval) [p-value] – between RT-based speed composite and the drift diffusion model parameters and between RT-based speed composite and mean RTs for each task. P-values for *drift rate* are Benjamini–Hochberg FDR-adjusted.

|  | **Drift rate** | **Boundary separation** | | **Non-decision time** | **Reaction time** |
| --- | --- | --- | --- | --- | --- |
| **Delay discounting**  **(N=157)** | -0.08 (-0.23, 0.077) [0.38] | 0.36 (0.21, 0.49) | | 0.18 (0.029, 0.33) | 0.4 (0.26, 0.52) |
|  |  |  |  | |  |
| **Emotion recognition**  **(N=106)** | -0.14 (-0.33, 0.048) [0.21] | 0.15 (-0.038, 0.33) | | 0.2 (0.0092, 0.38) | 0.31 (0.13, 0.47) |
|  |  |  | |  |  |
| **Memory**  **(N=95)** | -0.31 (-0.48, -0.11) [0.0078] | 0.31 (0.11, 0.48) | | 0.24 (0.043, 0.42) | 0.43 (0.25, 0.58) |
