# Supplementary material for: Age-related processes impacting cognitive processing speed across an older adult cohort: A drift diffusion model analysis
Source: PLoS One. 2026 Nov 13;21(11):e0361899. doi: 10.1371/journal.pone.0361899 (PMC13766253; doi:10.1371/journal.pone.0361899)
Supplement: Supplementary file 3 [file pone.0361899.s016.docx]

**Table A3.** Spearman correlations – rho (95% Confidence Interval) [p-value] – between RT-based speed composite and the drift diffusion model parameters and between RT-based speed composite and mean RTs for each task, including only participants for whom a subsequent longitudinal evaluation was available following completion of the online tasks, at which they were confirmed to remain cognitively and neurologically healthy. P-values for *drift rate* are Benjamini–Hochberg FDR-adjusted.

|  | **Drift rate** | **Boundary separation** | **Non-decision time** | **Reaction time** |
| --- | --- | --- | --- | --- |
| **Delay discounting (N=85)** | -0.17 (-0.37, 0.045) [.36] | 0.25 (0.041, 0.44) | 0.2 (-0.017, 0.39) | 0.33 (0.13, 0.51) |
| **Emotion recognition (N=54)** | 0.083 (-0.19, 0.34) [0.86] | -0.14 (-0.39, 0.13) | 0.24 (-0.025, 0.48) | 0.051 (-0.22, 0.31) |
| **Memory (N=49)** | -0.053 (-0.33, 0.23) [0.86] | 0.092 (-0.19, 0.36) | 0.0081 (-0.27, 0.29) | 0.17 (-0.12, 0.43) |
