# Supplementary material for: Age-related processes impacting cognitive processing speed across an older adult cohort: A drift diffusion model analysis
Source: PLoS One. 2026 Nov 13;21(11):e0361899. doi: 10.1371/journal.pone.0361899 (PMC13766253; doi:10.1371/journal.pone.0361899)
Supplement: Supplementary file 4 [file pone.0361899.s018.docx]

Dear Editor,

Thank you for giving us the opportunity of submitting a revised draft of our manuscript. We appreciate your time and the reviewers’ feedback. We have highlighted the changes within the manuscript to reflect the suggestions of the reviewers.

You will find below a point-by-point response to the reviewers’ specific comments and concerns. For reviewer 1 answers are given in the Specific Comments section given that each specific comment relates to a Major/Minor weakness, except for those that have no corresponding Specific Comments.

**Reviewer #1: General Comments**

Major Weaknesses:

1. Critical Temporal Confounding: The study methodology permits a time gap of up to three years between behavioral testing and neuropsychological assessment, and up to four years between behavioral testing and MRI acquisition. In a cohort of older adults ranging up to 95 years of age, white matter microstructure and cognitive processing speeds are subject to substantial nonlinear degeneration over a 48-month period. Correlating real-time decision-making parameters with historical neural architecture severely compromises the validity of the structure-function models.
2. Unjustified Omission of Statistical Corrections: The decision to forego multiple comparison corrections across three tasks and three distinct Drift Diffusion Model (DDM) parameters (resulting in numerous independent and non-independent tests) is statistically invalid. The primary structural finding—that global fractional anisotropy (FA) mediates age and drift rate in emotion recognition—relies on an uncorrected, marginal alpha ($p=0.044$). The authors explicitly acknowledge this finding would not survive correction, rendering the mechanistic conclusions fragile and highly susceptible to Type I error.
3. Methodological Heterogeneity in Response Coding: The delay discounting task boundary conditions are modeled dynamically based on a pre-estimated temporal preference parameter ($k$) to denote "consistent/inconsistent" choices. In contrast, the emotion recognition and memory tasks utilize objective "correct/error" boundaries. This fundamental difference in decision architecture confounds cross-task comparisons; a failure to accumulate evidence for a subjective preference is not psychologically equivalent to a failure to accumulate evidence for objective facial or lexical recognition.

We thank the reviewer for this point and have sought to clarify the relationship of this task to the central aim of the project. The drift diffusion model has been used by many investigators to model a wide and heterogenous range of cognitive operations, not limited to perceptual tasks (such as our *emotion recognition* task), but also including tasks requiring the internal maintenance and manipulation of representations (as in our *memory* task) and tasks of value-based decision-making (as in our *delay discounting* task). In addition, age-related slowing is observed across a similarly wide and heterogenous range of cognitive operations, including value-based decision-making; and several investigators have sought to mechanistically decompose such slowing in terms of changes in parameters of the DDM. Two contributions of our study to the literature are (1) modeling of age as a continuous parameter within a common cohort (rather than relying on group-level comparisons across young and old cohorts), and (2) estimation of DDM parameters across heterogeneous operations within the same cohort and with an overlapping set of demographic, neuropsychological, and neuroimaging measures. In the context of value-based decision-making, while these do not have an external or objective standard of “correctness” as in perceptual or memory decisions, one accepted approach is to analogize correct choices to those that maximize a subjective value/utility function (as within our task, “consistent” choices are those that maximize the subjective present value of a participant’s choice given their estimated *k* parameter; for related interpretations of accuracy or correctness in value-based decisions using the DDM see Milosavljevic et al, Judgment and Decision Making 2010, Copeland et al, Cogent Pyschology 2022.)

Then, to clarify the purposes of our study: our interest in testing predictions about changes in DDM parameters with aging across heterogeneous cognitive operations does not depend on the claim that these operations are psychologically equivalent. Instead, it stems from the recognition (1) that age-related slowing is observed across a heterogeneous range of cognitive operations, including value-based decision-making, (2) that the DDM has been productively used to model putative cognitive processes underlying s similarly heterogeneous range of cognitive operations, including value-based decision-making, and (3) that investigators have begun to investigate whether age-related cognitive slowing can be mechanistically decomposed using the DDM. In our view, the heterogeneity of the tasks included in our analysis is representative of the range of cognitive operations across which age-related cognitive slowing is observed and across which the DDM has been productively applied. In our preregistered exploratory analysis we evaluated whether changes in DDM parameters with aging across heterogeneous cognitive operations may reflect a common underlying mechanism (despite the psychological heterogeneity of these operations), although these analyses were generally inconclusive.

We have now made some changes to the manuscript:

- Page 2 lines 40-41: ‘*Age-related slowing is observed across a similarly wide and heterogenous range of cognitive operations.’*

- Page 12 lines 337-339: *‘In the context of value-based decision-making tasks, while these do not have an external or objective standard of “correctness” as in perceptual or memory decisions, a widely used approach is to analogize correct choices to those that maximize a subjective value/utility function.’*

*-* Page 24 lines 671-675: *‘Two contributions of our study to the literature are (1) modeling of age as a continuous parameter within a common cohort (rather than relying on group-level comparisons across young and old cohorts), and (2) estimation of DDM parameters across heterogeneous operations within the same cohort and with an overlapping set of demographic, neuropsychological, and neuroimaging measures.’*

*-* Page 26 lines 722-728: *‘However, our interest in testing predictions about changes in DDM parameters with aging across heterogeneous cognitive operations does not depend on the claim that these operations are psychologically equivalent. Instead, it stems from the recognition (1) that age-related slowing is observed across a heterogeneous range of cognitive operations (2) that the DDM has been productively used to model putative cognitive processes underlying a similarly heterogeneous range of cognitive operations, and (3) that investigators have begun to investigate whether age-related cognitive slowing can be mechanistically decomposed using the DDM.’*

4. Environmental Variance in Slow-RT Paradigms: Conducting unconstrained, home-based online data collection for millisecond-sensitive reaction time modeling introduces severe environmental noise. The resultant non-decision times ($t_0 > 2$ seconds) are abnormally long for DDM frameworks. While the authors cite precedent for slow-RT models, they fail to adequately control for the hardware, connectivity, and environmental distraction variances inherent to remote assessment in a geriatric cohort.

We thank the reviewer for this comment. We agree that unsupervised home-based testing may introduce additional sources of variability related to participants' environment, hardware, or temporary distractions. This was one of the motivations for excluding the slowest 5% of responses prior to DDM estimation, as these responses were considered more likely to reflect lapses of attention or environmental interruptions rather than the cognitive processes of interest. As requested by the reviewers, we have now included histograms of the reaction time distributions before and after trimming, together with a sensitivity analysis including all responses, which demonstrated that the main findings follow the same pattern.

Regarding the relatively long non-decision times, these values are expected given the characteristics of our experimental paradigms and are consistent with previous applications of the DDM to tasks with relatively slow response times in older adults. Non-decision time in this context reflects not only perceptual encoding and motor execution but also task-specific processes occurring outside evidence accumulation, and therefore should not be directly compared with values obtained from simple laboratory reaction-time paradigms.

Finally, we acknowledge that the home-based testing environment represents a limitation of the present study. We have expanded the Discussion accordingly.

Minor Weaknesses:

1. Cross-Sectional Mischaracterization: The manuscript frequently employs longitudinal terminology (e.g., "trajectory", "changes") despite relying entirely on cross-sectional data.
2. Homogeneous Cohort: The sample is demographically constrained, being 85-87% White and highly educated (mean >17 years), which limits the generalizability of the findings to broader aging populations.

We thank the reviewer for this observation. We agree that the demographic characteristics of our cohort, which is predominantly White and highly educated, may limit the generalizability of our findings to more diverse aging populations. We have now added this as a limitation to the Discussion:

- Page 27 lines 758-760: ‘*Moreover, our cohort was predominantly White and highly educated, which may limit the generalizability of our findings to more socioeconomically, educationally, and ethnically diverse aging populations.’*

Specific Comments

1. Page 8, Lines 26-27: The use of the term "trajectory" is methodologically inaccurate for a cross-sectional design. Replace with "cross-sectional age-related differences."

The term has been replaced throughout the manuscript:

- Page 1 line 21: *‘…was to evaluate the cross-sectional age-related differences of processes…’*

*- Page 4 line 103: ‘…cross-sectional age-related differences of drift rate…’*

*- Page 27 line 754: ‘…and to be able to look at age-related associations in DDM parameters…’*

*- Page 27 line 757: ‘…which still limits our ability to characterize these associations in middle age and in later older adulthood.’*

2. Page 13, Lines 158-164: The unconstrained, home-based online data collection introduces critical unmeasured chronobiological and environmental confounders. Variables such as sleep quality, time-of-day testing, and stimulant intake significantly alter cognitive processing speed and decision-making parameters, which are indistinguishable from true age-related cognitive decline in your dataset [Bouzouraa E, Dhahbi W, Ferchichi A, Geantă VA, Kunszabo MI, Chtourou H, Souissi N: Single-night sleep extension enhances morning physical and cognitive performance across time of day in physically active university students: a randomized crossover study. Life 2025, 15(8):1178] [Amor SB, Dhahbi W, Bougrine H, Bessifi M, Geantă VA, Ursu VE, Trabelsi K, Souissi N: Differential Time-of-Day Effects of Caffeine Capsule and Mouth Rinse on Cognitive Performance in Adolescent Male Volleyball Athletes: A Randomized Crossover Investigation. Life 2025, 16(1):33]. Furthermore, the lack of standardized assessment controls for these temporal artifacts undermines the integrity of the algorithmic diffusion models [Dhahbi W, Dergaa I: Machine learning with temporal control designs for testing rhythmic specificity in chronobiology: A multivariate framework proposal for distinguishing genuine biological rhythms from temporal artifacts. Chronobiology International 2026:1-8] [Dhahbi W, Chamari K: The Algorithmic Athlete: A Call to Standardize Assessment of Sensor Technologies and Artificial Intelligence. International Journal of Sports Physiology and Performance 2026, 1(aop):1-2]. You must add a limitations paragraph detailing these specific confounders and their potential to skew non-decision time and drift rate.

We thank the reviewer for raising this concern. The following lines have now been added to the limitations paragraph to address this concern:

- Page 26 lines 711-716: ‘*Another constraint inherent to our at-home testing design is the inability to control for variables such as sleep quality, time-of-day testing, and stimulant intake, that have been shown to have an influence on cognitive performance [56,57,58], thus having the potential to skew non-decision time and drift rate in our tasks, two of the DDM parameters analyzed in this study. Further studies should directly control such variables in order to exclude possible influences on the results obtained.’*

3. Page 13, Lines 170-174: The linkage of behavioral data to neuropsychological data acquired up to three years prior, and neuroimaging data up to four years prior, requires rigorous statistical justification. You must include a sensitivity analysis isolating participants with concurrent data ($<6$ months) to prove the extensive time gap does not drive the null or marginal associations.

We thank the reviewer for this important comment. We acknowledge that the interval between online task completion and the linked neuropsychological and neuroimaging assessments is a limitation of this study and reflects the longitudinal design of the cohort, with additional delays introduced by the COVID-19 pandemic. We note that this concern applies only to analyses involving the RT-based speed composite (hypothesis ii) and neuroimaging measures (exploratory hypothesis), as age and online task performance were assessed at the same time.

A sensitivity analysis restricted to assessments acquired within 6 months would retain only a small sample across tasks (N = 19 for the *delay discounting* task and N = 36 for the *emotion recognition* and *memory* tasks). Instead, we performed a sensitivity analysis restricted to participants for whom a subsequent longitudinal evaluation was available following completion of the online tasks, at which they were confirmed to remain cognitively and neurologically healthy. We added the following sentences to the Methods and Results, respectively:

- Page 14 lines 417-421: *‘.Given the time gap between online task evaluation and RT-based speed composite and neuroimaging acquisitions, sensitivity analysis for hypothesis ii and exploratory hypothesis iii was performed including only participants for whom a subsequent longitudinal evaluation was available following completion of the online tasks, at which they were confirmed to remain cognitively and neurologically healthy.’*

- Page 19 lines 528-533: *‘Table A3 shows results for the sensitivity analysis, including only participants for whom a subsequent longitudinal evaluation was available following completion of the online tasks, at which they were confirmed to remain cognitively and neurologically healthy. Given the reduced sample size, confidence intervals in this sensitivity analysis were wider and comparisons were not statistically significant, although the overall pattern of associations was generally consistent with the primary analyses.’*

- Page 20 lines 550-554: ‘*For the sensitivity analysis including only participants for whom a subsequent longitudinal evaluation was available following completion of the online tasks, at which they were confirmed to remain cognitively and neurologically healthy, the sample size was reduced to N=77 and bootstrapped unstandardized indirect effect was -3.3e-3 (p = 0.006).’*

4. Page 15, Lines 222-223: Excluding participants based solely on a mean displacement threshold of >1mm is excessively liberal for diffusion tensor imaging in aging populations, where sub-millimeter head motion introduces severe artifacts in FA and MD estimations. Re-evaluate the imaging data using framewise displacement (FD) thresholds or robust motion scrubbing protocols.

We thank the reviewer for raising this important point. We agree that head motion is an important potential confound in diffusion MRI studies, particularly in older adults.

However, our diffusion MRI data were processed using FSL's eddy, which jointly performs motion correction and eddy-current correction and incorporates slice-level detection and replacement of signal affected by motion during diffusion encoding. As described in the Methods, all MR images were visually inspected for motion-related artifacts (page 8 lines 218-219), all preprocessed images were visually inspected for registration errors, and FSL QUAD/SQUAD quality-control reports were reviewed prior to participant exclusion (page 8 lines 236-237). The FSL QUAD/SQUAD quality-control framework provides diffusion-specific motion metrics, and participants exceeding mean displacement > 1mm were excluded. In addition, mean displacement was not associated with age in the retained sample (Spearman's ρ = 0.043, p = 0.576) (as shown in Appendix Figure A9), reducing the likelihood that age-related associations were driven by systematic differences in head motion. We now report descriptive motion metrics for the retained sample:

- Page 8 lines 239-240: *‘Among the retained participants mean displacement was 0.53mm ± 0.37mm.’*

5. Page 16, Lines 259-262: Trimming the slowest 5% of responses and applying an arbitrary hard cutoff at 350ms alters the natural skew of the RT distribution, which is the mathematical foundation of DDM estimation. Provide distributional normality checks before and after this aggressive trimming.

We thank the reviewer for this comment. Regarding the 350 ms cutoff, this threshold was selected based on previous DDM studies in older adult cohorts. Furthermore, in our cohort no participant had reaction times faster than 350 ms; therefore, no trials were excluded by this criterion, as stated on page 15 lines 448–449.

Regarding the exclusion of the slowest 5% of responses, we agree that this trimming alters the right tail of the RT distribution. However, its purpose was to reduce the influence of lapses and environmental distractions, which are of particular concern given that the tasks were completed in an unconstrained home environment. We have now included histograms of the RT distributions before and after trimming (Appendix Figure A1). We added this sentence to the manuscript ‘*Visual inspection confirmed that the characteristic positively skewed RT distributions were preserved after the exclusion of the 5% slowest trials (Appendix Figure A1).*’ (page 15 lines 449-450).

Most importantly, we performed a sensitivity analysis including all responses (Appendix Tables A1 and A2). The results follow the same pattern, showing that our conclusions are not driven by the exclusion of the slowest 5% of responses.

6. Page 16, Lines 266-275: The rationale provided for omitting family-wise error correction is statistically inadequate. Non-independence of tasks does not justify evaluating an uncorrected threshold across multiple parameters. Apply False Discovery Rate (FDR) or Bonferroni adjustments to all correlational and mediation models.

We agree with the reviewer that non-independence among tasks does not, by itself, justify foregoing correction for multiple comparisons. Therefore, for the primary correlations, p-values were adjusted for multiple comparisons across 6 tests of the preregistered hypotheses (associations between *age* and *drift rate* for the three tasks, association between RT-based composite measure and *drift rate* for the three tasks) by applying Benjamini–Hochberg FDR correction. For ancillary analyses (involving *boundary separation* and *non-decision time*), effect sizes are now presented with 95% confidence intervals but without associated p-values to emphasize estimation rather than formal hypothesis testing. P-values adjusted for multiple comparisons across 10 tests (the 6 associations of the primary hypothesis and the 4 mediation models performed) are now reported for the mediation analysis.

Tables 3 and 4 have been updated to reflect these changes and the manuscript has been updated accordingly. The following changes were made to the manuscript:

- Page 5 lines 142-143: ‘*Preregistered ancillary analyses were also conducted to examine associations between age and boundary separation and non-decision time.’*

*-* Page 5 lines 145-146*: ‘Preregistered ancillary analyses were also conducted to examine associations between RT-based measures and boundary separation and non-decision time.’*

*-* Pages 11-12 lines 331-335: *‘Because our preregistered primary hypothesis was evaluated across three tasks, p-values for the drift rate analyses were adjusted for multiple comparisons using the Benjamini–Hochberg false discovery rate (FDR) procedure. Associations involving boundary separation and non-decision time were considered ancillary analyses and are reported as effect sizes with 95% confidence intervals.’*

*-*Page 14 lines 395-401*: ‘Because each primary hypothesis was evaluated across three tasks, p-values for 6 associations were adjusted for multiple comparisons using the Benjamini–Hochberg false discovery rate (FDR) procedure. Additionally, we examined the association between age and the other two diffusion model parameters (boundary-separation and non-decision time) and between the RT-based speed composite and these two parameters, as prespecified ancillary analyses. These ancillary analyses are reported as effect sizes with 95% confidence intervals.’*

*-* Page 14 lines 409-412*:* ‘*P-values for these exploratory analysis were adjusted for 10 comparisons (the 6 associations of the primary hypothesis and the 4 mediation models performed) using the Benjamini–Hochberg false discovery rate (FDR) procedure.’*

*-* Page 17 lines 497-505: *‘Age was significantly negatively associated with drift rate for both the emotion recognition (ρ = -0.19, p = 0.016) and the memory tasks (ρ = -0.18, p = 0.026) (Figure 1 and Table 3). Additionally, age was positively associated with boundary separation in the emotion recognition task (ρ = 0.16, 95% CI [0.017-0.29]), whereas little evidence of an association was observed in the other tasks. Age was also positively associated with non-decision time across all three tasks (delay discounting: ρ = 0.33, 95% CI [0.19-0.46]; emotion recognition: ρ = 0.37, 95% CI [0.24-0.49]; memory: ρ = 0.39, 95% CI [0.25-0.51]) (Table 3 and Appendix Figure A7).’*

- Pages 18-19 lines 519-526: *‘The RT-based speed composite was significantly negatively associated with drift rate for both the delay discounting (ρ = -0.274, p = 0.003) and the memory tasks (ρ = -0.271, p = 0.016). In addition, a positive association was found between the RT-based speed composite and boundary separation for the delay discounting (ρ =0.272, 95% CI [0.12-0.41]) and the memory tasks (ρ = 0.223, 95% CI [0.022-0.41]), and the association between the RT-based speed composite and non-decision time was positive across all three tasks (delay discounting: ρ = 0.19, 95% CI [0.03-0.33]; emotion recognition: ρ = 0.33, 95% CI [0.15-0.49]; memory: ρ = 0.25, 95% CI [0.047-0.43]) (Table 4).’*

*-* Pages 19-20 lines 546-548*: ‘The bootstrapped unstandardized indirect effect was -1.69e-3 (uncorrected p = 0.036). However, this result did not persist when adjusted for multiple comparisons (p = 0.067).’*

Please note that the original uncorrected indirect effect and p-value for the mediation analysis have been corrected from -1.23e-3 to -1.69e-3 and 0.044 to 0.036, respectively. This change reflects a transcription error identified during the revision process.

7. Page 17, Lines 283-290: Extracting the discounting parameter $k$ via maximum likelihood prior to executing the DDM introduces mathematical dependency. You are fitting a model onto the residuals of another model. You must justify why a hierarchical Bayesian DDM combining the hyperbolic discount function directly into the drift rate estimation was not utilized.

We agree that the delay-discounting DDM was conditional on the first-stage estimate of *k*. We have clarified this in the manuscript:

- Page 23 lines 618-621: ‘*Finally, we used a two-stage procedure for this task, applying maximum likelihood estimation to the hyperbolic discounting model to estimate k – for posterior response coding – before fitting the DDM, making the delay discounting DDM conditional on this first stage.’*

We now also acknowledge it as a limitation:

- Page 26 lines 719-722: *‘Moreover, the nature of the delay discounting task and the two-stage procedure used to fit the DDM to this task prevent an optimal comparison across tasks and results regarding this task should be interpreted with caution.’*

However, *k* was used only to define participant-specific response coding in a task without objectively correct responses, not as a regressor or residualized predictor in the DDM. The purpose was to create response categories analogous to correct/incorrect classifications in the other tasks while maintaining a common fast-dm framework across tasks.

We agree that a joint hierarchical Bayesian model incorporating the hyperbolic value function directly into trialwise drift rate might be a more powerful approach. However, this would require a different modeling framework from fast-dm and would reduce comparability with the other two tasks. We now present our approach as a simpler two-stage approximation and note that future work should use joint estimation:

- Page 23 lines 621-623: *‘Future work using joint modeling approaches such as hierarchical Bayesian DDMs might lead to more robust conclusions [49,50].’*

8. Page 20, Lines 371-375: The final trial counts (87 for delay discounting) are perilously low for reliable DDM parameter estimation, particularly when inter-trial variability parameters are included. Report the parameter recovery and test-retest reliability metrics for this specific trial density.

While we agree that 87 trials might be low for stable estimation of inter-trial variability parameters, in our model, *inter-trial variability of non-decision time*$\text{ }$was included to improve characterization of the RT distribution (page 12, lines 357-358) , but its estimates were not used in any between-subject analyses or interpreted substantively. We therefore focused parameter recovery analyses on the parameters used for our analyses: *drift rate, boundary separation and non-decision time.* The following sentences have been added to the manuscript:

- Page 13 lines 386-389: ‘*Lastly, parameter recovery was assessed by comparing the empirical DDM parameter estimates (drift rate, boundary separation and non-decision time) with those re-estimated after fitting the model to simulated datasets generated from the original parameter estimates, using Spearman correlations*.’

- Page 16 lines 478-481: *‘Parameter recovery analyses further demonstrated good recovery of the estimated DDM parameters across all three tasks, with strong correlations between the original and re-estimated values for drift rate (ρ = 0.84–0.95), boundary separation (ρ = 0.93–0.98), and non-decision time (ρ = 0.96–0.97) (all p < 0.001) (Appendix Figure A4).’*

Parameter recovery results are now shown in Appendix Figure A4.

Regarding test-retest reliability, we agree that this is an important property of DDM parameter estimation. However, evaluation of this property typically requires repeated administration of the same task to the same participants. Because the present study included a single administration of each task, we were not able to evaluate this property with the available data.

9. Page 23, Lines 450-451: The unstandardized indirect effect of global FA (-1.23e-3, $p=0.044$) is marginally significant. Given the text acknowledges this fails multiple comparison correction, this specific finding must be downgraded in the abstract and discussion from a definitive mechanism to a strictly exploratory, underpowered observation.

We thank the reviewer for this comment. We have now rephrased the sentence in the Abstract:

- Page 1 lines 24-26: *‘…and we show that this association in the emotion recognition task might be partially accounted for by global fractional anisotropy.’*

We also rephrased the presentation of this result in the Discussion:

- Page 21 lines 580-581: *‘…our mediation models suggest that white matter microstructure changes have a significant indirect effect in the association between age and drift rate in the emotion recognition task*.’

- Page 24 lines 661-662: ‘…*variables suggests that global FA might partially account for the association between age and drift rate in the emotion recognition task.’*

Finally, we added the following sentence to the Discussion:

- Page 24 lines 666-669: *‘However, given the marginally significant result we report, that did not survive multiple comparisons correction, this result should be interpreted with caution and be seen as exploratory rather than definite, needing further research to reach more precise conclusions.*’

10. Page 26, Lines 537-539: The assertion that this study effectively evaluates the "oldest old" is overstated. With group sizes of $N=19$, $N=31$, and $N=29$ for participants >85 years old across the three tasks, the study lacks the statistical power to reliably model DDM variance in this specific age stratum.

We agree that, although our cohort spans a broad age range (53–95 years), the number of participants aged over 85 years is relatively modest, limiting our ability to characterize DDM variability specifically within the oldest-old age group. We acknowledge this limitation in the final paragraph of the Discussion (page 27, lines 753–757). Our goal was not to suggest that the present study was specifically designed to model age-related effects within the oldest-old subgroup. Rather, we aimed to highlight that our cohort extends into this age range, which has been underrepresented in previous studies using a continuous measure of age.

11. Page 28, Lines 616-619: The discussion of cross-sectional mediation limitations is placed too late in the manuscript and downplays the severity of the mathematical bias. Causal chain language (e.g., "impact", "underlying") must be systematically removed from the entire manuscript.

We thank the reviewer for this comment. The discussion of the limitations of the mediation analysis was placed alongside the presentation of the mediation results, as these analyses are presented last in the manuscript. Our intention was to discuss the limitations in the context of the corresponding findings, particularly as the mediation analyses were exploratory and not a primary focus of the study. Nevertheless, we agree with the reviewer's concern regarding the interpretation of mediation analyses in cross-sectional data. We have now changed causal chain language throughout the manuscript:

- Page 3 lines 85-88: *‘…and found that the association between age and drift rate showed a significant indirect effect through white matter measures within the frontoparietal network, but the association between age and the non-decision time parameter did not show such effects.’*

- Page 5 line 147: *‘…parameter this association is partially accounted for by changes in white matter microstructure.’*

- Page 19 line 544: *‘Global FA showed a significant indirect effect in the association between age and drift rate…’*

- Page 20 lines 548-550: *‘…nor global MD showed significant indirect effects in the associations between age and drift rate nor in the associations between age and boundary separation and non-decision time…’*

- Page 21 lines 580-581: *‘…that white matter microstructure changes have a significant indirect effect in the association between age and drift rate in the emotion recognition task.’*

- Page 24 lines 661-662: *‘…suggests that global FA might partially account for the association between age and drift rate…’*

**Reviewer #2:**

The manuscript addresses an important and timely question regarding the mechanisms underlying age-related slowing in cognition by applying drift diffusion modelling (DDM) across a relatively large cohort of older adults spanning middle age to the oldest-old. The study has several notable strengths, including preregistration, inclusion of participants over 85 years of age, explicit model-fit evaluation, sensitivity analyses, and the attempt to combine computational modelling with white matter measures. The use of age as a continuous variable rather than relying solely on young-versus-old group comparisons is also commendable and represents an important contribution to the aging literature.

At the same time, the manuscript raises several conceptual and methodological concerns that limit the strength of the conclusions currently drawn. In particular, the interpretation of drift rate as a relatively unitary measure of “cognitive processing speed” across highly heterogeneous tasks appears overly strong. The manuscript would benefit substantially from a more cautious and theoretically constrained interpretation of the DDM parameters, clearer justification of task selection, more detailed task descriptions, and a more rigorous treatment of several statistical and methodological issues.

Overall, I believe the study has the potential to make a valuable contribution, but the manuscript would benefit from substantial revision before the conclusions can be considered fully supported.

Major Issues

**1.** Overgeneralized interpretation of drift rate across fundamentally different tasks

A central concern is the interpretation of drift rate as a relatively common measure of “cognitive processing speed” across the three experimental paradigms. While drift rate may often reflect evidence accumulation efficiency, its psychological meaning is highly task-dependent.

The three tasks used in this study appear to involve substantially different latent cognitive processes:

• delay discounting: value-based preference decisions / decision conflict / impulsivity,

• emotion recognition: perceptual-social discrimination,

• memory task: episodic retrieval / familiarity-based recognition.

As a result, it is unclear whether drift rate estimates across these tasks reflect the same underlying construct. This concern is particularly strong for the delay discounting paradigm, where responses are coded as “consistent” or “inconsistent” with each participant’s estimated preference function rather than objectively correct or incorrect. In this context, drift rate may reflect preference consistency or decision certainty rather than cognitive processing speed per se.

The manuscript would benefit from a clearer theoretical framework explaining:

• why these three tasks were selected,

• what common mechanism is hypothesized across them,

• and whether the authors truly intend drift rate to be interpreted comparably across all paradigms.

At present, the manuscript sometimes reads as though all three tasks are interchangeable “processing speed tasks,” which is difficult to justify conceptually.

We thank the reviewer for this point and have sought to clarify the relationship of this task to the central aim of the project. The drift diffusion model has been used by many investigators to model a wide and heterogenous range of cognitive operations, not limited to perceptual tasks (such as our *emotion recognition* task), but also including tasks requiring the internal maintenance and manipulation of representations (as in our *memory* task) and tasks of value-based decision-making (as in our *delay discounting* task). In addition, age-related slowing is observed across a similarly wide and heterogenous range of cognitive operations, including value-based decision-making; and several investigators have sought to mechanistically decompose such slowing in terms of changes in parameters of the DDM. Two contributions of our study to the literature are (1) modeling of age as a continuous parameter within a common cohort (rather than relying on group-level comparisons across young and old cohorts), and (2) estimation of DDM parameters across heterogeneous operations within the same cohort and with an overlapping set of demographic, neuropsychological, and neuroimaging measures. In the context of value-based decision-making, while these do not have an external or objective standard of “correctness” as in perceptual or memory decisions, a widely-used approach is to analogize correct choices to those that maximize a subjective value/utility function (as within our task, “consistent” choices are those that maximize the subjective present value of a participant’s choice given their estimated *k* parameter; for related interpretations of accuracy or correctness in value-based decisions using the DDM see Milosavljevic et al, Judgment and Decision Making 2010, Copeland et al, Cogent Pyschology 2022.)

Then, to clarify the purposes of our study: our interest in testing predictions about changes in DDM parameters with aging across heterogeneous cognitive operations does not depend on the claim that these operations are psychologically equivalent. Instead, it stems from the recognition (1) that age-related slowing is observed across a heterogeneous range of cognitive operations, including value-based decision-making, (2) that the DDM has been productively used to model putative cognitive processes underlying s similarly heterogeneous range of cognitive operations, including value-based decision-making, and (3) that investigators have begun to investigate whether age-related cognitive slowing can be mechanistically decomposed using the DDM. In our view, the heterogeneity of the tasks included in our analysis is representative of the range of cognitive operations across which age-related cognitive slowing is observed and across which the DDM has been productively applied. In our preregistered exploratory analysis we evaluated whether changes in DDM parameters with aging across heterogeneous cognitive operations may reflect a common underlying mechanism (despite the psychological heterogeneity of these operations), although these analyses were generally inconclusive.

We have now made some changes to the manuscript:

- Page 2 lines 40-41: ‘*Age-related slowing is observed across a similarly wide and heterogenous range of cognitive operations.’*

- Page 12 lines 337-339: *‘In the context of value-based decision-making tasks, while these do not have an external or objective standard of “correctness” as in perceptual or memory decisions, a widely used approach is to analogize correct choices to those that maximize a subjective value/utility function.’*

*-* Page 24 lines 671-675: *‘Two contributions of our study to the literature are (1) modeling of age as a continuous parameter within a common cohort (rather than relying on group-level comparisons across young and old cohorts), and (2) estimation of DDM parameters across heterogeneous operations within the same cohort and with an overlapping set of demographic, neuropsychological, and neuroimaging measures.’*

*-* Page 26 lines 722-728: *‘However, our interest in testing predictions about changes in DDM parameters with aging across heterogeneous cognitive operations does not depend on the claim that these operations are psychologically equivalent. Instead, it stems from the recognition (1) that age-related slowing is observed across a heterogeneous range of cognitive operations (2) that the DDM has been productively used to model putative cognitive processes underlying a similarly heterogeneous range of cognitive operations, and (3) that investigators have begun to investigate whether age-related cognitive slowing can be mechanistically decomposed using the DDM.’*

**2.** Claims regarding “task specificity” are stronger than warranted by the data

The manuscript repeatedly suggests that age-related slowing in drift rate is task-specific because significant associations were found in the emotion recognition and memory tasks but not in the delay discounting task.

However, the nonsignificant association in the delay discounting task was reportedly in the same direction as the significant effects in the other tasks. The manuscript correctly acknowledges that “significant versus nonsignificant” does not demonstrate a true difference between effects, but this important caveat is not sufficiently integrated into the overall interpretation.

No formal statistical comparison between correlations/effects across tasks appears to have been performed. Therefore, the data do not yet demonstrate genuine task specificity.

The conclusions should therefore be substantially softened, or direct statistical comparisons between task effects should be added.

We agree that the presence of significant associations in some tasks and a nonsignificant association in another task does not, by itself, demonstrate a statistically reliable difference between tasks. Accordingly, we have revised the manuscript to avoid strong claims of task specificity and now describe the findings more cautiously. The following changes were made:

- Page 1 lines 26-28: ‘*Our results suggest a slowing of information processing with age that may be more evident in some cognitive domains than others, although formal comparisons between tasks were not conducted.’*

- Page 21 lines 585-586: *‘… suggesting that declines in drift rate with age (reflecting a slowing of information accumulation) may be more apparent in some cognitive domains than others.’*

- Page 27 lines 762-763: *‘…that the strength of this association may vary across cognitive domains*…’

**3.** Insufficient detail and reproducibility of task descriptions

The experimental task descriptions lack sufficient methodological detail for replication and, more importantly, make it difficult to interpret the DDM parameters psychologically.

Several critical details are missing or insufficiently specified, including:

• exact trial timing,

• fixation/intertrial intervals,

• response windows,

• instructions,

• practice procedures,

• stimulus balancing/randomization,

• staircase parameters,

• and difficulty progression rules.

This issue is particularly important because DDM parameter interpretation depends heavily on task architecture.

Delay discounting task

The description raises conceptual concerns regarding what participants were actually required to compute during the task. If participants rapidly adopt a stable strategy (e.g., always selecting delayed larger rewards), then the task may become more reflective of preference policy execution than ongoing evidence accumulation.

Emotion recognition task

The adaptive staircase procedure is insufficiently described. Important information is missing regarding:

• starting difficulty,

• step sizes,

• reversal criteria,

• separate staircases by emotion,

• and stopping rules.

More importantly, because task difficulty was adaptively adjusted per participant, it becomes unclear how directly comparable drift rate estimates are across individuals. Drift rate is strongly influenced by stimulus discriminability/task difficulty, and participants may effectively have completed different tasks.

Memory task

The memory task adaptively manipulated:

• encoding duration,

• retention interval,

• and memory load simultaneously.

This creates major interpretational ambiguity regarding which aspect of task difficulty contributed to drift rate differences. It is also unclear how these varying task parameters were accounted for in the analyses.

The manuscript should more explicitly discuss how adaptive task procedures influence between-subject interpretation of DDM parameters.

We thank the reviewer for raising this concern. We agree that adaptive task procedures deserve careful consideration when interpreting drift rate estimates. The adaptive procedures implemented in the *emotion recognition* and *memory* tasks were specifically designed to maintain participant performance within a comparable accuracy range (approximately 66–70%), thereby reducing floor and ceiling effects and allowing participants with different abilities to perform the tasks at a comparable level of difficulty. Consequently, although individual participants were presented with different stimulus difficulties, the adaptive procedures produced similar overall task difficulty rather than created fundamentally different tasks. We have now added more details regarding each task structure.

Regarding the *delay discounting* task:

- Page 9 lines 244-247: ‘*Within trials, three parameters were varied independently: percent penalty (the relative difference in magnitudes between the smaller immediate and larger delayed reward), delay length (time required to wait for the delayed reward), and absolute reward magnitudes (expressed in terms of the delayed reward magnitude).’*

*-* Page 9 lines 250-253: ‘*As a control condition to ensure task comprehension, 12 trials were interspersed throughout the task either asking participants to correctly indicate which reward would be paid out sooner, or asking participants to correctly indicate which reward was the larger reward.’*

*-* Page 9 lines 254-263: *‘Trial order was fixed for every participant, there was no constraint of time to answer and no fixation between trials (each trial started as soon as the participant answered the previous trial). Instructions to the participants were as follows: ‘We are interested in how people make decisions about money now and in the future. As a token of our appreciation, you will receive an Amazon gift card worth at least $5. We will ask you 104 questions about rewards that you could receive, either today or in the future. There are three types of questions... 'Which reward is paid SOONER?' 'Which reward is LARGER?' 'Which reward do you WANT?' Most of the questions will be about which reward you WANT. At the end, we will randomly choose one of these questions to count for real money. You will receive the reward (either today or in the future) that you chose.’*

Regarding the *emotion recognition* task:

-Page 10 lines 272-286: *‘…reversal occurred after 1 incorrect response or 2 correct responses in a row. Starting difficulty was fixed at 30 on a scale of 5-95 and the step size was fixed at 5, with no separate staircases by emotion. Stopping was not based on reversal counts but once 160 trials had been reached. Emotions were superimposed on 10 unique faces and the number of trials for each emotion was 28 for anger, 26 for happiness, 28 for surprise, 24 for disgust, 25 for sadness and 29 for fear. The emotion shown on each trial was randomized, there was no constraint of time to answer and no fixation between trials (each trial started as soon as the participant answered the previous trial). Instructions to the participants were as follows: ‘This is a study about recognizing emotions. As a token of our appreciation, you will receive a $15 Amazon gift card. This task has multiple trials, and each trial will begin with a choice like this: You'll click on which emotion you think the model is expressing. You will answer these questions about 160 faces with different expressions. Some of the emotions may be easy for you to identify, and some may be hard for you to identify. Please be sure to try your best throughout the experiment! Let's try a few practice rounds before we begin. Click the button below when you are ready to start.’’*

Regarding the *memory* task:

- Page 10 lines 293-294*: ‘Study slide and wait slide were always 30 seconds combined.’*

*-* Pages 10-11 lines 299-320*: ‘Regarding study time, for more than 4 correct responses , there was no change in study time. For 5 or 6 correct responses, study time decreased by 4 seconds. For less than 4 correct responses, study time increased by 4 seconds. If study time dropped below 12 seconds, then word count increased by one level and study time was reset to 16 seconds. If study time went above 20 seconds, word count went down one level and study time was reset to 16 seconds. In cases where a participant had all 6 correct responses on a current trial and an overall accuracy of > 70% for all trials or, inversely, less than 3 correct responses on current trial and an overall accuracy of < 60%, the step value was doubled. Participants answered a total of 144 2-alternative forced-choice questions. There was no constraint of time to answer and no fixation between trials (each trial started as soon as the participant answered the previous trial). Instructions to the participants were as follows: ‘This is a study about memory. As a token of our appreciation, you will receive a $15 Amazon gift card. Please scroll through to the bottom of this page to view the structure of this task. Once you reach the bottom of the page, you will find a button to proceed to the next page. This task has multiple trials. For each trial, you will receive an allotted amount of time to memorize the list of words displayed. For example: Next, you'll wait for an amount of time before answering a series of questions about the list of words. Once the waiting time has concluded, we will test your memory for some of the words you have just memorized. We will show you two words at a time, one of which was on the list you just memorized and one that was not. You will answer questions about 24 different lists of words. Some of the lists may be easy to remember, and some may be hard to remember. Please be sure to try your best throughout the experiment! Let's try a few practice rounds before we begin. Click the button below when you are ready to start.’’*

We have also added a paragraph to the Discussion clarifying that drift rate estimates should be interpreted as reflecting the efficiency of evidence accumulation under task conditions individually adjusted to achieve comparable levels of performance, while acknowledging that adaptive procedures may nevertheless limit the direct comparability of drift rate estimates across individuals.

- Page 23 lines 624-631: ‘*The adaptive procedures used in the emotion recognition and memory tasks also deserve consideration when interpreting drift rate. These procedures were implemented to maintain participant performance within a comparable accuracy range (approximately 66–70%), thereby reducing floor and ceiling effects and maintaining a similar level of task difficulty across individuals with different abilities. Consequently, although participants were not presented with identical stimulus difficulties, drift rate should be interpreted as reflecting the efficiency of evidence accumulation under task conditions individually adjusted to achieve comparable levels of performance.’*

**4.** Temporal mismatch between behavioural, neuropsychological, and MRI measures

The study allows up to:

• 3 years between behavioural and neuropsychological measures,

• and 4 years between behavioural and MRI acquisition.

Given the advanced age range of the cohort (including participants over 85 years old), this is potentially problematic. White matter integrity, processing speed, and cognitive performance can change substantially over these intervals in older populations.

Although the TBSS analyses included time difference covariates, it is unclear whether the mediation analyses adequately accounted for these temporal discrepancies.

This issue substantially weakens mechanistic interpretations linking white matter changes to DDM parameters.

We thank the reviewer for this important comment. We acknowledge that the interval between online task completion and the linked neuropsychological and neuroimaging assessments is a limitation of this study and reflects the longitudinal design of the cohort, with additional delays introduced by the COVID-19 pandemic. We note that this concern applies only to analyses involving the RT-based speed composite (hypothesis ii) and neuroimaging measures (exploratory hypothesis), as age and online task performance were assessed at the same time.

We now performed a sensitivity analysis restricted to participants who remained cognitively and neurologically healthy at the first clinical evaluation following completion of the online tasks. We also clarify that time difference covariates were also included in the mediation analysis. We added the following sentences to the Methods and Results, respectively:

*-* Page 14 lines 405-406: *‘The difference (in days) between DTI acquisitions and task performance was used as a covariate.’*

- Page 14 lines 417-421: *‘.Given the time gap between online task evaluation and RT-based speed composite and neuroimaging acquisitions, sensitivity analysis for hypothesis ii and exploratory hypothesis iii was performed including only participants for whom a subsequent longitudinal evaluation was available following completion of the online tasks, at which they were confirmed to remain cognitively and neurologically healthy.’*

- Page 19 lines 528-533: *‘Table A3 shows results for the sensitivity analysis, including only participants for whom a subsequent longitudinal evaluation was available following completion of the online tasks, at which they were confirmed to remain cognitively and neurologically healthy. Given the reduced sample size, confidence intervals in this sensitivity analysis were wider and comparisons were not statistically significant, although the overall pattern of associations was generally consistent with the primary analyses.’*

- Page 17 lines 478-481: ‘*For the sensitivity analysis including only participants for whom a subsequent longitudinal evaluation was available following completion of the online tasks, at which they were confirmed to remain cognitively and neurologically healthy, the sample size was reduced to N=77 and bootstrapped unstandardized indirect effect was -3.3e-3 (p = 0.006).’*

**5.** Mediation analyses appear overinterpreted

The mediation findings should be interpreted more cautiously.

The significant mediation effect:

• was modest,

• would not survive correction for multiple comparisons according to the authors themselves,

• was based on cross-sectional data,

• and involved temporally mismatched measures.

Furthermore, the mediation used global FA averages, which substantially reduces anatomical specificity. Meanwhile, the voxelwise TBSS analyses did not show strong converging associations between regional white matter measures and drift rate parameters.

As a result, the manuscript should avoid mechanistic or causal interpretations suggesting that white matter alterations explain age-related slowing in evidence accumulation.

We understand the reviewers’ concern regarding the interpretation of the mediation results. We have now rephrased the sentence in the Abstract:

- Page 1 lines 24-26: ‘…*and we show that this association in the emotion recognition task might be partially accounted for by global fractional anisotropy.’*

We also rephrased the presentation of this result in the Discussion

- Page 24 lines 661-662: *‘…variables suggests that global FA might partially account for the association between age and drift rate in the emotion recognition task*’.

And we added this sentence to the Discussion:

- Page 24 lines 666-669: ‘*However, given the marginally significant result we report, that did not survive multiple comparisons correction, this result should be interpreted with caution and be seen as exploratory rather than definite, needing further research to reach more precise conclusions*.’

We have now also changed causal chain language throughout the manuscript:

- Page 3 lines 85-88: *‘…and found that the association between age and drift rate showed a significant indirect effect through white matter measures within the frontoparietal network, but the association between age and the non-decision time parameter did not show such effects.’*

- Page 5 line 147: *‘…parameter this association is partially accounted for by changes in white matter microstructure.’*

- Page 19 line 544: *‘Global FA showed a significant indirect effect in the association between age and drift rate…’*

- Page 20 lines 548-550: *‘…nor global MD showed significant indirect effects in the associations between age and drift rate nor in the associations between age and boundary separation and non-decision time…’*

- Page 21 lines 580-581: *‘…that white matter microstructure changes have a significant indirect effect in the association between age and drift rate in the emotion recognition task.’*

- Page 24 lines 661-662: *‘…suggests that global FA might partially account for the association between age and drift rate…’*

**6.** Statistical analyses are under-modelled relative to the complexity of the questions

The main behavioural analyses rely primarily on simple bivariate Spearman correlations.

Given:

• the broad age range,

• unequal age-group sizes,

• highly educated sample,

• online testing environment,

• adaptive task difficulty,

• and temporally mismatched multimodal data,

this analytic strategy appears overly simplistic.

Particularly concerning is the complete absence of nonlinear analyses. The manuscript repeatedly discusses aging trajectories and lifespan changes, yet all analyses appear effectively monotonic/linear despite the high likelihood of nonlinear aging effects in oldest-old populations.

The manuscript would benefit from:

• regression-based analyses,

• covariate adjustment,

• nonlinear modelling,

• or at minimum stronger justification for the analytic simplicity.

We thank the reviewer for this comment. Our main goal was to examine the associations between age and DDM parameters. Accordingly, our preregistered analyses used Spearman's rank correlations, a non-parametric approach chosen a priori because it assesses monotonic associations without assuming linear relationships.

We agree that nonlinear age effects exist and have been reported previously (e.g., Krause et al., 2022). However, in that study, *drift rate* was relatively stable until approximately 60 years of age, after which the estimated decline was mainly monotonic decline. Given that our cohort included participants aged 53–95 years, with the majority of participants over 65 years of age, we considered Spearman's rank correlation an appropriate approach to quantify monotonic age-related associations within the age range represented in our sample. We also note that our primary analyses were preregistered, and our objective was not to model age-related effects but rather to test the prespecified hypotheses regarding the association between age and DDM parameters. We added this sentence to the methods to clarify this:

-Page 14 lines 394-395: *‘Spearman's correlation was selected a priori as a non-parametric approach to assess monotonic associations without assuming linear relationships.’*

Finally, we have revised the manuscript to avoid language implying that age trajectories were modelled:

- Page 1 line 21: *‘…was to evaluate the cross-sectional age-related differences of processes…’*

*- Page 4 line 103: ‘…cross-sectional age-related differences of drift rate…’*

- Page 27 line 754: *‘…and to be able to look at age-related associations in DDM parameters…’*

*- Page 27 line 757: ‘…which still limits our ability to characterize these associations in middle age and in later older adulthood.’*

**7.** Multiple comparison concerns remain substantial

The manuscript explicitly states that no multiple-comparison correction was applied.

Although the preregistration and primary/ancillary distinction are appreciated, the study nevertheless includes a large number of:

• correlations,

• DDM parameters,

• tasks,

• mediation analyses,

• and exploratory neuroimaging analyses.

This issue is especially relevant given that some key findings appear statistically fragile.

We thank the reviewer for raising this concern. We have now updated our statistics. For the primary correlations, p-values were adjusted for multiple comparisons across 6 tests of the preregistered hypotheses (associations between *age* and *drift rate* for the three tasks, association between RT-based composite measure and *drift rate* for the three tasks) and by applying Benjamini–Hochberg FDR correction. For ancillary analyses (involving *boundary separation* and *non-decision time*), effect sizes are now presented with 95% confidence intervals but without associated p-values to emphasize estimation rather than formal hypothesis testing. P-values adjusted for multiple comparisons across 10 tests (the 6 associations of the primary hypothesis and the 4 mediation models performed) are now reported for the mediation analysis.

Tables 3 and 4 have been updated to reflect these changes and the manuscript has been updated accordingly. The following changes were made to the manuscript:
- Page 5 lines 142-143: ‘*Preregistered ancillary analyses were also conducted to examine associations between age and boundary separation and non-decision time.’*

*-* Page 5 lines 145-146*: ‘Preregistered ancillary analyses were also conducted to examine associations between RT-based measures and boundary separation and non-decision time.’*

*-* Pages 11-12 lines 331-335: *‘Because our preregistered primary hypothesis was evaluated across three tasks, p-values for the drift rate analyses were adjusted for multiple comparisons using the Benjamini–Hochberg false discovery rate (FDR) procedure. Associations involving boundary separation and non-decision time were considered ancillary analyses and are reported as effect sizes with 95% confidence intervals.’*

*-*Page 14 lines 395-401*: ‘Because each primary hypothesis was evaluated across three tasks, p-values for 6 associations were adjusted for multiple comparisons using the Benjamini–Hochberg false discovery rate (FDR) procedure. Additionally, we examined the association between age and the other two diffusion model parameters (boundary-separation and non-decision time) and between the RT-based speed composite and these two parameters, as prespecified ancillary analyses. These ancillary analyses are reported as effect sizes with 95% confidence intervals.’*

*- Page 14 lines 409-412: ‘P-values for these exploratory analysis were adjusted for 10 comparisons (the 6 associations of the primary hypothesis and the 4 mediation models performed) using the Benjamini–Hochberg false discovery rate (FDR) procedure.’*

*-* Page 17 lines 497-505: *‘Age was significantly negatively associated with drift rate for both the emotion recognition (ρ = -0.19, p = 0.016) and the memory tasks (ρ = -0.18, p = 0.026) (Figure 1 and Table 3). Additionally, age was positively associated with boundary separation in the emotion recognition task (ρ = 0.16, 95% CI [0.017-0.29]), whereas little evidence of an association was observed in the other tasks. Age was also positively associated with non-decision time across all three tasks (delay discounting: ρ = 0.33, 95% CI [0.19-0.46]; emotion recognition: ρ = 0.37, 95% CI [0.24-0.49]; memory: ρ = 0.39, 95% CI [0.25-0.51]) (Table 3 and Appendix Figure A7).’*

- Pages 18-19 lines 519-526: *‘The RT-based speed composite was significantly negatively associated with drift rate for both the delay discounting (ρ = -0.274, p = 0.003) and the memory tasks (ρ = -0.271, p = 0.016). In addition, a positive association was found between the RT-based speed composite and boundary separation for the delay discounting (ρ =0.272, 95% CI [0.12-0.41]) and the memory tasks (ρ = 0.223, 95% CI [0.022-0.41]), and the association between the RT-based speed composite and non-decision time was positive across all three tasks (delay discounting: ρ = 0.19, 95% CI [0.03-0.33]; emotion recognition: ρ = 0.33, 95% CI [0.15-0.49]; memory: ρ = 0.25, 95% CI [0.047-0.43]) (Table 4).’*

*- Pages 19-20 lines 546-548: ‘The bootstrapped unstandardized indirect effect was -1.69e-3 (uncorrected p = 0.036). However, this result did not persist when adjusted for multiple comparisons (p = 0.067).’*

Please note that the original uncorrected indirect effect and p-value for the mediation analysis have been corrected from -1.23e-3 to -1.69e-3 and 0.044 to 0.036, respectively. This change reflects a transcription error identified during the revision process.

**8.** Uneven age distribution limits interpretation of “continuous aging trajectories”

Although the continuous-age approach is preferable to simple young-versus-old comparisons, the sampled age distribution is markedly uneven.

Most participants fall within the 75–84 age range, while middle-aged and oldest-old groups are substantially smaller. This limits the extent to which the analyses can characterize continuous age-related trajectories across the entire sampled range.

Additional descriptive presentation of DDM parameters by age group would help readers evaluate:

• the stability of effects,

• possible nonlinearities,

• and whether findings are disproportionately driven by specific age ranges.

We thank the reviewer for raising this concern. We have now added a Figure to the Appendix (Appendix Figure A6) and added the following sentence:

- Page 16 line 483-484: ‘*Appendix Figure A6 shows mean DDM parameter values per age group (age groups can be found in Table 1 - demographic table).’*

Minor Issues

**1.** Definition of “neurologically healthy” is insufficiently specified

The manuscript states that participants were verified as neurologically normal through multidisciplinary assessment. However, no operational criteria are provided.

The manuscript should clarify:

• neuropsychological cutoffs,

• exclusion criteria,

• whether MCI/subclinical impairment was excluded,

• and how “healthy aging” was defined.

This is particularly important given the advanced age of the cohort.

We thank the reviewer for this comment. The following was added to the manuscript:
- Page 6 lines 157-165: *‘During a consensus conference, physicians and clinicians used the patient history (derived in neurologic exam), informant rated Clinical Dementia Rating (CDR), neuropsychological test results, brain Magnetic Resonance Imaging (MRI), and all available fluid and positron emission tomography (PET) biomarkers to establish the diagnosis. Exclusion criteria for the BRANCH cohort include history or current evidence of: large vessel stroke, diagnosis of DSM-5 major psychiatric disorders, multiple sclerosis, symptomatic neurodegenerative disease, epilepsy, significant memory concerns or related diagnoses, active substance abuse, hepatitis C, HIV, syphilis, blindness, or deafness.’*

**2.** Rationale for young-adult normalization of the RT-based composite is unclear

The RT-based speed composite was normalized using a healthy young-adult reference sample.

Because the present study does not include young adults and focuses only on older participants, the rationale for this normalization is unclear. It would help to clarify whether the normalization was intended merely for scaling purposes or whether the resulting measure should be interpreted as age-relative impairment.

We thank the reviewer for this comment. The RT-based speed composite was derived following the methodology of Kerchner et al. (2012), in which individual task response times are standardized relative to a healthy young adult reference sample and then combined into a composite score. The purpose of this normalization is to provide a standardized measure of processing speed on a common scale and to maintain consistency with the previously validated composite measure. It was not intended to represent age-adjusted impairment within our older adult sample. We have clarified this point in the Methods:

- Page 7 lines 199-202*: ‘The normalization to a young adult reference group was retained to maintain consistency with the validated composite measure described by Kerchner et al. (2012) [2], providing a standardized metric of processing speed rather than an age-adjusted measure of impairment.’*

**3.** Trial trimming procedure deserves further discussion

The exclusion of the slowest 5% of responses was justified by concerns regarding distraction during online testing. While understandable, extremely slow responses may also reflect genuine aging-related variability rather than artifacts.

The potential influence of trimming on DDM parameter estimation should be discussed more explicitly.

We thank the reviewer for this concern. We agree that very slow responses may reflect genuine age-related variability as well as lapses in attention or environmental distractions. The exclusion of the slowest 5% of responses was chosen a priori because the tasks were completed in an unconstrained home environment, where unusually slow responses are more likely to reflect interruptions or off-task processes than in laboratory-based testing.

However, to evaluate the potential influence of this step, we repeated the analyses including all responses (sensitivity analysis in Appendix Tables A1 and A2). The overall pattern of results remained largely unchanged, suggesting that our conclusions are not driven by the exclusion of the slowest 5% of responses. We have also acknowledged now in the Discussion that some very slow responses may represent genuine age-related variability rather than only environmental distraction:

- Page 19 line 527: *‘Table A2 shows results for the sensitivity analysis, without excluding the 5% slowest trials.’*

- Page 25 lines 707-710: *‘A related issue was the exclusion of the slowest 5% of responses, that was intended to reduce the influence of lapses and environmental distractions inherent to unsupervised home testing. However, some of these responses may also reflect genuine age-related variability. Nevertheless, our sensitivity analyses, including all responses, yielded largely comparable results.’*

Strengths

Despite the concerns above, the manuscript has several important strengths:

• preregistration,

• inclusion of oldest-old adults,

• use of continuous age rather than dichotomous grouping,

• explicit model-fit assessment,

• sensitivity analyses,

• computational modelling approach,

• and integration of behavioural and neuroimaging measures.

The model-fit evaluation procedure, including Monte Carlo simulations and graphical diagnostics, is particularly careful and represents a methodological strength of the study.

Overall Evaluation

This manuscript addresses an important topic and includes several valuable methodological features. However, the current interpretation of the findings is, in my view, more conceptually unified and mechanistically specific than the data presently justify.

The manuscript would become substantially stronger if the authors:

• adopted a more cautious interpretation of drift rate,

• more explicitly acknowledged task heterogeneity,

• clarified the theoretical role of the three paradigms,

• reduced mechanistic claims,

• and more carefully addressed the limitations introduced by adaptive task procedures and temporally mismatched multimodal measures.

At present, the data may be better suited to supporting a nuanced account of task-dependent aging effects on DDM-derived parameters than a more general account of age-related slowing of information accumulation.

We thank the reviewer for this constructive overall assessment of our work, as well as for recognizing the strengths of the study. In response to the reviewer's comments, we have revised the manuscript to adopt a more cautious interpretation of the findings, reduce mechanistic language throughout, provide additional methodological details for the experimental paradigms, include new sensitivity and parameter recovery analyses, and expand the discussion of the adaptive task procedures and the temporal separation between behavioral, neuropsychological, and neuroimaging measures. We believe these revisions have strengthened the manuscript and improved the clarity and interpretation of our findings.
