# Supplementary material for: Age-related processes impacting cognitive processing speed across an older adult cohort: A drift diffusion model analysis
Source: PLoS One. 2026 Nov 13;21(11):e0361899. doi: 10.1371/journal.pone.0361899 (PMC13766253; doi:10.1371/journal.pone.0361899)
Supplement: Supplementary file 5 [file pone.0361899.s019.docx]

**Reviewer #1: General Comments**

The authors investigate age-related differences in cognitive processing speed utilizing the drift diffusion model across an older adult cohort. The major weakness of the manuscript centers on the analytical framework applied to the delay discounting task. The methodology utilizes a maximum likelihood estimation to determine a hyperbolic discounting parameter prior to fitting the drift diffusion model. This conditional two-stage procedure creates a structural discrepancy when compared to the accuracy-based coding utilized in the emotion recognition and memory tasks. Consequently, comparing the drift rate parameter across these disparate tasks lacks robust validity. A secondary major weakness relates to the neuroimaging mediation analysis. The sample size decreases substantially for the diffusion tensor imaging models, and the reported mediation effect of fractional anisotropy on the association between age and drift rate in the emotion recognition task did not survive false discovery rate correction. The minor weaknesses involve the data collection environment. The experimental tasks were administered online in unconstrained home settings, which introduces uncontrolled variables such as environmental distractors that directly influence the primary dependent variable of reaction time.

**Scientific Content, Statistics, and Methodology**

The overall scientific content is sound, yet the adaptive difficulty procedures applied to the emotion recognition and memory tasks require careful interpretation. The staircase algorithms were programmed to maintain participant accuracy between 66 and 70 percent. Modifying stimulus difficulty individually means the drift rate parameter measures evidence accumulation under heterogenous testing conditions rather than standardized objective difficulty. The statistical approach appropriately utilizes Spearman correlations for non-parametric assessment of monotonic associations. The authors properly applied the Benjamini-Hochberg false discovery rate procedure for multiple comparisons regarding their primary hypotheses. The methodology of excluding the slowest 5 percent of reaction times and trials faster than 350 milliseconds is a standard and acceptable practice to mitigate the impact of the unsupervised online testing environment. The tract-based spatial statistics correctly utilized permutation testing and threshold-free cluster enhancement, establishing rigorous criteria for the imaging data.

**Specific Comments**

- Page 7, lines 174-181: The methodology permits a time gap of up to four years between online experimental task performance and neuroimaging acquisition. Significant microstructural white matter changes and cortical atrophy occur over a four-year period in cohorts ranging up to 95 years of age, which severely confounds the assumption of cross-sectional alignment between the behavioral and imaging datasets.

- Page 10, lines 240-262: The delay discounting task presents trials immediately after a participant responds without a fixed inter-trial interval or fixation cross. Proceeding without a fixation period fails to control for residual attention or motor preparation from the preceding trial, introducing uncontrolled variance into the non-decision time parameter.

- Page 12, lines 294-319: The memory task dynamically adjusts study time, delay time, and the total number of words based on ongoing performance accuracy. The manuscript lacks an analysis of how these specific adaptive variables independently influence the estimated drift rate, complicating the isolation of pure age-related cognitive slowing from task-induced strategic shifts.

- Page 13, lines 342-352: Calculating a subjective value function before applying the drift diffusion model creates an inconsistent analytical foundation relative to the other assessments. This difference in response coding limits the ability to draw unified conclusions regarding information accumulation across the tested domains.

- Page 24, lines 661-669: The mediation models evaluating global fractional anisotropy present a marginally significant unstandardized indirect effect that became non-significant after adjusting for multiple comparisons. The discussion surrounding white matter connectivity mediating the effects of age on drift rate must be explicitly described as statistically inconclusive.

We are grateful again to the reviewer for their careful and conscientious attention to the manuscript. As this reviewer indicated that previous comments have been addressed, we have not written a point-by-point response to these additional notes. However, our comments to Reviewer 2 indicate several changes made to the manuscript that also address Reviewer 1’s residual concerns. The key remaining issue described by Reviewer 1 concerns the relationship of findings from the delay discounting task to findings from the other two tasks, which is addressed in greater detail below. Their secondary concern is about the interpretation of the exploratory neuroimaging mediation analysis, also addressed in greater detail below. Regarding the home testing environment, we have described our work to address this concern to the extent possible.

Other specific comments are also addressed below, including sensitivity analyses addressing the time gap between experimental task performance and in-person neuroimaging measures and the influence of adaptive variables on drift rate. This reviewer also notes that the lack of a fixation cross or fixed inter-trial interval may introduce variance into the non-decision time parameter. In the revised manuscript, we have further clarified the manuscript’s preregistered focus on the drift rate parameter, and have restated results related to the non-decision time parameter to clarify that hypotheses regarding this parameter were not specifically assessed.

**Reviewer #2:** I appreciate the considerable effort the authors have invested in revising the manuscript. The revision includes several useful additions, including more detailed task descriptions, multiple-comparison correction, reaction-time sensitivity analyses, parameter-recovery analyses, and a more explicit discussion of several limitations. These changes have improved the transparency and reproducibility of the study.

Nevertheless, after comparing the response letter with the revised manuscript and the newly reported analyses, I do not think that all of the central concerns raised in the previous round have been adequately resolved. In several instances, the response provides a rationale for retaining the original approach but does not fully address the underlying construct-validity or interpretational concern. There are also places where the response letter claims that a limitation has been acknowledged or that causal language has been removed, whereas the quoted revised text does not consistently reflect that change.

My principal remaining concerns are outlined below.

**1**. The interpretation of drift rate across the three tasks remains overly general

The authors provide a reasonable justification for applying the drift diffusion model to heterogeneous cognitive tasks. I agree that DDMs have been productively applied to perceptual, mnemonic, and value-based decisions, and that it can be informative to examine DDM parameters across different tasks within the same cohort.

However, this does not resolve the construct-validity issue raised in the previous review. The fact that the same mathematical model can be applied to different tasks does not establish that drift rate has the same psychological meaning across those tasks. In the present study, drift rate may reflect:

• perceptual-social evidence in the emotion-recognition task;

• mnemonic evidence, familiarity, or retrieval processes in the memory task; and

• subjective-value differences, decision conflict, or preference consistency in the delay-discounting task.

These can all be described as forms of evidence accumulation, but they should not automatically be treated as interchangeable measures of a unitary cognitive processing-speed construct.

The authors now state that their argument does not depend on the three operations being psychologically equivalent. This is an important concession, but it is not fully reconciled with the broader framing and conclusions of the manuscript, which continue to interpret drift rate as a relatively general measure of information-processing or cognitive processing speed. If the tasks are not assumed to be psychologically equivalent, the corresponding drift rates should instead be described consistently as task-specific indices of evidence accumulation.

A stronger claim about a common, task-general processing-speed construct would require relevant convergent evidence, such as substantial correlations among the task-specific drift rates, formal latent-variable modelling, or another analysis demonstrating shared variance. In the absence of such evidence, the study can still make a useful contribution, but its contribution should be framed as examining age-related associations with task-specific DDM parameters across heterogeneous decision contexts.

This issue is particularly important for delay discounting. The participant-specific discounting parameter (k) is estimated from the choices, and the same choices are subsequently classified as consistent or inconsistent with the preference function defined by that estimate. Drift rate in this model is therefore likely to reflect the consistency or certainty of choices relative to the fitted preference function, rather than cognitive processing speed in the same sense as in a perceptual or memory decision. The analogy between preference-consistent and objectively correct responses does not eliminate this conceptual difference.

The manuscript should therefore:

• avoid presenting the three drift-rate estimates as directly comparable measures of a single processing-speed construct;

• describe their psychological interpretation separately for each task;

• state explicitly that the evidence for a common age-related mechanism across tasks was exploratory and inconclusive; and

• revise the Abstract, Introduction, Discussion, and conclusions accordingly.

We thank the reviewer for this comment and agree that application of the same mathematical model across heterogeneous tasks does not imply that *drift rate* has an identical psychological meaning across tasks. To directly examine the extent of shared variance, we have now conducted exploratory pairwise Spearman correlations *between drift rate* estimates from the three paradigms. *Drift rates* were correlated between the *memory* and *emotion recognition* tasks (ρ = 0.32, 95% CI [0.18, 0.46]) and between the *emotion recognition* and *delay discounting* tasks (ρ= 0.25, 95% CI [0.05, 0.43]), whereas the association between *memory* and *delay discounting* *drift rates* was smaller and not statistically significant (ρ = 0.12, 95% CI [-0.1, 0.32]; these two tasks had the smallest overlap in participants) (these analyses have been added to the Appendix Figure A7). Thus, these analyses provide some evidence of shared variance, but indeed do not support treating the three *drift rate* estimates as interchangeable measures of a unitary processing-speed construct.

We have revised the manuscript accordingly. *Drift rate* is now interpreted in relation to perceptual/social evidence in the *emotion recognition* task, mnemonic evidence in the *memory* task, and subjective-value-related evidence in the *delay discounting* task. We have also revised the broader framing and conclusions to describe the study as examining age-related associations with task-specific information processing across heterogeneous cognitive contexts.

- Page 1 lines 26-27: *‘Our results suggest age-related differences in information processing across cognitive contexts…’*

- Page 5 line 129: *‘ … - emotion recognition, memory, and delay discounting - …’*

- Page 5 lines 132-137: ‘*We primarily evaluated the effects of age on the drift rate parameter, as it reflects the rate of information accumulation during decision-making. Although drift rate provides a common mathematical parameter of information accumulation across the three tasks, the nature of the information being accumulated varies with task: perceptual/social information in the emotion recognition task, mnemonic information in the memory task, and information related to subjective valuation in the delay discounting task.’*

*-* Page 14 lines 407-410: *‘Additionally, we conducted exploratory pairwise Spearman correlations between drift rate estimates from the three tasks to assess the degree of correspondence in drift rate across task contexts*. These are *reported descriptively as effect sizes with 95% confidence intervals.’*

- Page 17 lines 505-506: ‘*Appendix Figure A7 shows the pairwise Spearman correlations between drift rate estimates from the three tasks.’*

- Pages 23 lines 630-632: ‘… *(reflecting slower information accumulation within a given task context)* *with evidence for this association observed in two of the three cognitive domains examined.* ’

- Pages 26 lines 729-730: ‘…*information processing across cognitive contexts and that these task-specific processes should be considered…’*

- Page 28 lines 775-778: *‘Indeed, the modest and inconsistent pairwise associations observed between drift rates across tasks suggest that these parameters may be interpreted as task-specific indices of information accumulation rather than as interchangeable measures of a unitary processing-speed construct.’*

- Page 29 lines 816-817: *‘…reflected in task-specific patterns of evidence accumulation across cognitive domains…’*

**2.** The implications of the adaptive task procedures remain insufficiently addressed

The revised Methods provide substantially more information about the adaptive procedures, which is helpful. However, the central interpretational problem remains.

The response letter states that the revised Discussion acknowledges that adaptive procedures “may nevertheless limit the direct comparability of drift rate estimates across individuals.” Yet the quoted text added to the manuscript does not contain this acknowledgement. Instead, it concludes that drift rate should be interpreted as evidence-accumulation efficiency “under task conditions individually adjusted to achieve comparable levels of performance.”

Comparable accuracy does not necessarily imply comparable stimulus difficulty or comparable latent decision processes. Participants may achieve similar accuracy while being exposed to different emotion intensities, list lengths, encoding durations, or retention conditions. Because drift rate is sensitive to stimulus discriminability and task difficulty, individual differences in the adaptive levels attained may directly influence the estimated drift rates.

This is especially problematic in the memory task, in which more than one task dimension appears to be adapted. It is therefore difficult to determine whether individual differences in drift rate reflect evidence-accumulation efficiency, memory ability, the task difficulty reached by the staircase, or some combination of these factors.

If the necessary task-level variables are available, the authors should report:

• the distribution of the mean or final difficulty levels;

• their associations with age;

• their associations with drift rate; and

• whether the age–drift-rate associations remain after accounting for achieved task difficulty.

If these analyses cannot be performed, the limitation should be stated directly and more strongly. The manuscript should not imply that equating average performance necessarily makes drift-rate estimates directly comparable across individuals.

We thank the reviewer for this comment and agree that maintaining comparable accuracy across participants does not necessarily imply equivalent stimulus difficulty, and that variation in the adaptive levels attained may contribute to individual differences in *drift rate.*

We have now performed the suggested additional analyses for both tasks. Specifically, we examined the distributions of the final difficulty levels, their associations with age and *drift rate*, and whether the associations between age and *drift rate* remained after accounting for achieved task difficulty.

For the emotion recognition task, the association between final adaptive level and age was ρ = 0.21 (95% CI [0.08, 0.35]), while the association between final adaptive level and drift rate was ρ = −0.15 (95% CI [−0.29, −0.01]). For the memory task, the corresponding associations were ρ = 0.12 (95% CI [−0.03, 0.27]) and ρ = −0.24 (95% CI [−0.38, −0.10]), respectively. Lower values of the adaptive parameters correspond to greater task difficulty. Importantly, partial Spearman correlations controlling for task difficulty showed that the negative association between age and *drift rate* remained significant for both the *emotion recognition* (ρ = −0.16, p = .044) and *memory* (ρ = −0.16, p = .048) tasks.

Regarding the reviewer’s specific concern that multiple dimensions were adapted in the *memory* task, we have added to the task description to clarify that only two dimensions were independently adapted, word count and study time (delay time was adjusted linearly with study time to maintain a combined time of 30 seconds). Although both word count and study time were adjusted during the adaptive procedure, all participants reached the maximum word count. Word count therefore was not a source of between-participant variation in final achieved difficulty, and final study time, which continued to vary across participants, was used to characterize task difficulty in these analyses.

These additional analyses indicate that achieved task difficulty is related to *drift rate*, congruent with the reviewer’s concern that the adaptive levels attained could contribute to individual differences in *drift rate* estimates. However, the persistence of the age–*drift rate* associations after accounting for achieved task difficulty suggests that variation in final task difficulty alone does not account for these associations. We have added these analyses to the Methods and Results, included the distributions of final difficulty levels in Appendix Figure A9 and revised the Discussion accordingly. The following changes have been made to the manuscript:

- Page 11 lines 306-307: *‘Delay time was adjusted according to the increase or decrease in study time to keep the combined time at 30 seconds.’*

- Page 14 lines 410-420: *‘As a further check, to assess the potential influence of the adaptive difficulty procedures of the emotion recognition and the memory tasks on drift rate, we examined the distributions of achieved task difficulty and its associations with age and drift rate for both tasks. These are reported as effect sizes with 95% confidence intervals. For the memory task, although both word count and study time were adjusted by the adaptive procedure, all participants reached the maximum word count. Therefore, individual differences in final achieved difficulty were characterized using final study time, with shorter study times indicating greater difficulty. In a sensitivity analysis, we performed partial Spearman correlations between age and drift rate while controlling for achieved task difficulty. These associations were adjusted for multiple comparisons using the Benjamini–Hochberg FDR procedure correcting for the same number of associations as in the main analyses).’*

- Pages 19-20 lines 536-547: *‘Given the adaptive nature of the emotion recognition and memory tasks, we additionally examined whether the task difficulty achieved by participants was associated with age and drift rate. In both tasks, lower values of the adaptive parameter represented greater task difficulty. In the emotion recognition task, age was positively associated with the final adaptive level (ρ = 0.21, 95% CI [0.08,0.35]) whereas drift rate was negatively associated with the final adaptive level (ρ = -0.15, 95% CI [-0.29, -0.01]). In the memory task, the association between age and final adaptive level was ρ = 0.12 (95% CI [−0.03, 0.27]), while drift rate was negatively associated with the final adaptive level (ρ = −0.24, 95% CI [−0.38, −0.10]). In sensitivity analyses, partial Spearman correlations controlling for achieved task difficulty showed that the negative association between age and drift rate remained significant for both emotion recognition (ρ = -0.16, p = 0.044) and memory (ρ = -0.16, p = 0.048) tasks. The distributions of achieved task difficulty are displayed in Appendix Figure A9.’*

- Pages 24-25 lines 672-681*: ‘The additional analyses showed that participants with higher drift rates tended to reach more difficult task conditions in both tasks, confirming that achieved difficulty is associated with individual differences in drift rate estimates. Older age was also associated with reaching less difficult conditions in the emotion recognition task,* *although the confidence interval for the association between age and difficulty included zero in the memory task. Importantly, however, the negative associations between age and drift rate remained significant after accounting for achieved task difficulty in both tasks, suggesting that differences in achieved difficulty alone did not account for the observed age–drift rate associations. Nevertheless, because participants were exposed to different task conditions, direct comparison of drift rate estimates across individuals should be made with caution.’*

**3.** The temporal mismatch has not been adequately addressed by the reported sensitivity analysis

It is useful that the difference in days between DTI acquisition and online task performance was included as a covariate in the mediation analysis. However, the additional sensitivity analysis does not directly test the effect of temporal mismatch.

Restricting the sample to participants who remained cognitively and neurologically healthy at a subsequent clinical evaluation addresses the possibility of overt intervening neurological or cognitive disorder. It does not establish that white matter integrity or processing speed remained stable during the three- to four-year interval between measurements. Normal age-related changes can occur even in individuals who remain clinically healthy.

Moreover, the manuscript’s statement that the pattern of associations was “generally consistent with the primary analyses” is not well supported by Appendix Table A3. For the association between the RT-based speed composite and drift rate, the estimates changed as follows:

• delay discounting: approximately (\rho=-.27) to (\rho=-.17);

• emotion recognition: approximately (\rho=-.09) to (\rho=+.08);

• memory: approximately (\rho=-.27) to (\rho=-.05).

Thus, two associations were substantially attenuated and one changed direction. This cannot be described solely as a loss of statistical significance caused by wider confidence intervals. The Results and Discussion should report this attenuation accurately.

Ideally, the authors should conduct sensitivity analyses using progressively narrower measurement intervals, even if the resulting estimates are imprecise, or model the temporal difference continuously in all analyses involving non-concurrent measures. At minimum, the manuscript must clearly distinguish between sensitivity to subsequent clinical status and sensitivity to measurement timing, as these are not equivalent.

The mediation sensitivity result reported for the reduced sample, (p=.006), should also be identified explicitly as either corrected or uncorrected. Its inferential status is currently unclear.

We thank the reviewer for this clarification and agree that our previous sensitivity analysis addressed the potential influence of intervening clinical neurological or cognitive conditions rather than the temporal mismatch between measurements itself. We have revised the manuscript to distinguish these two issues explicitly. We have also revised our description of the results from this analysis. In particular, associations between the RT-based speed composite and *drift rate* were attenuated in the restricted sample, with the emotion-recognition association also changing direction. We therefore no longer describe these results as generally consistent with the primary analyses or attribute the differences solely to reduced statistical power. We have also clarified the inferential status of the mediation result in the reduced sample: the reported p = 0.003 was FDR-corrected (note the change from 0.006 to 0.003 due to the correction for 4 associations instead of 10).

As suggested by the reviewer, to address measurement timing more directly, successive sensitivity analyses were performed utilizing progressively narrower time windows for linkage to in-person neuropsychological and neuroimaging testing, which are now displayed in Appendix Figure A10 and A11.

The following changes were made to the manuscript:

- Pages 15 lines 431-436: *‘To assess the potential influence of intervening changes in clinical status during the time gap between the online task evaluation and the RT-based speed composite and neuroimaging acquisitions, sensitivity analyses for hypothesis ii and exploratory hypothesis iii were performed including only participants for whom a subsequent longitudinal evaluation following completion of the online tasks was available and confirmed that they remained cognitively and neurologically healthy.’*

*- Page 15 lines 439-445: ‘To directly assess the potential influence of temporal mismatch between measurements, additional sensitivity analyses were performed utilizing progressively narrower time windows for linkage to in-person neuropsychological and neuroimaging testing. For analyses involving the RT-based speed composite, these time windows were ≤1 year, ≤2 years, and (as originally preregistered) ≤3 years; for analyses involving neuroimaging measures, these time windows were ≤1 year, ≤ 2 years, ≤3 years, and (as originally preregistered) ≤4 years.* *These are presented graphically with Confidence Intervals.’*

*-* Page 20 lines 565-569*: ‘In this sensitivity analysis, associations between the RT-based speed composite and drift rate were attenuated relative to the primary analyses for the delay discounting and memory tasks, while the association for the emotion recognition task was weak and changed direction. Confidence intervals were also wider, reflecting the substantially reduced sample sizes, and none of the associations were statistically significant.’*

*-* Page 20 lines 569-574*: ‘ Appendix Figure A10 demonstrates the sensitivity analysis focusing on temporal mismatch between task performance and in-person neuropsychological testing for the elements included in the RT-based speed composite. Because of restrictions on in-person evaluation during the COVID-19 pandemic, no participants had in-person evaluations within 1 year of the emotion recognition or memory tasks. and few had in-person evaluations within 2 years of these tasks.’*

- Page 21 line 594: *‘…p=0.003, after correcting for multiple comparisons.’*

- Page 21 lines 594-597*: ‘Appendix Figure A11 demonstrates the sensitivity analysis focusing on temporal mismatch between task performance and in-person neuroimaging; the direction and magnitude of indirect effects were not substantially changed by the use of narrower time windows.’*

- Page 25 lines 705-707: *‘However, associations between the RT-based speed composite and drift rate were attenuated or changed direction in the restricted sensitivity analysis, warranting caution in interpreting these findings, particularly given the temporal separation between assessments.’*

**4.** The mediation result remains overinterpreted and is described inconsistently

The authors report an uncorrected indirect-effect (p)-value of .036, which becomes .067 after multiple-comparison correction. Accordingly, this result does not meet the inferential criterion adopted in the revised manuscript.

Despite this, the manuscript continues to use statements such as:

• “Global FA showed a significant indirect effect”;

• “white matter microstructure changes have a significant indirect effect”;

• “this association is partially accounted for by changes in white matter microstructure”; and

• “global FA might partially account for the association.”

These formulations are not fully consistent with the corrected result. Some also continue to imply a mechanistic or causal chain, despite the cross-sectional design and temporal separation of the measures. The response letter states that causal language has been changed throughout the manuscript, but several of the examples presented as revised wording still retain mechanistic implications.

The Abstract formulation—“we show that this association … might be partially accounted for by global fractional anisotropy”—is also too strong. The phrase “we show” is difficult to reconcile with an exploratory effect that did not survive correction.

A more accurate formulation would be:

“An exploratory indirect association involving global FA was observed in the emotion-recognition task, but it did not remain statistically significant after correction for multiple comparisons.”

Similar wording should be used consistently in the Results and Discussion. The authors should avoid describing the effect as significant without immediately specifying that this refers only to the uncorrected result. They should also avoid “account for,” “mediate the effects of age,” or equivalent mechanistic language.

The result may be reported as hypothesis-generating, but it should not support substantive conclusions about white matter explaining age-related changes in drift rate.

We thank the reviewer for this comment and agree that our previous wording remained too strong given that the indirect effect did not remain statistically significant after correction for multiple comparisons. We have therefore revised the manuscript throughout to clearly distinguish the uncorrected exploratory finding from the corrected result and have removed language implying that global FA accounts for or mediates the association between age and *drift rate*. Specifically, the indirect association involving global FA in the *emotion recognition* task is now reported as an exploratory finding that was observed at the uncorrected level (p = 0.036) but did not remain statistically significant following FDR correction (p = 0.134). We have also revised the Abstract, Results, Discussion, and Conclusion to avoid mechanistic or causal interpretations and to describe this finding as hypothesis-generating.

The following changes have been made to the manuscript:

- Page 1 lines 24-26: *‘…in an exploratory analysis, the association for the emotion recognition task was mediated by global fractional anisotropy but this did not survive multiple-comparison correction.’*

- Page 21 lines 585-589: *‘An exploratory indirect association involving global FA was observed between age and drift rate in the emotion recognition task. Unstandardized indirect effects were computed for each of 10000 bootstrapped samples. The bootstrapped unstandardized indirect effect was -1.69e-3 (uncorrected p = 0.036) but did not remain statistically significant after correcting for multiple comparisons (p = 0.134).’*

- Pages 23 lines 622-625: *‘…our exploratory analysis for the association between age and drift rate in the emotion recognition task produced evidence for mediation via global FA, although this did not remain statistically significant after correction for multiple comparisons.’*

- Page 26 lines 713-714: *‘…identified an exploratory indirect association involving global FA in the association between age and drift rate in the emotion recognition task.’*

- Page 26 line 715: *‘This finding is consistent with…’*

- Page 26 lines 717-718: *‘…global FA may be particularly relevant for investigating the relationship between age and drift rate.’*

- Page 26 lines 718: *‘However, given that the finding we report, …’*

**5.** The conclusions concerning differences between tasks should be further softened

The authors appropriately acknowledge that a significant association in two tasks and a nonsignificant association in a third task do not demonstrate a statistically significant difference between tasks. This is a welcome improvement.

Nevertheless, expressions such as “may be more evident in some cognitive domains than others” and “may be more apparent” still invite a between-task interpretation that was not formally tested. Given that the delay-discounting association was in the same direction as the other associations, and given the differences in task architecture, response coding, trial numbers, and adaptive procedures, the safest conclusion is that evidence for an association was obtained in two tasks but not in the third. The data do not establish that the population-level associations differ across tasks.

The manuscript should distinguish clearly between:

• evidence for an association within each task; and

• evidence that the magnitude of the association differs between tasks.

Only the former was tested.

We thank the reviewer for this comment. We have now removed statements suggesting that the age–*drift rate* association may be “more evident” or “more apparent” in some cognitive domains than others. Throughout the manuscript, we now distinguish between evidence for an association within individual tasks and evidence for differences in the magnitude of associations across tasks. Specifically, we report that evidence for an association between age and *drift rate* was observed in the *emotion recognition* and *memory* tasks but not in the *delay discounting* task, while explicitly noting that between-task differences in the magnitude of these associations were not formally tested.

- Page 1 lines 26-27: *‘Our results suggest age-related differences in information processing within cognitive contexts…’*

- Page 23 lines 630-632: *‘…(reflecting slower information accumulation within a given task context) with evidence for this association observed in two of the three cognitive domains examined.’*

- Page 29 lines 816-817: *‘…reflected in task-specific patterns of evidence accumulation across cognitive domains…’*

**6.** The delay-discounting model requires further clarification

The authors appropriately acknowledge that the delay-discounting DDM is conditional on the first-stage estimate of (k), and they now discuss joint hierarchical modelling as a preferable future approach. This improves the manuscript.

However, two additional issues should be clarified.

First, the Methods indicate that (k) was estimated with bounds of (-10^{10}) and (10^{10}). A standard hyperbolic temporal-discounting parameter is ordinarily constrained to a theoretically meaningful non-negative range. Allowing very large negative values may create theoretically invalid discount functions and potential singularities in (V=A/(1+kD)). The authors should justify these bounds, report whether any negative estimates occurred, and preferably repeat the estimation using theoretically appropriate constraints.

Second, because (k) is estimated from the same choices that are subsequently coded as consistent or inconsistent, the resulting DDM parameter should be interpreted cautiously. It is not equivalent to a drift rate estimated from independently defined correct and incorrect responses. This difference needs to be reflected not only in the limitations but also in the substantive interpretation of the delay-discounting results.

We thank the reviewer for raising this point. We agree that the hyperbolic discounting parameter is theoretically non-negative and that the broad bounds used in our original numerical optimization were unnecessarily permissive. We therefore inspected the estimated *k* values. Of the negative estimates, all but two were extremely small (roughly 10⁻¹⁵) and can be considered numerical approximations to zero. Two participants had negative estimates of *k* = −0.019. We have clarified the original optimization procedure and the occurrence of negative estimates in the Methods.

We also agree that the *delay discounting* task *drift rate* differs conceptually from that obtained from tasks with independently defined correct and incorrect responses. We have further clarified throughout the manuscript that responses in this task were classified according to consistency with each participant's individually estimated discounting function, which was itself estimated from the same choice data. Accordingly, *delay discounting drift rate* is interpreted as evidence accumulation toward choices consistent with the individually estimated preference model, rather than evidence accumulation toward an independently defined correct response.

The following changes were made to the manuscript:

- Page 12 lines 346-350: *‘…to avoid imposing constraints on the numerical optimization. Although this allowed negative k estimates, inspection of the estimated values showed that 25 negative estimates were extremely small (on the order of 10^-15^-10^-16^) and were considered numerical approximations to zero, while two participants had negative estimates (k = −0.019).’*

- Page 12 lines 355-357*: ‘However, because this tendency was estimated from the same choices subsequently classified as consistent or inconsistent based on the estimated k, the resulting drift rate could differ from that obtained when response accuracy is defined independently of participants’ choices.’*

- Page 24 lines 658-661: *‘Moreover, because k was estimated from the same choices that were subsequently classified as consistent or inconsistent, the resulting drift rate may not be equivalent to one estimated from independently defined correct and incorrect responses.’*

**7.** The parameter-recovery analysis is useful but should not be overstated

The addition of a parameter-recovery analysis is welcome. Nevertheless, high correlations between empirical parameter estimates and estimates recovered from data simulated from those same fitted parameters do not provide a complete assessment of parameter recovery.

Correlations can be high when there is substantial between-participant variation even if estimates are systematically biased or imprecise. A more informative evaluation would also report measures such as bias, root mean squared error, regression slope and intercept, or interval coverage, ideally across repeated simulations.

If the authors retain the current analysis, it should be described more narrowly as evidence that the rank ordering of participants was reasonably recoverable under the fitted model and observed trial counts, rather than as comprehensive evidence of “good recovery.” The inability to assess test–retest reliability is understandable and should remain acknowledged.

We thank the reviewer for this clarification. We have now revised the Methods, Results, and figure caption to describe the analysis more narrowly and no longer characterize the observed correlations as demonstrating “good parameter recovery.” Instead, we interpret them as showing that the rank ordering of participants was well recovered under the fitted model and observed trial counts.

The following changes were made to the manuscript:

- Page 14 lines 392-393: *‘…, to assess preservation of the rank ordering of parameter values.’*

- Page 17 lines 500-501: *‘…the rank ordering of the estimated DDM parameters was well preserved across all three tasks, …’*

**8.** Multiple-comparison correction is improved but requires clearer reporting

The introduction of FDR correction represents an important improvement. However, the correction procedure and the status of the reported p-values should be described more consistently.

The Methods should state clearly which families of tests were subjected to FDR correction and whether the p-values subsequently reported in the text, tables, and figures are adjusted unless explicitly identified otherwise. Standalone tables and figure captions should also provide sufficient information to be interpreted independently. For example, the Figure 1 caption should refer to an FDR-adjusted significance threshold rather than simply stating that associations were significant “at a p-value of 0.05.” Unadjusted p-values may be reported for transparency, but all inferential conclusions should be based on the adjusted values.

The following points also require clarification:

• whether the p-values reported for the sensitivity analyses are adjusted or unadjusted;

• whether the mediation sensitivity result of (p=.006) is adjusted or unadjusted;

• the exact tests included in each FDR family; and

• whether the ancillary mediation analyses involving boundary separation and non-decision time were included in any multiplicity correction

In addition, the rationale for combining six preregistered behavioural associations and four exploratory mediation tests into a single ten-test FDR family is unclear. These tests address conceptually distinct hypotheses, and the mediation analyses appear to have been conducted conditionally, following evidence of associations in the preceding analyses. Combining them into one family may therefore allow the rank ordering of strong behavioural p-values to influence the adjustment of conceptually separate exploratory mediation results. The authors should either provide a clear statistical and conceptual justification for this combined family or apply separately defined correction families to the behavioural and mediation analyses. If the correction procedure is revised, all affected adjusted p-values and corresponding conclusions should be updated accordingly.

We thank the reviewer for this comment. We agree that combining the preregistered behavioral associations and exploratory mediation analyses into a single FDR family was not sufficiently justified given that these analyses address conceptually distinct hypotheses. We have therefore revised the multiple comparison procedure and applied Benjamini–Hochberg FDR correction separately to the four exploratory mediation analyses involving *drift rate*. All affected adjusted p-values and corresponding conclusions have been updated accordingly. We have also revised the Methods, tables and figure captions to explicitly identify the tests included in each correction family and whether reported p-values are adjusted or unadjusted.

Finally, given that we now report associations between boundary separation and non-decision time with age descriptively as effect sizes and 95% Confidence Intervals and do not associate them with p-values or significance-based inference, we have removed analyses involving these parameters from the mediation analyses.

The following changes were made to the manuscript:

- Page 14 lines 404-406: *‘These ancillary analyses were not included in the FDR correction, as they were treated as ancillary and are reported descriptively as effect sizes with 95% confidence intervals, without p-values or significance-based inference.’*

- Page 15 lines 436-437: *‘All sensitivity analyses followed the same FDR adjustments for multiple comparisons as the main analysis.’*

- Page 15 lines 437-438: *‘All p-values are reported after adjustment for multiple comparisons unless explicitly stated otherwise.’*

- Page 19 lines 530-531 (Figure 1 legend): *‘…at an FDR-adjusted p-value of 0.05 using the Benjamini–Hochberg procedure…’*

- Page 19 lines 534-535 (Table 3 legend): *‘P-values for drift rate are Benjamini–Hochberg FDR-adjusted.’*

- Page 21 lines 578-579 (Table 4 legend): *‘P-values for drift rate are Benjamini–Hochberg FDR-adjusted.’*

**9.** Issues that have been addressed adequately

Several previous concerns have been addressed sufficiently and, in my view, need not prevent publication after the remaining issues are resolved.

The authors have:

• substantially expanded the task descriptions;

• clarified the definition and clinical evaluation of neurologically healthy participants;

• explained the purpose of normalization to the young-adult reference sample;

• replaced longitudinal terminology with cross-sectional terminology;

• acknowledged the uneven age distribution and limited oldest-old sample;

• added descriptive presentation by age group;

• reported diffusion-specific quality-control procedures and motion statistics;

• provided reaction-time distributions before and after trimming;

• repeated the principal analyses without trimming the slowest 5% of trials; and

• justified the preregistered use of Spearman correlations as tests of monotonic associations.

I consider the explanation for not fitting nonlinear age trajectories broadly acceptable. A nonlinear exploratory analysis could still be informative, but it is not essential to the preregistered monotonic association hypothesis, provided that the manuscript no longer makes claims about developmental or aging trajectories.

**Overall assessment**

The manuscript has improved, and I recognize that the authors have made a serious effort to respond to a large number of comments. The study has meaningful strengths, including preregistration, a relatively large older-adult sample, explicit model-fit diagnostics, sensitivity analyses, and the integration of behavioural modelling with neuroimaging measures.

However, several central concerns have not yet received satisfactory answers. In particular, the manuscript still moves too readily from task-specific DDM parameters to a general cognitive processing-speed interpretation; the consequences of adaptive task difficulty are not adequately analysed or acknowledged; the temporal sensitivity analysis does not test temporal proximity and is described more positively than its results warrant; and the exploratory, non-significant-after-correction mediation effect continues to be presented using significant and partly mechanistic language.

Therefore, I would support a further substantial revision focused on aligning the claims with what the analyses can establish. The study may ultimately support a useful and appropriately nuanced account of age-related associations with task-specific DDM parameters. It does not currently provide convincing evidence for a unitary, task-general slowing of evidence accumulation or for white matter microstructure as a mechanism explaining that slowing.
