# Supplementary material for: Age-related processes impacting cognitive processing speed across an older adult cohort: A drift diffusion model analysis
Source: PLoS One. 2026 Nov 13;21(11):e0361899. doi: 10.1371/journal.pone.0361899 (PMC13766253; doi:10.1371/journal.pone.0361899)
Supplement: Supplementary file 6 [file pone.0361899.s020.docx]

**Reviewer #2**

I appreciate the considerable effort the authors have invested in this revision. The additional analyses and the more careful interpretation have addressed the great majority of my previous concerns, and the manuscript is now substantially clearer, more transparent, and appropriately nuanced. In particular, the task-specific interpretation of drift rate, the analyses of adaptive task difficulty and temporal mismatch, and the revised multiple-comparison procedure represent important improvements. I have only two remaining points, both of which should be relatively straightforward to address.

**1. Interpretation of the mediation analysis**

The indirect association involving global FA was no longer statistically significant after correction for multiple comparisons: the uncorrected p-value was .036, whereas the FDR-adjusted p-value was .134. Nevertheless, the Abstract still states that the association “was mediated by global fractional anisotropy,” and the Discussion refers to “evidence for mediation.” These formulations remain stronger than warranted by an exploratory result that did not remain significant after correction, particularly given the cross-sectional design.

The white-matter finding need not be omitted entirely, as it remains an interesting, hypothesis-generating observation. However, it should be described consistently as an exploratory indirect association that was not statistically significant after correction. The Conclusion may mention it in this explicitly qualified form rather than stating more generally that age-related slowing “might be linked to age-related loss of white matter integrity.” The term “white matter integrity” should also be avoided or qualified, given the acknowledged limitations of interpreting FA as a direct measure of integrity.

We thank the reviewer for this comment and agree that the previous wording remained too strong given that the exploratory indirect association involving global FA did not remain statistically significant after multiple-comparison correction. We have revised the Abstract, Discussion, and Conclusion to consistently describe this finding as an exploratory indirect association that did not remain statistically significant after correction, rather than as evidence of mediation. The finding is now presented explicitly as hypothesis-generating and as requiring further investigation.

The following changes were made to the manuscript:

- Page 1 lines 24-26*: ‘in an exploratory analysis, an indirect association involving global fractional anisotropy was observed for the emotion recognition task, although this did not survive multiple-comparison correction.’*

- Page 23 line 631: *‘…identified an indirect association involving global FA,* *although this did not remain statistically significant after correction for multiple comparisons.’*

*- Page 29 lines 823-826: ‘An exploratory indirect association involving global FA, although not statistically significant after multiple-comparison correction, suggests that the potential role of age-related differences in white matter microstructure warrants further investigation.’*

**2. Negative discounting-parameter estimates**

Inspection of the estimated discounting parameters revealed two genuinely negative values (\(k=-.019\)), in addition to very small negative values that can reasonably be regarded as numerical approximations to zero. Because the hyperbolic discounting parameter is theoretically expected to be zero or positive, merely reporting the negative estimates does not fully resolve the issue.

The authors should preferably repeat the estimation while restricting \(k\) to non-negative values and confirm whether the classification of responses, the delay-discounting DDM estimates, and the substantive conclusions remain unchanged. At minimum, they should provide a sensitivity analysis excluding the two participants with clearly negative estimates.

Subject to these two targeted revisions, I believe the manuscript would be suitable for publication.

We thank the reviewer for this suggestion. We performed the requested sensitivity analysis excluding the two participants with clearly negative estimates of the discounting parameter (k = −0.019). The results were essentially unchanged. The Spearman correlation between age and *drift rate* was ρ = −0.097 (p = 0.27) after excluding these participants, compared with ρ = −0.098 (p = 0.25) in the full sample. Similarly, the correlation between the RT-based speed composite and *drift rate* was ρ = −0.27 (p = 0.005) after exclusion, similar to that observed in the full sample (ρ = −0.274, p = 0.003). Thus, exclusion of the two participants with clearly negative *k* estimates did not affect the substantive conclusions for the *delay-discounting* task.

We have added this sensitivity analysis to the manuscript:

- Page 14 lines 407-409: ‘*Moreover, for the delay-discounting task, a sensitivity analysis for the associations of drift rate with age and the RT-based speed composite was performed after excluding the two participants with k = −0.019.’*

*-* Page 18 lines 530-531*: ‘Excluding the two participants with clearly negative k estimates did not noticeably alter the delay-discounting task results (ρ = −0.097, p = 0.27).’*

*- Page 20 lines 567-569: ‘Excluding the two participants with clearly negative k estimates did not noticeably alter the delay-discounting task results (ρ = −0.27, p = 0.005).’*
