# Supplementary material for: Age-related processes impacting cognitive processing speed across an older adult cohort: A drift diffusion model analysis
Source: PLoS One. 2026 Nov 13;21(11):e0361899. doi: 10.1371/journal.pone.0361899 (PMC13766253; doi:10.1371/journal.pone.0361899)
Supplement: Peer review history

# Peer review: Decision Letter 0

**DOI:** 10.1371/journal.pone.0361899.r001

**Authors:** Pramod Yadav

25 May 2026

Thank you for submitting your manuscript to PLOS One. The manuscript has now been evaluated by expert reviewers. Based on the reviewers’ comments and recommendations, I would like to inform you that the decision on your manuscript is Major Revision.

The reviewers have identified several important issues that should be carefully addressed before the manuscript can be considered further for publication. I encourage you to revise the manuscript thoroughly in accordance with the reviewers’ suggestions and comments.

plosone@plos.org. When you're ready to submit your revision, log on to https://www.editorialmanager.com/pone/ and select the 'Submissions Needing Revision' folder to locate your manuscript file.

- A letter that responds to each point raised by the academic editor and reviewer(s). You should upload this letter as a separate file labeled 'Response to Reviewers'.
- A marked-up copy of your manuscript that highlights changes made to the original version. You should upload this as a separate file labeled 'Revised Manuscript with Track Changes'.
- An unmarked version of your revised paper without tracked changes. You should upload this as a separate file labeled 'Manuscript'.

If you would like to make changes to your financial disclosure, please include your updated statement in your cover letter. Guidelines for resubmitting your figure files are available below the reviewer comments at the end of this letter.

If applicable, we recommend that you deposit your laboratory protocols in protocols.io to enhance the reproducibility of your results. Protocols.io assigns your protocol its own identifier (DOI) so that it can be cited independently in the future. For instructions see: https://journals.plos.org/plosone/s/submission-guidelines#loc-laboratory-protocols. Additionally, PLOS ONE offers an option for publishing peer-reviewed Lab Protocol articles, which describe protocols hosted on protocols.io. Read more information on sharing protocols at https://plos.org/protocols?utm_medium=editorial-email&utm_source=authorletters&utm_campaign=protocols.

As the corresponding author, your ORCID iD is verified in the submission system and will appear in the published article. PLOS supports the use of ORCID, and we encourage all coauthors to register for an ORCID iD and use it as well. Please encourage your coauthors to verify their ORCID iD within the submission system before final acceptance, as unverified ORCID iDs will not appear in the published article. *Only*  the individual author can complete the verification step; PLOS staff *cannot*  verify ORCID iDs on behalf of authors.

We look forward to receiving your revised manuscript.

Kind regards,

Pramod K. Yadav, Ph. D.

Academic Editor

PLOS One

**Journal Requirements:**

When submitting your revision, we need you to address these additional requirements.

1. Please ensure that your manuscript meets PLOS ONE’s style requirements, including those for file naming. The PLOS ONE style templates can be found at

https://journals.plos.org/plosone/s/file?id=wjVg/PLOSOne_formatting_sample_main_body.pdf and

https://journals.plos.org/plosone/s/file?id=ba62/PLOSOne_formatting_sample_title_authors_affiliations.pdf

2. Please provide additional details regarding participant consent. In the ethics statement in the Methods and online submission information, please ensure that you have specified (1) whether consent was informed and (2) what type you obtained (for instance, written or verbal, and if verbal, how it was documented and witnessed). If your study included minors, state whether you obtained consent from parents or guardians. If the need for consent was waived by the ethics committee, please include this information.

3. If you are reporting a retrospective study of medical records or archived samples, please ensure that you have discussed whether all data were fully anonymized before you accessed them and/or whether the IRB or ethics committee waived the requirement for informed consent. If patients provided informed written consent to have data from their medical records used in research, please include this information.

4. Thank you for stating the following financial disclosure:

“This study was supported by NIH-NIA grants R01AG058817 (PI:WC), R01AG022983 (PI:WC), R01AG032289 (PI: JHK), R01AG048234 (PI: JHK), UCSF ADRC P30AG062422 (PI: GDR), R01AG072475 (PI: KBC), UF1NS100608 (PI: JHK), K23AG058752 (PI: KBC), K24AG083117 (PI: WC), and Alzheimer’s Association Research Grants AARG-20-683875 (PI: KBC), AARF-23-1145318 (PI: RS). Our work was also supported by a grant from the Larry L. Hillblom Foundation (2018-A-006-NET; PI: JHK).”

Please state what role the funders took in the study.  If the funders had no role, please state: "The funders had no role in study design, data collection and analysis, decision to publish, or preparation of the manuscript."

If this statement is not correct you must amend it as needed.

Please include this amended Role of Funder statement in your cover letter; we will change the online submission form on your behalf.

5. Please expand the acronym “NIH-NIA” (as indicated in your financial disclosure) so that it states the name of your funders in full.

This information should be included in your cover letter; we will change the online submission form on your behalf.

6. Please note that funding information should not appear in any section or other areas of your manuscript. We will only publish funding information present in the Funding Statement section of the online submission form. Please remove any funding-related text from the manuscript.

7. When completing the data availability statement of the submission form, you indicated that you will make your data available on acceptance. We strongly recommend all authors decide on a data sharing plan before acceptance, as the process can be lengthy and hold up publication timelines. Please note that, though access restrictions are acceptable now, your entire data will need to be made freely accessible if your manuscript is accepted for publication. This policy applies to all data except where public deposition would breach compliance with the protocol approved by your research ethics board. If you are unable to adhere to our open data policy, please kindly revise your statement to explain your reasoning and we will seek the editor’s input on an exemption. Please be assured that, once you have provided your new statement, the assessment of your exemption will not hold up the peer review process.

8. If the reviewer comments include a recommendation to cite specific previously published works, please review and evaluate these publications to determine whether they are relevant and should be cited. There is no requirement to cite these works unless the editor has indicated otherwise.

Reviewers' comments:

Reviewer’s Responses to Questions

**Comments to the Author**

1. Is the manuscript technically sound, and do the data support the conclusions?

Reviewer #1: Partly

Reviewer #2: Partly

**********

2. Has the statistical analysis been performed appropriately and rigorously?

Reviewer #1: Yes

Reviewer #2: Yes

**********

3. Have the authors made all data underlying the findings in their manuscript fully available?

The PLOS Data policy

Reviewer #1: Yes

Reviewer #2: Yes

**********

4. Is the manuscript presented in an intelligible fashion and written in standard English?

Reviewer #1: Yes

Reviewer #2: Yes

**********

Reviewer #1: General Comments

Major Weaknesses:

Critical Temporal Confounding: The study methodology permits a time gap of up to three years between behavioral testing and neuropsychological assessment, and up to four years between behavioral testing and MRI acquisition. In a cohort of older adults ranging up to 95 years of age, white matter microstructure and cognitive processing speeds are subject to substantial nonlinear degeneration over a 48-month period. Correlating real-time decision-making parameters with historical neural architecture severely compromises the validity of the structure-function models.

Unjustified Omission of Statistical Corrections: The decision to forego multiple comparison corrections across three tasks and three distinct Drift Diffusion Model (DDM) parameters (resulting in numerous independent and non-independent tests) is statistically invalid. The primary structural finding—that global fractional anisotropy (FA) mediates age and drift rate in emotion recognition—relies on an uncorrected, marginal alpha ($p=0.044$). The authors explicitly acknowledge this finding would not survive correction, rendering the mechanistic conclusions fragile and highly susceptible to Type I error.

Methodological Heterogeneity in Response Coding: The delay discounting task boundary conditions are modeled dynamically based on a pre-estimated temporal preference parameter ($k$) to denote "consistent/inconsistent" choices. In contrast, the emotion recognition and memory tasks utilize objective "correct/error" boundaries. This fundamental difference in decision architecture confounds cross-task comparisons; a failure to accumulate evidence for a subjective preference is not psychologically equivalent to a failure to accumulate evidence for objective facial or lexical recognition.

Environmental Variance in Slow-RT Paradigms: Conducting unconstrained, home-based online data collection for millisecond-sensitive reaction time modeling introduces severe environmental noise. The resultant non-decision times ($t_0 > 2$ seconds) are abnormally long for DDM frameworks. While the authors cite precedent for slow-RT models, they fail to adequately control for the hardware, connectivity, and environmental distraction variances inherent to remote assessment in a geriatric cohort.

Minor Weaknesses:

Cross-Sectional Mischaracterization: The manuscript frequently employs longitudinal terminology (e.g., "trajectory", "changes") despite relying entirely on cross-sectional data.

Homogeneous Cohort: The sample is demographically constrained, being 85-87% White and highly educated (mean >17 years), which limits the generalizability of the findings to broader aging populations.

Specific Comments

Page 8, Lines 26-27: The use of the term "trajectory" is methodologically inaccurate for a cross-sectional design. Replace with "cross-sectional age-related differences."

Page 13, Lines 158-164: The unconstrained, home-based online data collection introduces critical unmeasured chronobiological and environmental confounders. Variables such as sleep quality, time-of-day testing, and stimulant intake significantly alter cognitive processing speed and decision-making parameters, which are indistinguishable from true age-related cognitive decline in your dataset [Bouzouraa E, Dhahbi W, Ferchichi A, Geantă VA, Kunszabo MI, Chtourou H, Souissi N: Single-night sleep extension enhances morning physical and cognitive performance across time of day in physically active university students: a randomized crossover study. Life 2025, 15(8):1178] [Amor SB, Dhahbi W, Bougrine H, Bessifi M, Geantă VA, Ursu VE, Trabelsi K, Souissi N: Differential Time-of-Day Effects of Caffeine Capsule and Mouth Rinse on Cognitive Performance in Adolescent Male Volleyball Athletes: A Randomized Crossover Investigation. Life 2025, 16(1):33]. Furthermore, the lack of standardized assessment controls for these temporal artifacts undermines the integrity of the algorithmic diffusion models [Dhahbi W, Dergaa I: Machine learning with temporal control designs for testing rhythmic specificity in chronobiology: A multivariate framework proposal for distinguishing genuine biological rhythms from temporal artifacts. Chronobiology International 2026:1-8] [Dhahbi W, Chamari K: The Algorithmic Athlete: A Call to Standardize Assessment of Sensor Technologies and Artificial Intelligence. International Journal of Sports Physiology and Performance 2026, 1(aop):1-2]. You must add a limitations paragraph detailing these specific confounders and their potential to skew non-decision time and drift rate.

Page 13, Lines 170-174: The linkage of behavioral data to neuropsychological data acquired up to three years prior, and neuroimaging data up to four years prior, requires rigorous statistical justification. You must include a sensitivity analysis isolating participants with concurrent data ($<6$ months) to prove the extensive time gap does not drive the null or marginal associations.

Page 15, Lines 222-223: Excluding participants based solely on a mean displacement threshold of >1mm is excessively liberal for diffusion tensor imaging in aging populations, where sub-millimeter head motion introduces severe artifacts in FA and MD estimations. Re-evaluate the imaging data using framewise displacement (FD) thresholds or robust motion scrubbing protocols.

Page 16, Lines 259-262: Trimming the slowest 5% of responses and applying an arbitrary hard cutoff at 350ms alters the natural skew of the RT distribution, which is the mathematical foundation of DDM estimation. Provide distributional normality checks before and after this aggressive trimming.

Page 16, Lines 266-275: The rationale provided for omitting family-wise error correction is statistically inadequate. Non-independence of tasks does not justify evaluating an uncorrected threshold across multiple parameters. Apply False Discovery Rate (FDR) or Bonferroni adjustments to all correlational and mediation models.

Page 17, Lines 283-290: Extracting the discounting parameter $k$ via maximum likelihood prior to executing the DDM introduces mathematical dependency. You are fitting a model onto the residuals of another model. You must justify why a hierarchical Bayesian DDM combining the hyperbolic discount function directly into the drift rate estimation was not utilized.

Page 20, Lines 371-375: The final trial counts (87 for delay discounting) are perilously low for reliable DDM parameter estimation, particularly when inter-trial variability parameters are included. Report the parameter recovery and test-retest reliability metrics for this specific trial density.

Page 23, Lines 450-451: The unstandardized indirect effect of global FA (-1.23e-3, $p=0.044$) is marginally significant. Given the text acknowledges this fails multiple comparison correction, this specific finding must be downgraded in the abstract and discussion from a definitive mechanism to a strictly exploratory, underpowered observation.

Page 26, Lines 537-539: The assertion that this study effectively evaluates the "oldest old" is overstated. With group sizes of $N=19$, $N=31$, and $N=29$ for participants >85 years old across the three tasks, the study lacks the statistical power to reliably model DDM variance in this specific age stratum.

Page 28, Lines 616-619: The discussion of cross-sectional mediation limitations is placed too late in the manuscript and downplays the severity of the mathematical bias. Causal chain language (e.g., "impact", "underlying") must be systematically removed from the entire manuscript.

Reviewer #2: The manuscript addresses an important and timely question regarding the mechanisms underlying age-related slowing in cognition by applying drift diffusion modelling (DDM) across a relatively large cohort of older adults spanning middle age to the oldest-old. The study has several notable strengths, including preregistration, inclusion of participants over 85 years of age, explicit model-fit evaluation, sensitivity analyses, and the attempt to combine computational modelling with white matter measures. The use of age as a continuous variable rather than relying solely on young-versus-old group comparisons is also commendable and represents an important contribution to the aging literature.

At the same time, the manuscript raises several conceptual and methodological concerns that limit the strength of the conclusions currently drawn. In particular, the interpretation of drift rate as a relatively unitary measure of “cognitive processing speed” across highly heterogeneous tasks appears overly strong. The manuscript would benefit substantially from a more cautious and theoretically constrained interpretation of the DDM parameters, clearer justification of task selection, more detailed task descriptions, and a more rigorous treatment of several statistical and methodological issues.

Overall, I believe the study has the potential to make a valuable contribution, but the manuscript would benefit from substantial revision before the conclusions can be considered fully supported.

Major Issues

1. Overgeneralized interpretation of drift rate across fundamentally different tasks

A central concern is the interpretation of drift rate as a relatively common measure of “cognitive processing speed” across the three experimental paradigms. While drift rate may often reflect evidence accumulation efficiency, its psychological meaning is highly task-dependent.

The three tasks used in this study appear to involve substantially different latent cognitive processes:

• delay discounting: value-based preference decisions / decision conflict / impulsivity,

• emotion recognition: perceptual-social discrimination,

• memory task: episodic retrieval / familiarity-based recognition.

As a result, it is unclear whether drift rate estimates across these tasks reflect the same underlying construct. This concern is particularly strong for the delay discounting paradigm, where responses are coded as “consistent” or “inconsistent” with each participant’s estimated preference function rather than objectively correct or incorrect. In this context, drift rate may reflect preference consistency or decision certainty rather than cognitive processing speed per se.

The manuscript would benefit from a clearer theoretical framework explaining:

• why these three tasks were selected,

• what common mechanism is hypothesized across them,

• and whether the authors truly intend drift rate to be interpreted comparably across all paradigms.

At present, the manuscript sometimes reads as though all three tasks are interchangeable “processing speed tasks,” which is difficult to justify conceptually.

2. Claims regarding “task specificity” are stronger than warranted by the data

The manuscript repeatedly suggests that age-related slowing in drift rate is task-specific because significant associations were found in the emotion recognition and memory tasks but not in the delay discounting task.

However, the nonsignificant association in the delay discounting task was reportedly in the same direction as the significant effects in the other tasks. The manuscript correctly acknowledges that “significant versus nonsignificant” does not demonstrate a true difference between effects, but this important caveat is not sufficiently integrated into the overall interpretation.

No formal statistical comparison between correlations/effects across tasks appears to have been performed. Therefore, the data do not yet demonstrate genuine task specificity.

The conclusions should therefore be substantially softened, or direct statistical comparisons between task effects should be added.

3. Insufficient detail and reproducibility of task descriptions

The experimental task descriptions lack sufficient methodological detail for replication and, more importantly, make it difficult to interpret the DDM parameters psychologically.

Several critical details are missing or insufficiently specified, including:

• exact trial timing,

• fixation/intertrial intervals,

• response windows,

• instructions,

• practice procedures,

• stimulus balancing/randomization,

• staircase parameters,

• and difficulty progression rules.

This issue is particularly important because DDM parameter interpretation depends heavily on task architecture.

Delay discounting task

The description raises conceptual concerns regarding what participants were actually required to compute during the task. If participants rapidly adopt a stable strategy (e.g., always selecting delayed larger rewards), then the task may become more reflective of preference policy execution than ongoing evidence accumulation.

Emotion recognition task

The adaptive staircase procedure is insufficiently described. Important information is missing regarding:

• starting difficulty,

• step sizes,

• reversal criteria,

• separate staircases by emotion,

• and stopping rules.

More importantly, because task difficulty was adaptively adjusted per participant, it becomes unclear how directly comparable drift rate estimates are across individuals. Drift rate is strongly influenced by stimulus discriminability/task difficulty, and participants may effectively have completed different tasks.

Memory task

The memory task adaptively manipulated:

• encoding duration,

• retention interval,

• and memory load simultaneously.

This creates major interpretational ambiguity regarding which aspect of task difficulty contributed to drift rate differences. It is also unclear how these varying task parameters were accounted for in the analyses.

The manuscript should more explicitly discuss how adaptive task procedures influence between-subject interpretation of DDM parameters.

4. Temporal mismatch between behavioural, neuropsychological, and MRI measures

The study allows up to:

• 3 years between behavioural and neuropsychological measures,

• and 4 years between behavioural and MRI acquisition.

Given the advanced age range of the cohort (including participants over 85 years old), this is potentially problematic. White matter integrity, processing speed, and cognitive performance can change substantially over these intervals in older populations.

Although the TBSS analyses included time difference covariates, it is unclear whether the mediation analyses adequately accounted for these temporal discrepancies.

This issue substantially weakens mechanistic interpretations linking white matter changes to DDM parameters.

5. Mediation analyses appear overinterpreted

The mediation findings should be interpreted more cautiously.

The significant mediation effect:

• was modest,

• would not survive correction for multiple comparisons according to the authors themselves,

• was based on cross-sectional data,

• and involved temporally mismatched measures.

Furthermore, the mediation used global FA averages, which substantially reduces anatomical specificity. Meanwhile, the voxelwise TBSS analyses did not show strong converging associations between regional white matter measures and drift rate parameters.

As a result, the manuscript should avoid mechanistic or causal interpretations suggesting that white matter alterations explain age-related slowing in evidence accumulation.

6. Statistical analyses are under-modelled relative to the complexity of the questions

The main behavioural analyses rely primarily on simple bivariate Spearman correlations.

Given:

• the broad age range,

• unequal age-group sizes,

• highly educated sample,

• online testing environment,

• adaptive task difficulty,

• and temporally mismatched multimodal data,

this analytic strategy appears overly simplistic.

Particularly concerning is the complete absence of nonlinear analyses. The manuscript repeatedly discusses aging trajectories and lifespan changes, yet all analyses appear effectively monotonic/linear despite the high likelihood of nonlinear aging effects in oldest-old populations.

The manuscript would benefit from:

• regression-based analyses,

• covariate adjustment,

• nonlinear modelling,

• or at minimum stronger justification for the analytic simplicity.

7. Multiple comparison concerns remain substantial

The manuscript explicitly states that no multiple-comparison correction was applied.

Although the preregistration and primary/ancillary distinction are appreciated, the study nevertheless includes a large number of:

• correlations,

• DDM parameters,

• tasks,

• mediation analyses,

• and exploratory neuroimaging analyses.

This issue is especially relevant given that some key findings appear statistically fragile.

8. Uneven age distribution limits interpretation of “continuous aging trajectories”

Although the continuous-age approach is preferable to simple young-versus-old comparisons, the sampled age distribution is markedly uneven.

Most participants fall within the 75–84 age range, while middle-aged and oldest-old groups are substantially smaller. This limits the extent to which the analyses can characterize continuous age-related trajectories across the entire sampled range.

Additional descriptive presentation of DDM parameters by age group would help readers evaluate:

• the stability of effects,

• possible nonlinearities,

• and whether findings are disproportionately driven by specific age ranges.

Minor Issues

1. Definition of “neurologically healthy” is insufficiently specified

The manuscript states that participants were verified as neurologically normal through multidisciplinary assessment. However, no operational criteria are provided.

The manuscript should clarify:

• neuropsychological cutoffs,

• exclusion criteria,

• whether MCI/subclinical impairment was excluded,

• and how “healthy aging” was defined.

This is particularly important given the advanced age of the cohort.

2. Rationale for young-adult normalization of the RT-based composite is unclear

The RT-based speed composite was normalized using a healthy young-adult reference sample.

Because the present study does not include young adults and focuses only on older participants, the rationale for this normalization is unclear. It would help to clarify whether the normalization was intended merely for scaling purposes or whether the resulting measure should be interpreted as age-relative impairment.

3. Trial trimming procedure deserves further discussion

The exclusion of the slowest 5% of responses was justified by concerns regarding distraction during online testing. While understandable, extremely slow responses may also reflect genuine aging-related variability rather than artifacts.

The potential influence of trimming on DDM parameter estimation should be discussed more explicitly.

Strengths

Despite the concerns above, the manuscript has several important strengths:

• preregistration,

• inclusion of oldest-old adults,

• use of continuous age rather than dichotomous grouping,

• explicit model-fit assessment,

• sensitivity analyses,

• computational modelling approach,

• and integration of behavioural and neuroimaging measures.

The model-fit evaluation procedure, including Monte Carlo simulations and graphical diagnostics, is particularly careful and represents a methodological strength of the study.

Overall Evaluation

This manuscript addresses an important topic and includes several valuable methodological features. However, the current interpretation of the findings is, in my view, more conceptually unified and mechanistically specific than the data presently justify.

The manuscript would become substantially stronger if the authors:

• adopted a more cautious interpretation of drift rate,

• more explicitly acknowledged task heterogeneity,

• clarified the theoretical role of the three paradigms,

• reduced mechanistic claims,

• and more carefully addressed the limitations introduced by adaptive task procedures and temporally mismatched multimodal measures.

At present, the data may be better suited to supporting a nuanced account of task-dependent aging effects on DDM-derived parameters than a more general account of age-related slowing of information accumulation.

**********

what does this mean?). If published, this will include your full peer review and any attached files.

If you choose “no”, your identity will remain anonymous but your review may still be made public.

**Do you want your identity to be public for this peer review?**  For information about this choice, including consent withdrawal, please see our Privacy Policy

Reviewer #1: **Yes:**  Wissem Dhahbi

Reviewer #2: No

**********

[NOTE: If reviewer comments were submitted as an attachment file, they will be attached to this email and accessible via the submission site. Please log into your account, locate the manuscript record, and check for the action link "View Attachments". If this link does not appear, there are no attachment files.]

To ensure your figures meet our technical requirements, please review our figure guidelines: https://journals.plos.org/plosone/s/figures

You may also use PLOS’s free figure tool, NAAS, to help you prepare publication quality figures: https://journals.plos.org/plosone/s/figures#loc-tools-for-figure-preparation.

NAAS will assess whether your figures meet our technical requirements by comparing each figure against our figure specifications.

# Author comment: Author response to Decision Letter 1

**DOI:** 10.1371/journal.pone.0361899.r002

21 Jul 2026

Reviewer #1: General Comments

Major Weaknesses:

Critical Temporal Confounding: The study methodology permits a time gap of up to three years between behavioral testing and neuropsychological assessment, and up to four years between behavioral testing and MRI acquisition. In a cohort of older adults ranging up to 95 years of age, white matter microstructure and cognitive processing speeds are subject to substantial nonlinear degeneration over a 48-month period. Correlating real-time decision-making parameters with historical neural architecture severely compromises the validity of the structure-function models.

Unjustified Omission of Statistical Corrections: The decision to forego multiple comparison corrections across three tasks and three distinct Drift Diffusion Model (DDM) parameters (resulting in numerous independent and non-independent tests) is statistically invalid. The primary structural finding—that global fractional anisotropy (FA) mediates age and drift rate in emotion recognition—relies on an uncorrected, marginal alpha ($p=0.044$). The authors explicitly acknowledge this finding would not survive correction, rendering the mechanistic conclusions fragile and highly susceptible to Type I error.

Methodological Heterogeneity in Response Coding: The delay discounting task boundary conditions are modeled dynamically based on a pre-estimated temporal preference parameter ($k$) to denote "consistent/inconsistent" choices. In contrast, the emotion recognition and memory tasks utilize objective "correct/error" boundaries. This fundamental difference in decision architecture confounds cross-task comparisons; a failure to accumulate evidence for a subjective preference is not psychologically equivalent to a failure to accumulate evidence for objective facial or lexical recognition.

We thank the reviewer for this point and have sought to clarify the relationship of this task to the central aim of the project. The drift diffusion model has been used by many investigators to model a wide and heterogenous range of cognitive operations, not limited to perceptual tasks (such as our emotion recognition task), but also including tasks requiring the internal maintenance and manipulation of representations (as in our memory task) and tasks of value-based decision-making (as in our delay discounting task). In addition, age-related slowing is observed across a similarly wide and heterogenous range of cognitive operations, including value-based decision-making; and several investigators have sought to mechanistically decompose such slowing in terms of changes in parameters of the DDM. Two contributions of our study to the literature are (1) modeling of age as a continuous parameter within a common cohort (rather than relying on group-level comparisons across young and old cohorts), and (2) estimation of DDM parameters across heterogeneous operations within the same cohort and with an overlapping set of demographic, neuropsychological, and neuroimaging measures. In the context of value-based decision-making, while these do not have an external or objective standard of “correctness” as in perceptual or memory decisions, one accepted approach is to analogize correct choices to those that maximize a subjective value/utility function (as within our task, “consistent” choices are those that maximize the subjective present value of a participant’s choice given their estimated k parameter; for related interpretations of accuracy or correctness in value-based decisions using the DDM see Milosavljevic et al, Judgment and Decision Making 2010, Copeland et al, Cogent Pyschology 2022.)

Then, to clarify the purposes of our study: our interest in testing predictions about changes in DDM parameters with aging across heterogeneous cognitive operations does not depend on the claim that these operations are psychologically equivalent. Instead, it stems from the recognition (1) that age-related slowing is observed across a heterogeneous range of cognitive operations, including value-based decision-making, (2) that the DDM has been productively used to model putative cognitive processes underlying s similarly heterogeneous range of cognitive operations, including value-based decision-making, and (3) that investigators have begun to investigate whether age-related cognitive slowing can be mechanistically decomposed using the DDM. In our view, the heterogeneity of the tasks included in our analysis is representative of the range of cognitive operations across which age-related cognitive slowing is observed and across which the DDM has been productively applied. In our preregistered exploratory analysis we evaluated whether changes in DDM parameters with aging across heterogeneous cognitive operations may reflect a common underlying mechanism (despite the psychological heterogeneity of these operations), although these analyses were generally inconclusive.

We have now made some changes to the manuscript:

- Page 2 lines 40-41: ‘Age-related slowing is observed across a similarly wide and heterogenous range of cognitive operations.’

- Page 12 lines 337-339: ‘In the context of value-based decision-making tasks, while these do not have an external or objective standard of “correctness” as in perceptual or memory decisions, a widely used approach is to analogize correct choices to those that maximize a subjective value/utility function.’

- Page 24 lines 671-675: ‘Two contributions of our study to the literature are (1) modeling of age as a continuous parameter within a common cohort (rather than relying on group-level comparisons across young and old cohorts), and (2) estimation of DDM parameters across heterogeneous operations within the same cohort and with an overlapping set of demographic, neuropsychological, and neuroimaging measures.’

- Page 26 lines 722-728: ‘However, our interest in testing predictions about changes in DDM parameters with aging across heterogeneous cognitive operations does not depend on the claim that these operations are psychologically equivalent. Instead, it stems from the recognition (1) that age-related slowing is observed across a heterogeneous range of cognitive operations (2) that the DDM has been productively used to model putative cognitive processes underlying a similarly heterogeneous range of cognitive operations, and (3) that investigators have begun to investigate whether age-related cognitive slowing can be mechanistically decomposed using the DDM.’

Environmental Variance in Slow-RT Paradigms: Conducting unconstrained, home-based online data collection for millisecond-sensitive reaction time modeling introduces severe environmental noise. The resultant non-decision times ($t_0 > 2$ seconds) are abnormally long for DDM frameworks. While the authors cite precedent for slow-RT models, they fail to adequately control for the hardware, connectivity, and environmental distraction variances inherent to remote assessment in a geriatric cohort.

We thank the reviewer for this comment. We agree that unsupervised home-based testing may introduce additional sources of variability related to participants' environment, hardware, or temporary distractions. This was one of the motivations for excluding the slowest 5% of responses prior to DDM estimation, as these responses were considered more likely to reflect lapses of attention or environmental interruptions rather than the cognitive processes of interest. As requested by the reviewers, we have now included histograms of the reaction time distributions before and after trimming, together with a sensitivity analysis including all responses, which demonstrated that the main findings follow the same pattern.

Regarding the relatively long non-decision times, these values are expected given the characteristics of our experimental paradigms and are consistent with previous applications of the DDM to tasks with relatively slow response times in older adults. Non-decision time in this context reflects not only perceptual encoding and motor execution but also task-specific processes occurring outside evidence accumulation, and therefore should not be directly compared with values obtained from simple laboratory reaction-time paradigms.

Finally, we acknowledge that the home-based testing environment represents a limitation of the present study. We have expanded the Discussion accordingly.

Minor Weaknesses:

Cross-Sectional Mischaracterization: The manuscript frequently employs longitudinal terminology (e.g., "trajectory", "changes") despite relying entirely on cross-sectional data.

Homogeneous Cohort: The sample is demographically constrained, being 85-87% White and highly educated (mean >17 years), which limits the generalizability of the findings to broader aging populations.

We thank the reviewer for this observation. We agree that the demographic characteristics of our cohort, which is predominantly White and highly educated, may limit the generalizability of our findings to more diverse aging populations. We have now added this as a limitation to the Discussion:

- Page 27 lines 758-760: ‘Moreover, our cohort was predominantly White and highly educated, which may limit the generalizability of our findings to more socioeconomically, educationally, and ethnically diverse aging populations.’

Specific Comments

Page 8, Lines 26-27: The use of the term "trajectory" is methodologically inaccurate for a cross-sectional design. Replace with "cross-sectional age-related differences."

The term has been replaced throughout the manuscript:

- Page 1 line 21: ‘…was to evaluate the cross-sectional age-related differences of processes…’

- Page 4 line 103: ‘…cross-sectional age-related differences of drift rate…’

- Page 27 line 754: ‘…and to be able to look at age-related associations in DDM parameters…’

- Page 27 line 757: ‘…which still limits our ability to characterize these associations in middle age and in later older adulthood.’

Page 13, Lines 158-164: The unconstrained, home-based online data collection introduces critical unmeasured chronobiological and environmental confounders. Variables such as sleep quality, time-of-day testing, and stimulant intake significantly alter cognitive processing speed and decision-making parameters, which are indistinguishable from true age-related cognitive decline in your dataset [Bouzouraa E, Dhahbi W, Ferchichi A, Geantă VA, Kunszabo MI, Chtourou H, Souissi N: Single-night sleep extension enhances morning physical and cognitive performance across time of day in physically active university students: a randomized crossover study. Life 2025, 15(8):1178] [Amor SB, Dhahbi W, Bougrine H, Bessifi M, Geantă VA, Ursu VE, Trabelsi K, Souissi N: Differential Time-of-Day Effects of Caffeine Capsule and Mouth Rinse on Cognitive Performance in Adolescent Male Volleyball Athletes: A Randomized Crossover Investigation. Life 2025, 16(1):33]. Furthermore, the lack of standardized assessment controls for these temporal artifacts undermines the integrity of the algorithmic diffusion models [Dhahbi W, Dergaa I: Machine learning with temporal control designs for testing rhythmic specificity in chronobiology: A multivariate framework proposal for distinguishing genuine biological rhythms from temporal artifacts. Chronobiology International 2026:1-8] [Dhahbi W, Chamari K: The Algorithmic Athlete: A Call to Standardize Assessment of Sensor Technologies and Artificial Intelligence. International Journal of Sports Physiology and Performance 2026, 1(aop):1-2]. You must add a limitations paragraph detailing these specific confounders and their potential to skew non-decision time and drift rate.

We thank the reviewer for raising this concern. The following lines have now been added to the limitations paragraph to address this concern:

- Page 26 lines 711-716: ‘Another constraint inherent to our at-home testing design is the inability to control for variables such as sleep quality, time-of-day testing, and stimulant intake, that have been shown to have an influence on cognitive performance [56,57,58], thus having the potential to skew non-decision time and drift rate in our tasks, two of the DDM parameters analyzed in this study. Further studies should directly control such variables in order to exclude possible influences on the results obtained.’

Page 13, Lines 170-174: The linkage of behavioral data to neuropsychological data acquired up to three years prior, and neuroimaging data up to four years prior, requires rigorous statistical justification. You must include a sensitivity analysis isolating participants with concurrent data ($<6$ months) to prove the extensive time gap does not drive the null or marginal associations.

We thank the reviewer for this important comment. We acknowledge that the interval between online task completion and the linked neuropsychological and neuroimaging assessments is a limitation of this study and reflects the longitudinal design of the cohort, with additional delays introduced by the COVID-19 pandemic. We note that this concern applies only to analyses involving the RT-based speed composite (hypothesis ii) and neuroimaging measures (exploratory hypothesis), as age and online task performance were assessed at the same time.

A sensitivity analysis restricted to assessments acquired within 6 months would retain only a small sample across tasks (N = 19 for the delay discounting task and N = 36 for the emotion recognition and memory tasks). Instead, we performed a sensitivity analysis restricted to participants for whom a subsequent longitudinal evaluation was available following completion of the online tasks, at which they were confirmed to remain cognitively and neurologically healthy. We added the following sentences to the Methods and Results, respectively:

- Page 14 lines 417-421: ‘.Given the time gap between online task evaluation and RT-based speed composite and neuroimaging acquisitions, sensitivity analysis for hypothesis ii and exploratory hypothesis iii was performed including only participants for whom a subsequent longitudinal evaluation was available following completion of the online tasks, at which they were confirmed to remain cognitively and neurologically healthy.’

- Page 19 lines 528-533: ‘Table A3 shows results for the sensitivity analysis, including only participants for whom a subsequent longitudinal evaluation was available following completion of the online tasks, at which they were confirmed to remain cognitively and neurologically healthy. Given the reduced sample size, confidence intervals in this sensitivity analysis were wider and comparisons were not statistically significant, although the overall pattern of associations was generally consistent with the primary analyses.’

- Page 20 lines 550-554: ‘For the sensitivity analysis including only participants for whom a subsequent longitudinal evaluation was available following completion of the online tasks, at which they were confirmed to remain cognitively and neurologically healthy, the sample size was reduced to N=77 and bootstrapped unstandardized indirect effect was -3.3e-3 (p = 0.006).’

Page 15, Lines 222-223: Excluding participants based solely on a mean displacement threshold of >1mm is excessively liberal for diffusion tensor imaging in aging populations, where sub-millimeter head motion introduces severe artifacts in FA and MD estimations. Re-evaluate the imaging data using framewise displacement (FD) thresholds or robust motion scrubbing protocols.

We thank the reviewer for raising this important point. We agree that head motion is an important potential confound in diffusion MRI studies, particularly in older adults.

However, our diffusion MRI data were processed using FSL’s eddy, which jointly performs motion correction and eddy-current correction and incorporates slice-level detection and replacement of signal affected by motion during diffusion encoding. As described in the Methods, all MR images were visually inspected for motion-related artifacts (page 8 lines 218-219), all preprocessed images were visually inspected for registration errors, and FSL QUAD/SQUAD quality-control reports were reviewed prior to participant exclusion (page 8 lines 236-237). The FSL QUAD/SQUAD quality-control framework provides diffusion-specific motion metrics, and participants exceeding mean displacement > 1mm were excluded. In addition, mean displacement was not associated with age in the retained sample (Spearman’s ρ = 0.043, p = 0.576) (as shown in Appendix Figure A9), reducing the likelihood that age-related associations were driven by systematic differences in head motion. We now report descriptive motion metrics for the retained sample:

- Page 8 lines 239-240: ‘Among the retained participants mean displacement was 0.53mm ± 0.37mm.’

Page 16, Lines 259-262: Trimming the slowest 5% of responses and applying an arbitrary hard cutoff at 350ms alters the natural skew of the RT distribution, which is the mathematical foundation of DDM estimation. Provide distributional normality checks before and after this aggressive trimming.

We thank the reviewer for this comment. Regarding the 350 ms cutoff, this threshold was selected based on previous DDM studies in older adult cohorts. Furthermore, in our cohort no participant had reaction times faster than 350 ms; therefore, no trials were excluded by this criterion, as stated on page 15 lines 448–449.

Regarding the exclusion of the slowest 5% of responses, we agree that this trimming alters the right tail of the RT distribution. However, its purpose was to reduce the influence of lapses and environmental distractions, which are of particular concern given that the tasks were completed in an unconstrained home environment. We have now included histograms of the RT distributions before and after trimming (Appendix Figure A1). We added this sentence to the manuscript ‘Visual inspection confirmed that the characteristic positively skewed RT distributions were preserved after the exclusion of the 5% slowest trials (Appendix Figure A1).’ (page 15 lines 449-450).

Most importantly, we performed a sensitivity analysis including all responses (Appendix Tables A1 and A2). The results follow the same pattern, showing that our conclusions are not driven by the exclusion of the slowest 5% of responses.

Page 16, Lines 266-275: The rationale provided for omitting family-wise error correction is statistically inadequate. Non-independence of tasks does not justify evaluating an uncorrected threshold across multiple parameters. Apply False Discovery Rate (FDR) or Bonferroni adjustments to all correlational and mediation models.

We agree with the reviewer that non-independence among tasks does not, by itself, justify foregoing correction for multiple comparisons. Therefore, for the primary correlations, p-values were adjusted for multiple comparisons across 6 tests of the preregistered hypotheses (associations between age and drift rate for the three tasks, association between RT-based composite measure and drift rate for the three tasks) by applying Benjamini–Hochberg FDR correction. For ancillary analyses (involving boundary separation and non-decision time), effect sizes are now presented with 95% confidence intervals but without associated p-values to emphasize estimation rather than formal hypothesis testing. P-values adjusted for multiple comparisons across 10 tests (the 6 associations of the primary hypothesis and the 4 mediation models performed) are now reported for the mediation analysis.

Tables 3 and 4 have been updated to reflect these changes and the manuscript has been updated accordingly. The following changes were made to the manuscript:

- Page 5 lines 142-143: ‘Preregistered ancillary analyses were also conducted to examine associations between age and boundary separation and non-decision time.’

- Page 5 lines 145-146: ‘Preregistered ancillary analyses were also conducted to examine associations between RT-based measures and boundary separation and non-decision time.’

- Pages 11-12 lines 331-335: ‘Because our preregistered primary hypothesis was evaluated across three tasks, p-values for the drift rate analyses were adjusted for multiple comparisons using the Benjamini–Hochberg false discovery rate (FDR) procedure. Associations involving boundary separation and non-decision time were considered ancillary analyses and are reported as effect sizes with 95% confidence intervals.’

-Page 14 lines 395-401: ‘Because each primary hypothesis was evaluated across three tasks, p-values for 6 associations were adjusted for multiple comparisons using the Benjamini–Hochberg false discovery rate (FDR) procedure. Additionally, we examined the association between age and the other two diffusion model parameters (boundary-separation and non-decision time) and between the RT-based speed composite and these two parameters, as prespecified ancillary analyses. These ancillary analyses are reported as effect sizes with 95% confidence intervals.’

- Page 14 lines 409-412: ‘P-values for these exploratory analysis were adjusted for 10 comparisons (the 6 associations of the primary hypothesis and the 4 mediation models performed) using the Benjamini–Hochberg false discovery rate (FDR) procedure.’

- Page 17 lines 497-505: ‘Age was significantly negatively associated with drift rate for both the emotion recognition (ρ = -0.19, p = 0.016) and the memory tasks (ρ = -0.18, p = 0.026) (Figure 1 and Table 3). Additionally, age was positively associated with boundary separation in the emotion recognition task (ρ = 0.16, 95% CI [0.017-0.29]), whereas little evidence of an association was observed in the other tasks. Age was also positively associated with non-decision time across all three tasks (delay discounting: ρ = 0.33, 95% CI [0.19-0.46]; emotion recognition: ρ = 0.37, 95% CI [0.24-0.49]; memory: ρ = 0.39, 95% CI [0.25-0.51]) (Table 3 and Appendix Figure A7).’

- Pages 18-19 lines 519-526: ‘The RT-based speed composite was significantly negatively associated with drift rate for both the delay discounting (ρ = -0.274, p = 0.003) and the memory tasks (ρ = -0.271, p = 0.016). In addition, a positive association was found between the RT-based speed composite and boundary separation for the delay discounting (ρ =0.272, 95% CI [0.12-0.41]) and the memory tasks (ρ = 0.223, 95% CI [0.022-0.41]), and the association between the RT-based speed composite and non-decision time was positive across all three tasks (delay discounting: ρ = 0.19, 95% CI [0.03-0.33]; emotion recognition: ρ = 0.33, 95% CI [0.15-0.49]; memory: ρ = 0.25, 95% CI [0.047-0.43]) (Table 4).’

- Pages 19-20 lines 546-548: ‘The bootstrapped unstandardized indirect effect was -1.69e-3 (uncorrected p = 0.036). However, this result did not persist when adjusted for multiple comparisons (p = 0.067).’

Please note that the original uncorrected indirect effect and p-value for the mediation analysis have been corrected from -1.23e-3 to -1.69e-3 and 0.044 to 0.036, respectively. This change reflects a transcription error identified during the revision process.

Page 17, Lines 283-290: Extracting the discounting parameter $k$ via maximum likelihood prior to executing the DDM introduces mathematical dependency. You are fitting a model onto the residuals of another model. You must justify why a hierarchical Bayesian DDM combining the hyperbolic discount function directly into the drift rate estimation was not utilized.

We agree that the delay-discounting DDM was conditional on the first-stage estimate of k. We have clarified this in the manuscript:

- Page 23 lines 618-621: ‘Finally, we used a two-stage procedure for this task, applying maximum likelihood estimation to the hyperbolic discounting model to estimate k – for posterior response coding – before fitting the DDM, making the delay discounting DDM conditional on this first stage.’

We now also acknowledge it as a limitation:

- Page 26 lines 719-722: ‘Moreover, the nature of the delay discounting task and the two-stage procedure used to fit the DDM to this task prevent an optimal comparison across tasks and results regarding this task should be interpreted with caution.’

However, k was used only to define participant-specific response coding in a task without objectively correct responses, not as a regressor or residualized predictor in the DDM. The purpose was to create response categories analogous to correct/incorrect classifications in the other tasks while maintaining a common fast-dm framework across tasks.

We agree that a joint hierarchical Bayesian model incorporating the hyperbolic value function directly into trialwise drift rate might be a more powerful approach. However, this would require a different modeling framework from fast-dm and would reduce comparability with the other two tasks. We now present our approach as a simpler two-stage approximation and note that future work should use joint estimation:

- Page 23 lines 621-623: ‘Future work using joint modeling approaches such as hierarchical Bayesian DDMs might lead to more robust conclusions [49,50].’

Page 20, Lines 371-375: The final trial counts (87 for delay discounting) are perilously low for reliable DDM parameter estimation, particularly when inter-trial variability parameters are included. Report the parameter recovery and test-retest reliability metrics for this specific trial density.

While we agree that 87 trials might be low for stable estimation of inter-trial variability parameters, in our model, inter-trial variability of non-decision time" " was included to improve characterization of the RT distribution (page 12, lines 357-358) , but its estimates were not used in any between-subject analyses or interpreted substantively. We therefore focused parameter recovery analyses on the parameters used for our analyses: drift rate, boundary separation and non-decision time. The following sentences have been added to the manuscript:

- Page 13 lines 386-389: ‘Lastly, parameter recovery was assessed by comparing the empirical DDM parameter estimates (drift rate, boundary separation and non-decision time) with those re-estimated after fitting the model to simulated datasets generated from the original parameter estimates, using Spearman correlations.’

- Page 16 lines 478-481: ‘Parameter recovery analyses further demonstrated good recovery of the estimated DDM parameters across all three tasks, with strong correlations between the original and re-estimated values for drift rate (ρ = 0.84–0.95), boundary separation (ρ = 0.93–0.98), and non-decision time (ρ = 0.96–0.97) (all p < 0.001) (Appendix Figure A4).’

Parameter recovery results are now shown in Appendix Figure A4.

Regarding test-retest reliability, we agree that this is an important property of DDM parameter estimation. However, evaluation of this property typically requires repeated administration of the same task to the same participants. Because the present study included a single administration of each task, we were not able to evaluate this property with the available data.

Page 23, Lines 450-451: The unstandardized indirect effect of global FA (-1.23e-3, $p=0.044$) is marginally significant. Given the text acknowledges this fails multiple comparison correction, this specific finding must be downgraded in the abstract and discussion from a definitive mechanism to a strictly exploratory, underpowered observation.

We thank the reviewer for this comment. We have now rephrased the sentence in the Abstract:

- Page 1 lines 24-26: ‘…and we show that this association in the emotion recognition task might be partially accounted for by global fractional anisotropy.’

We also rephrased the presentation of this result in the Discussion:

- Page 21 lines 580-581: ‘…our mediation models suggest that white matter microstructure changes have a significant indirect effect in the association between age and drift rate in the emotion recognition task.’

- Page 24 lines 661-662: ‘…variables suggests that global FA might partially account for the association between age and drift rate in the emotion recognition task.’

Finally, we added the following sentence to the Discussion:

- Page 24 lines 666-669: ‘However, given the marginally significant result we report, that did not survive multiple comparisons correction, this result should be interpreted with caution and be seen as exploratory rather than definite, needing further research to reach more precise conclusions.’

Page 26, Lines 537-539: The assertion that this study effectively evaluates the "oldest old" is overstated. With group sizes of $N=19$, $N=31$, and $N=29$ for participants >85 years old across the three tasks, the study lacks the statistical power to reliably model DDM variance in this specific age stratum.

We agree that, although our cohort spans a broad age range (53–95 years), the number of participants aged over 85 years is relatively modest, limiting our ability to characterize DDM variability specifically within the oldest-old age group. We acknowledge this limitation in the final paragraph of the Discussion (page 27, lines 753–757). Our goal was not to suggest that the present study was specifically designed to model age-related effects within the oldest-old subgroup. Rather, we aimed to highlight that our cohort extends into this age range, which has been underrepresented in previous studies using a continuous measure of age.

Page 28, Lines 616-619: The discussion of cross-sectional mediation limitations is placed too late in the manuscript and downplays the severity of the mathematical bias. Causal chain language (e.g., "impact", "underlying") must be systematically removed from the entire manuscript.

We thank the reviewer for this comment. The discussion of the limitations of the mediation analysis was placed alongside the presentation of the mediation results, as these analyses are presented last in the manuscript. Our intention was to discuss the limitations in the context of the corresponding findings, particularly as the mediation analyses were exploratory and not a primary focus of the study. Nevertheless, we agree with the reviewer’s concern regarding the interpretation of mediation analyses in cross-sectional data. We have now changed causal chain language throughout the manuscript:

- Page 3 lines 85-88: ‘…and found that the association between age and drift rate showed a significant indirect effect through white matter measures within the frontoparietal network, but the association between age and the non-decision time parameter did not show such effects.’

- Page 5 line 147: ‘…parameter this association is partially accounted for by changes in white matter microstructure.’

- Page 19 line 544: ‘Global FA showed a significant indirect effect in the association between age and drift rate…’

- Page 20 lines 548-550: ‘…nor global MD showed significant indirect effects in the associations between age and drift rate nor in the associations between age and boundary separation and non-decision time…’

- Page 21 lines 580-581: ‘…that white matter microstructure changes have a significant indirect effect in the association between age and drift rate in the emotion recognition task.’

- Page 24 lines 661-662: ‘…suggests that global FA might partially account for the association between age and drift rate…’

Reviewer #2:

The manuscript addresses an important and timely question regarding the mechanisms underlying age-related slowing in cognition by applying drift diffusion modelling (DDM) across a relatively large cohort of older adults spanning middle age to the oldest-old. The study has several notable strengths, including preregistration, inclusion of participants over 85 years of age, explicit model-fit evaluation, sensitivity analyses, and the attempt to combine computational modelling with white matter measures. The use of age as a continuous variable rather than relying solely on young-versus-old group comparisons is also commendable and represents an important contribution to the aging literature.

At the same time, the manuscript raises several conceptual and methodological concerns that limit the strength of the conclusions currently drawn. In particular, the interpretation of drift rate as a relatively unitary measure of “cognitive processing speed” across highly heterogeneous tasks appears overly strong. The manuscript would benefit substantially from a more cautious and theoretically constrained interpretation of the DDM parameters, clearer justification of task selection, more detailed task descriptions, and a more rigorous treatment of several statistical and methodological issues.

Overall, I believe the study has the potential to make a valuable contribution, but the manuscript would benefit from substantial revision before the conclusions can be considered fully supported.

Major Issues

1. Overgeneralized interpretation of drift rate across fundamentally different tasks

A central concern is the interpretation of drift rate as a relatively common measure of “cognitive processing speed” across the three experimental paradigms. While drift rate may often reflect evidence accumulation efficiency, its psychological meaning is highly task-dependent.

The three tasks used in this study appear to involve substantially different latent cognitive processes:

• delay discounting: value-based preference decisions / decision conflict / impulsivity,

• emotion recognition: perceptual-social discrimination,

• memory task: episodic retrieval / familiarity-based recognition.

As a result, it is unclear whether drift rate estimates across these tasks reflect the same underlying construct. This concern is particularly strong for the delay discounting paradigm, where responses are coded as “consistent” or “inconsistent” with each participant’s estimated preference function rather than objectively correct or incorrect. In this context, drift rate may reflect preference consistency or decision certainty rather than cognitive processing speed per se.

The manuscript would benefit from a clearer theoretical framework explaining:

• why these three tasks were selected,

• what common mechanism is hypothesized across them,

• and whether the authors truly intend drift rate to be interpreted comparably across all paradigms.

At present, the manuscript sometimes reads as though all three tasks are interchangeable “processing speed tasks,” which is difficult to justify conceptually.

We thank the reviewer for this point and have sought to clarify the relationship of this task to the central aim of the project. The drift diffusion model has been used by many investigators to model a wide and heterogenous range of cognitive operations, not limited to perceptual tasks (such as our emotion recognition task), but also including tasks requiring the internal maintenance and manipulation of representations (as in our memory task) and tasks of value-based decision-making (as in our delay discounting task). In addition, age-related slowing is observed across a similarly wide and heterogenous range of cognitive operations, including value-based decision-making; and several investigators have sought to mechanistically decompose such slowing in terms of changes in parameters of the DDM. Two contributions of our study to the literature are (1) modeling of age as a continuous parameter within a common cohort (rather than relying on group-level comparisons across young and old cohorts), and (2) estimation of DDM parameters across heterogeneous operations within the same cohort and with an overlapping set of demographic, neuropsychological, and neuroimaging measures. In the context of value-based decision-making, while these do not have an external or objective standard of “correctness” as in perceptual or memory decisions, a widely-used approach is to analogize correct choices to those that maximize a subjective value/utility function (as within our task, “consistent” choices are those that maximize the subjective present value of a participant’s choice given their estimated k parameter; for related interpretations of accuracy or correctness in value-based decisions using the DDM see Milosavljevic et al, Judgment and Decision Making 2010, Copeland et al, Cogent Pyschology 2022.)

Then, to clarify the purposes of our study: our interest in testing predictions about changes in DDM parameters with aging across heterogeneous cognitive operations does not depend on the claim that these operations are psychologically equivalent. Instead, it stems from the recognition (1) that age-related slowing is observed across a heterogeneous range of cognitive operations, including value-based decision-making, (2) that the DDM has been productively used to model putative cognitive processes underlying s similarly heterogeneous range of cognitive operations, including value-based decision-making, and (3) that investigators have begun to investigate whether age-related cognitive slowing can be mechanistically decomposed using the DDM. In our view, the heterogeneity of the tasks included in our analysis is representative of the range of cognitive operations across which age-related cognitive slowing is observed and across which the DDM has been productively applied. In our preregistered exploratory analysis we evaluated whether changes in DDM parameters with aging across heterogeneous cognitive operations may reflect a common underlying mechanism (despite the psychological heterogeneity of these operations), although these analyses were generally inconclusive.

We have now made some changes to the manuscript:

- Page 2 lines 40-41: ‘Age-related slowing is observed across a similarly wide and heterogenous range of cognitive operations.’

- Page 12 lines 337-339: ‘In the context of value-based decision-making tasks, while these do not have an external or objective standard of “correctness” as in perceptual or memory decisions, a widely used approach is to analogize correct choices to those that maximize a subjective value/utility function.’

- Page 24 lines 671-675: ‘Two contributions of our study to the literature are (1) modeling of age as a continuous parameter within a common cohort (rather than relying on group-level comparisons across young and old cohorts), and (2) estimation of DDM parameters across heterogeneous operations within the same cohort and with an overlapping set of demographic, neuropsychological, and neuroimaging measures.’

- Page 26 lines 722-728: ‘However, our interest in testing predictions about changes in DDM parameters with aging across heterogeneous cognitive operations does not depend on the claim that these operations are psychologically equivalent. Instead, it stems from the recognition (1) that age-related slowing is observed across a heterogeneous range of cognitive operations (2) that the DDM has been productively used to model putative cognitive processes underlying a similarly heterogeneous range of cognitive operations, and (3) that investigators have begun to investigate whether age-related cognitive slowing can be mechanistically decomposed using the DDM.’

2. Claims regarding “task specificity” are stronger than warranted by the data

The manuscript repeatedly suggests that age-related slowing in drift rate is task-specific because significant associations were found in the emotion recognition and memory tasks but not in the delay discounting task.

However, the nonsignificant association in the delay discounting task was reportedly in the same direction as the significant effects in the other tasks. The manuscript correctly acknowledges that “significant versus nonsignificant” does not demonstrate a true difference between effects, but this important caveat is not sufficiently integrated into the overall interpretation.

No formal statistical comparison between correlations/effects across tasks appears to have been performed. Therefore, the data do not yet demonstrate genuine task specificity.

The conclusions should therefore be substantially softened, or direct statistical comparisons between task effects should be added.

We agree that the presence of significant associations in some tasks and a nonsignificant association in another task does not, by itself, demonstrate a statistically reliable difference between tasks. Accordingly, we have revised the manuscript to avoid strong claims of task specificity and now describe the findings more cautiously. The following changes were made:

- Page 1 lines 26-28: ‘Our results suggest a slowing of information processing with age that may be more evident in some cognitive domains than others, although formal comparisons between tasks were not conducted.’

- Page 21 lines 585-586: ‘… suggesting that declines in drift rate with age (reflecting a slowing of information accumulation) may be more apparent in some cognitive domains than others.’

- Page 27 lines 762-763: ‘…that the strength of this association may vary across cognitive domains…’

3. Insufficient detail and reproducibility of task descriptions

The experimental task descriptions lack sufficient methodological detail for replication and, more importantly, make it difficult to interpret the DDM parameters psychologically.

Several critical details are missing or insufficiently specified, including:

• exact trial timing,

• fixation/intertrial intervals,

• response windows,

• instructions,

• practice procedures,

• stimulus balancing/randomization,

• staircase parameters,

• and difficulty progression rules.

This issue is particularly important because DDM parameter interpretation depends heavily on task architecture.

Delay discounting task

The description raises conceptual concerns regarding what participants were actually required to compute during the task. If participants rapidly adopt a stable strategy (e.g., always selecting delayed larger rewards), then the task may become more reflective of preference policy execution than ongoing evidence accumulation.

Emotion recognition task

The adaptive staircase procedure is insufficiently described. Important information is missing regarding:

• starting difficulty,

• step sizes,

• reversal criteria,

• separate staircases by emotion,

• and stopping rules.

More importantly, because task difficulty was adaptively adjusted per participant, it becomes unclear how directly comparable drift rate estimates are across individuals. Drift rate is strongly influenced by stimulus discriminability/task difficulty, and participants may effectively have completed different tasks.

Memory task

The memory task adaptively manipulated:

• encoding duration,

• retention interval,

• and memory load simultaneously.

This creates major interpretational ambiguity regarding which aspect of task difficulty contributed to drift rate differences. It is also unclear how these varying task parameters were accounted for in the analyses.

The manuscript should more explicitly discuss how adaptive task procedures influence between-subject interpretation of DDM parameters.

We thank the reviewer for raising this concern. We agree that adaptive task procedures deserve careful consideration when interpreting drift rate estimates. The adaptive procedures implemented in the emotion recognition and memory tasks were specifically designed to maintain participant performance within a comparable accuracy range (approximately 66–70%), thereby reducing floor and ceiling effects and allowing participants with different abilities to perform the tasks at a comparable level of difficulty. Consequently, although individual participants were presented with different stimulus difficulties, the adaptive procedures produced similar overall task difficulty rather than created fundamentally different tasks. We have now added more details regarding each task structure.

Regarding the delay discounting task:

- Page 9 lines 244-247: ‘Within trials, three parameters were varied independently: percent penalty (the relative difference in magnitudes between the smaller immediate and larger delayed reward), delay length (time required to wait for the delayed reward), and absolute reward magnitudes (expressed in terms of the delayed reward magnitude).’

- Page 9 lines 250-253: ‘As a control condition to ensure task comprehension, 12 trials were interspersed throughout the task either asking participants to correctly indicate which reward would be paid out sooner, or asking participants to correctly indicate which reward was the larger reward.’

- Page 9 lines 254-263: ‘Trial order was fixed for every participant, there was no constraint of time to answer and no fixation between trials (each trial started as soon as the participant answered the previous trial). Instructions to the participants were as follows: ‘We are interested in how people make decisions about money now and in the future. As a token of our appreciation, you will receive an Amazon gift card worth at least $5. We will ask you 104 questions about rewards that you could receive, either today or in the future. There are three types of questions... 'Which reward is paid SOONER?' 'Which reward is LARGER?' 'Which reward do you WANT?' Most of the questions will be about which reward you WANT. At the end, we will randomly choose one of these questions to count for real money. You will receive the reward (either today or in the future) that you chose.’

Regarding the emotion recognition task:

-Page 10 lines 272-286: ‘…reversal occurred after 1 incorrect response or 2 correct responses in a row. Starting difficulty was fixed at 30 on a scale of 5-95 and the step size was fixed at 5, with no separate staircases by emotion. Stopping was not based on reversal counts but once 160 trials had been reached. Emotions were superimposed on 10 unique faces and the number of trials for each emotion was 28 for anger, 26 for happiness, 28 for surprise, 24 for disgust, 25 for sadness and 29 for fear. The emotion shown on each trial was randomized, there was no constraint of time to answer and no fixation between trials (each trial started as soon as the participant answered the previous trial). Instructions to the participants were as follows: ‘This is a study about recognizing emotions. As a token of our appreciation, you will receive a $15 Amazon gift card. This task has multiple trials, and each trial will begin with a choice like this: You'll click on which emotion you think the model is expressing. You will answer these questions about 160 faces with different expressions. Some of the emotions may be easy for you to identify, and some may be hard for you to identify. Please be sure to try your best throughout the experiment! Let’s try a few practice rounds before we begin. Click the button below when you are ready to start.’’

Regarding the memory task:

- Page 10 lines 293-294: ‘Study slide and wait slide were always 30 seconds combined.’

- Pages 10-11 lines 299-320: ‘Regarding study time, for more than 4 correct responses , there was no change in study time. For 5 or 6 correct responses, study time decreased by 4 seconds. For less than 4 correct responses, study time increased by 4 seconds. If study time dropped below 12 seconds, then word count increased by one level and study time was reset to 16 seconds. If study time went above 20 seconds, word count went down one level and study time was reset to 16 seconds. In cases where a participant had all 6 correct responses on a current trial and an overall accuracy of > 70% for all trials or, inversely, less than 3 correct responses on current trial and an overall accuracy of < 60%, the step value was doubled. Participants answered a total of 144 2-alternative forced-choice questions. There was no constraint of time to answer and no fixation between trials (each trial started as soon as the participant answered the previous trial). Instructions to the participants were as follows: ‘This is a study about memory. As a token of our appreciation, you will receive a $15 Amazon gift card. Please scroll through to the bottom of this page to view the structure of this task. Once you reach the bottom of the page, you will find a button to proceed to the next page. This task has multiple trials. For each trial, you will receive an allotted amount of time to memorize the list of words displayed. For example: Next, you'll wait for an amount of time before answering a series of questions about the list of words. Once the waiting time has concluded, we will test your memory for some of the words you have just memorized. We will show you two words at a time, one of which was on the list you just memorized and one that was not. You will answer questions about 24 different lists of words. Some of the lists may be easy to remember, and some may be hard to remember. Please be sure to try your best throughout the experiment! Let’s try a few practice rounds before we begin. Click the button below when you are ready to start.’’

We have also added a paragraph to the Discussion clarifying that drift rate estimates should be interpreted as reflecting the efficiency of evidence accumulation under task conditions individually adjusted to achieve comparable levels of performance, while acknowledging that adaptive procedures may nevertheless limit the direct comparability of drift rate estimates across individuals.

- Page 23 lines 624-631: ‘The adaptive procedures used in the emotion recognition and memory tasks also deserve consideration when interpreting drift rate. These procedures were implemented to maintain participant performance within a comparable accuracy range (approximately 66–70%), thereby reducing floor and ceiling effects and maintaining a similar level of task difficulty across individuals with different abilities. Consequently, although participants were not presented with identical stimulus difficulties, drift rate should be interpreted as reflecting the efficiency of evidence accumulation under task conditions individually adjusted to achieve comparable levels of performance.’

4. Temporal mismatch between behavioural, neuropsychological, and MRI measures

The study allows up to:

• 3 years between behavioural and neuropsychological measures,

• and 4 years between behavioural and MRI acquisition.

Given the advanced age range of the cohort (including participants over 85 years old), this is potentially problematic. White matter integrity, processing speed, and cognitive performance can change substantially over these intervals in older populations.

Although the TBSS analyses included time difference covariates, it is unclear whether the mediation analyses adequately accounted for these temporal discrepancies.

This issue substantially weakens mechanistic interpretations linking white matter changes to DDM parameters.

We thank the reviewer for this important comment. We acknowledge that the interval between online task completion and the linked neuropsychological and neuroimaging assessments is a limitation of this study and reflects the longitudinal design of the cohort, with additional delays introduced by the COVID-19 pandemic. We note that this concern applies only to analyses involving the RT-based speed composite (hypothesis ii) and neuroimaging measures (exploratory hypothesis), as age and online task performance were assessed at the same time.

We now performed a sensitivity analysis restricted to participants who remained cognitively and neurologically healthy at the first clinical evaluation following completion of the online tasks. We also clarify that time difference covariates were also included in the mediation analysis. We added the following sentences to the Methods and Results, respectively:

- Page 14 lines 405-406: ‘The difference (in days) between DTI acquisitions and task performance was used as a covariate.’

- Page 14 lines 417-421: ‘.Given the time gap between online task evaluation and RT-based speed composite and neuroimaging acquisitions, sensitivity analysis for hypothesis ii and exploratory hypothesis iii was performed including only participants for whom a subsequent longitudinal evaluation was available following completion of the online tasks, at which they were confirmed to remain cognitively and neurologically healthy.’

- Page 19 lines 528-533: ‘Table A3 shows results for the sensitivity analysis, including only participants for whom a subsequent longitudinal evaluation was available following completion of the online tasks, at which they were confirmed to remain cognitively and neurologically healthy. Given the reduced sample size, confidence intervals in this sensitivity analysis were wider and comparisons were not statistically significant, although the overall pattern of associations was generally consistent with the primary analyses.’

- Page 17 lines 478-481: ‘For the sensitivity analysis including only participants for whom a subsequent longitudinal evaluation was available following completion of the online tasks, at which they were confirmed to remain cognitively and neurologically healthy, the sample size was reduced to N=77 and bootstrapped unstandardized indirect effect was -3.3e-3 (p = 0.006).’

5. Mediation analyses appear overinterpreted

The mediation findings should be interpreted more cautiously.

The significant mediation effect:

• was modest,

• would not survive correction for multiple comparisons according to the authors themselves,

• was based on cross-sectional data,

• and involved temporally mismatched measures.

Furthermore, the mediation used global FA averages, which substantially reduces anatomical specificity. Meanwhile, the voxelwise TBSS analyses did not show strong converging associations between regional white matter measures and drift rate parameters.

As a result, the manuscript should avoid mechanistic or causal interpretations suggesting that white matter alterations explain age-related slowing in evidence accumulation.

We understand the reviewers’ concern regarding the interpretation of the mediation results. We have now rephrased the sentence in the Abstract:

- Page 1 lines 24-26: ‘…and we show that this association in the emotion recognition task might be partially accounted for by global fractional anisotropy.’

We also rephrased the presentation of this result in the Discussion

- Page 24 lines 661-662: ‘…variables suggests that global FA might partially account for the association between age and drift rate in the emotion recognition task’.

And we added this sentence to the Discussion:

- Page 24 lines 666-669: ‘However, given the marginally significant result we report, that did not survive multiple comparisons correction, this result should be interpreted with caution and be seen as exploratory rather than definite, needing further research to reach more precise conclusions.’

We have now also changed causal chain language throughout the manuscript:

- Page 3 lines 85-88: ‘…and found that the association between age and drift rate showed a significant indirect effect through white matter measures within the frontoparietal network, but the association between age and the non-decision time parameter did not show such effects.’

- Page 5 line 147: ‘…parameter this association is partially accounted for by changes in white matter microstructure.’

- Page 19 line 544: ‘Global FA showed a significant indirect effect in the association between age and drift rate…’

- Page 20 lines 548-550: ‘…nor global MD showed significant indirect effects in the associations between age and drift rate nor in the associations between age and boundary separation and non-decision time…’

- Page 21 lines 580-581: ‘…that white matter microstructure changes have a significant indirect effect in the association between age and drift rate in the emotion recognition task.’

- Page 24 lines 661-662: ‘…suggests that global FA might partially account for the association between age and drift rate…’

6. Statistical analyses are under-modelled relative to the complexity of the questions

The main behavioural analyses rely primarily on simple bivariate Spearman correlations.

Given:

• the broad age range,

• unequal age-group sizes,

• highly educated sample,

• online testing environment,

• adaptive task difficulty,

• and temporally mismatched multimodal data,

this analytic strategy appears overly simplistic.

Particularly concerning is the complete absence of nonlinear analyses. The manuscript repeatedly discusses aging trajectories and lifespan changes, yet all analyses appear effectively monotonic/linear despite the high likelihood of nonlinear aging effects in oldest-old populations.

The manuscript would benefit from:

• regression-based analyses,

• covariate adjustment,

• nonlinear modelling,

• or at minimum stronger justification for the analytic simplicity.

We thank the reviewer for this comment. Our main goal was to examine the associations between age and DDM parameters. Accordingly, our preregistered analyses used Spearman’s rank correlations, a non-parametric approach chosen a priori because it assesses monotonic associations without assuming linear relationships.

We agree that nonlinear age effects exist and have been reported previously (e.g., Krause et al., 2022). However, in that study, drift rate was relatively stable until approximately 60 years of age, after which the estimated decline was mainly monotonic decline. Given that our cohort included participants aged 53–95 years, with the majority of participants over 65 years of age, we considered Spearman’s rank correlation an appropriate approach to quantify monotonic age-related associations within the age range represented in our sample. We also note that our primary analyses were preregistered, and our objective was not to model age-related effects but rather to test the prespecified hypotheses regarding the association between age and DDM parameters. We added this sentence to the methods to clarify this:

-Page 14 lines 394-395: ‘Spearman’s correlation was selected a priori as a non-parametric approach to assess monotonic associations without assuming linear relationships.’

Finally, we have revised the manuscript to avoid language implying that age trajectories were modelled:

- Page 1 line 21: ‘…was to evaluate the cross-sectional age-related differences of processes…’

- Page 4 line 103: ‘…cross-sectional age-related differences of drift rate…’

- Page 27 line 754: ‘…and to be able to look at age-related associations in DDM parameters…’

- Page 27 line 757: ‘…which still limits our ability to characterize these associations in middle age and in later older adulthood.’

7. Multiple comparison concerns remain substantial

The manuscript explicitly states that no multiple-comparison correction was applied.

Although the preregistration and primary/ancillary distinction are appreciated, the study nevertheless includes a large number of:

• correlations,

• DDM parameters,

• tasks,

• mediation analyses,

• and exploratory neuroimaging analyses.

This issue is especially relevant given that some key findings appear statistically fragile.

We thank the reviewer for raising this concern. We have now updated our statistics. For the primary correlations, p-values were adjusted for multiple comparisons across 6 tests of the preregistered hypotheses (associations between age and drift rate for the three tasks, association between RT-based composite measure and drift rate for the three tasks) and by applying Benjamini–Hochberg FDR correction. For ancillary analyses (involving boundary separation and non-decision time), effect sizes are now presented with 95% confidence intervals but without associated p-values to emphasize estimation rather than formal hypothesis testing. P-values adjusted for multiple comparisons across 10 tests (the 6 associations of the primary hypothesis and the 4 mediation models performed) are now reported for the mediation analysis.

Tables 3 and 4 have been updated to reflect these changes and the manuscript has been updated accordingly. The following changes were made to the manuscript:

- Page 5 lines 142-143: ‘Preregistered ancillary analyses were also conducted to examine associations between age and boundary separation and non-decision time.’

- Page 5 lines 145-146: ‘Preregistered ancillary analyses were also conducted to examine associations between RT-based measures and boundary separation and non-decision time.’

- Pages 11-12 lines 331-335: ‘Because our preregistered primary hypothesis was evaluated across three tasks, p-values for the drift rate analyses were adjusted for multiple comparisons using the Benjamini–Hochberg false discovery rate (FDR) procedure. Associations involving boundary separation and non-decision time were considered ancillary analyses and are reported as effect sizes with 95% confidence intervals.’

-Page 14 lines 395-401: ‘Because each primary hypothesis was evaluated across three tasks, p-values for 6 associations were adjusted for multiple comparisons using the Benjamini–Hochberg false discovery rate (FDR) procedure. Additionally, we examined the association between age and the other two diffusion model parameters (boundary-separation and non-decision time) and between the RT-based speed composite and these two parameters, as prespecified ancillary analyses. These ancillary analyses are reported as effect sizes with 95% confidence intervals.’

- Page 14 lines 409-412: ‘P-values for these exploratory analysis were adjusted for 10 comparisons (the 6 associations of the primary hypothesis and the 4 mediation models performed) using the Benjamini–Hochberg false discovery rate (FDR) procedure.’

- Page 17 lines 497-505: ‘Age was significantly negatively associated with drift rate for both the emotion recognition (ρ = -0.19, p = 0.016) and the memory tasks (ρ = -0.18, p = 0.026) (Figure 1 and Table 3). Additionally, age was positively associated with boundary separation in the emotion recognition task (ρ = 0.16, 95% CI [0.017-0.29]), whereas little evidence of an association was observed in the other tasks. Age was also positively associated with non-decision time across all three tasks (delay discounting: ρ = 0.33, 95% CI [0.19-0.46]; emotion recognition: ρ = 0.37, 95% CI [0.24-0.49]; memory: ρ = 0.39, 95% CI [0.25-0.51]) (Table 3 and Appendix Figure A7).’

- Pages 18-19 lines 519-526: ‘The RT-based speed composite was significantly negatively associated with drift rate for both the delay discounting (ρ = -0.274, p = 0.003) and the memory tasks (ρ = -0.271, p = 0.016). In addition, a positive association was found between the RT-based speed composite and boundary separation for the delay discounting (ρ =0.272, 95% CI [0.12-0.41]) and the memory tasks (ρ = 0.223, 95% CI [0.022-0.41]), and the association between the RT-based speed composite and non-decision time was positive across all three tasks (delay discounting: ρ = 0.19, 95% CI [0.03-0.33]; emotion recognition: ρ = 0.33, 95% CI [0.15-0.49]; memory: ρ = 0.25, 95% CI [0.047-0.43]) (Table 4).’

- Pages 19-20 lines 546-548: ‘The bootstrapped unstandardized indirect effect was -1.69e-3 (uncorrected p = 0.036). However, this result did not persist when adjusted for multiple comparisons (p = 0.067).’

Please note that the original uncorrected indirect effect and p-value for the mediation analysis have been corrected from -1.23e-3 to -1.69e-3 and 0.044 to 0.036, respectively. This change reflects a transcription error identified during the revision process.

8. Uneven age distribution limits interpretation of “continuous aging trajectories”

Although the continuous-age approach is preferable to simple young-versus-old comparisons, the sampled age distribution is markedly uneven.

Most participants fall within the 75–84 age range, while middle-aged and oldest-old groups are substantially smaller. This limits the extent to which the analyses can characterize continuous age-related trajectories across the entire sampled range.

Additional descriptive presentation of DDM parameters by age group would help readers evaluate:

• the stability of effects,

• possible nonlinearities,

• and whether findings are disproportionately driven by specific age ranges.

We thank the reviewer for raising this concern. We have now added a Figure to the Appendix (Appendix Figure A6) and added the following sentence:

- Page 16 line 483-484: ‘Appendix Figure A6 shows mean DDM parameter values per age group (age groups can be found in Table 1 - demographic table).’

Minor Issues

1. Definition of “neurologically healthy” is insufficiently specified

The manuscript states that participants were verified as neurologically normal through multidisciplinary assessment. However, no operational criteria are provided.

The manuscript should clarify:

• neuropsychological cutoffs,

• exclusion criteria,

• whether MCI/subclinical impairment was excluded,

• and how “healthy aging” was defined.

This is particularly important given the advanced age of the cohort.

We thank the reviewer for this comment. The following was added to the manuscript:

- Page 6 lines 157-165: ‘During a consensus conference, physicians and clinicians used the patient history (derived in neurologic exam), informant rated Clinical Dementia Rating (CDR), neuropsychological test results, brain Magnetic Resonance Imaging (MRI), and all available fluid and positron emission tomography (PET) biomarkers to establish the diagnosis. Exclusion criteria for the BRANCH cohort include history or current evidence of: large vessel stroke, diagnosis of DSM-5 major psychiatric disorders, multiple sclerosis, symptomatic neurodegenerative disease, epilepsy, significant memory concerns or related diagnoses, active substance abuse, hepatitis C, HIV, syphilis, blindness, or deafness.’

2. Rationale for young-adult normalization of the RT-based composite is unclear

The RT-based speed composite was normalized using a healthy young-adult reference sample.

Because the present study does not include young adults and focuses only on older participants, the rationale for this normalization is unclear. It would help to clarify whether the normalization was intended merely for scaling purposes or whether the resulting measure should be interpreted as age-relative impairment.

We thank the reviewer for this comment. The RT-based speed composite was derived following the methodology of Kerchner et al. (2012), in which individual task response times are standardized relative to a healthy young adult reference sample and then combined into a composite score. The purpose of this normalization is to provide a standardized measure of processing speed on a common scale and to maintain consistency with the previously validated composite measure. It was not intended to represent age-adjusted impairment within our older adult sample. We have clarified this point in the Methods:

- Page 7 lines 199-202: ‘The normalization to a young adult reference group was retained to maintain consistency with the validated composite measure described by Kerchner et al. (2012) [2], providing a standardized metric of processing speed rather than an age-adjusted measure of impairment.’

3. Trial trimming procedure deserves further discussion

The exclusion of the slowest 5% of responses was justified by concerns regarding distraction during online testing. While understandable, extremely slow responses may also reflect genuine aging-related variability rather than artifacts.

The potential influence of trimming on DDM parameter estimation should be discussed more explicitly.

We thank the reviewer for this concern. We agree that very slow responses may reflect genuine age-related variability as well as lapses in attention or environmental distractions. The exclusion of the slowest 5% of responses was chosen a priori because the tasks were completed in an unconstrained home environment, where unusually slow responses are more likely to reflect interruptions or off-task processes than in laboratory-based testing.

However, to evaluate the potential influence of this step, we repeated the analyses including all responses (sensitivity analysis in Appendix Tables A1 and A2). The overall pattern of results remained largely unchanged, suggesting that our conclusions are not driven by the exclusion of the slowest 5% of responses. We have also acknowledged now in the Discussion that some very slow responses may represent genuine age-related variability rather than only environmental distraction:

- Page 19 line 527: ‘Table A2 shows results for the sensitivity analysis, without excluding the 5% slowest trials.’

- Page 25 lines 707-710: ‘A related issue was the exclusion of the slowest 5% of responses, that was intended to reduce the influence of lapses and environmental distractions inherent to unsupervised home testing. However, some of these responses may also reflect genuine age-related variability. Nevertheless, our sensitivity analyses, including all responses, yielded largely comparable results.’

Strengths

Despite the concerns above, the manuscript has several important strengths:

• preregistration,

• inclusion of oldest-old adults,

• use of continuous age rather than dichotomous grouping,

• explicit model-fit assessment,

• sensitivity analyses,

• computational modelling approach,

• and integration of behavioural and neuroimaging measures.

The model-fit evaluation procedure, including Monte Carlo simulations and graphical diagnostics, is particularly careful and represents a methodological strength of the study.

Overall Evaluation

This manuscript addresses an important topic and includes several valuable methodological features. However, the current interpretation of the findings is, in my view, more conceptually unified and mechanistically specific than the data presently justify.

The manuscript would become substantially stronger if the authors:

• adopted a more cautious interpretation of drift rate,

• more explicitly acknowledged task heterogeneity,

• clarified the theoretical role of the three paradigms,

• reduced mechanistic claims,

• and more carefully addressed the limitations introduced by adaptive task procedures and temporally mismatched multimodal measures.

At present, the data may be better suited to supporting a nuanced account of task-dependent aging effects on DDM-derived parameters than a more general account of age-related slowing of information accumulation.

We thank the reviewer for this constructive overall assessment of our work, as well as for recognizing the strengths of the study. In response to the reviewer’s comments, we have revised the manuscript to adopt a more cautious interpretation of the findings, reduce mechanistic language throughout, provide additional methodological details for the experimental paradigms, include new sensitivity and parameter recovery analyses, and expand the discussion of the adaptive task procedures and the temporal separation between behavioral, neuropsychological, and neuroimaging measures. We believe these revisions have strengthened the manuscript and improved the clarity and interpretation of our findings.

Attachment Submitted filename: Response to reviewers.docx

# Peer review: Decision Letter 1

**DOI:** 10.1371/journal.pone.0361899.r003

**Authors:** Pramod Yadav

10 Aug 2026

Dear Dr. Sanches,

Thank you for submitting your manuscript to PLOS ONE. After careful consideration, we feel that it has merit but does not fully meet PLOS ONE’s publication criteria as it currently stands. Therefore, we invite you to submit a revised version of the manuscript that addresses the points raised during the review process.

Please submit your revised manuscript by Sep 24 2026 11:59PM. If you will need more time than this to complete your revisions, please reply to this message or contact the journal office at plosone@plos.org. When you're ready to submit your revision, log on to https://www.editorialmanager.com/pone/ and select the 'Submissions Needing Revision' folder to locate your manuscript file.

- A letter that responds to each point raised by the academic editor and reviewer(s). You should upload this letter as a separate file labeled 'Response to Reviewers'.
- A marked-up copy of your manuscript that highlights changes made to the original version. You should upload this as a separate file labeled 'Revised Manuscript with Track Changes'.
- An unmarked version of your revised paper without tracked changes. You should upload this as a separate file labeled 'Manuscript'.

If you would like to make changes to your financial disclosure, please include your updated statement in your cover letter. Guidelines for resubmitting your figure files are available below the reviewer comments at the end of this letter.

If applicable, we recommend that you deposit your laboratory protocols in protocols.io to enhance the reproducibility of your results. Protocols.io assigns your protocol its own identifier (DOI) so that it can be cited independently in the future. For instructions see: https://journals.plos.org/plosone/s/submission-guidelines#loc-laboratory-protocols. Additionally, PLOS ONE offers an option for publishing peer-reviewed Lab Protocol articles, which describe protocols hosted on protocols.io. Read more information on sharing protocols at https://plos.org/protocols?utm_medium=editorial-email&utm_source=authorletters&utm_campaign=protocols.

As the corresponding author, your ORCID iD is verified in the submission system and will appear in the published article. PLOS supports the use of ORCID, and we encourage all coauthors to register for an ORCID iD and use it as well. Please encourage your coauthors to verify their ORCID iD within the submission system before final acceptance, as unverified ORCID iDs will not appear in the published article. *Only*  the individual author can complete the verification step; PLOS staff *cannot*  verify ORCID iDs on behalf of authors.

We look forward to receiving your revised manuscript.

Kind regards,

Pramod K. Yadav, Ph. D.

Academic Editor

PLOS One

Journal Requirements:

If the reviewer comments include a recommendation to cite specific previously published works, please review and evaluate these publications to determine whether they are relevant and should be cited. There is no requirement to cite these works unless the editor has indicated otherwise.

[Note: HTML markup is below. Please do not edit.]

Reviewers' comments:

Reviewer’s Responses to Questions

**Comments to the Author**

Reviewer #1: All comments have been addressed

Reviewer #2: (No Response)

**********

2. Is the manuscript technically sound, and do the data support the conclusions?

Reviewer #1: Yes

Reviewer #2: (No Response)

**********

3. Has the statistical analysis been performed appropriately and rigorously?

Reviewer #1: Yes

Reviewer #2: (No Response)

**********

4. Have the authors made all data underlying the findings in their manuscript fully available?

The PLOS Data policy

Reviewer #1: Yes

Reviewer #2: (No Response)

**********

5. Is the manuscript presented in an intelligible fashion and written in standard English?

Reviewer #1: Yes

Reviewer #2: (No Response)

**********

Reviewer #1: General Comments

The authors investigate age-related differences in cognitive processing speed utilizing the drift diffusion model across an older adult cohort. The major weakness of the manuscript centers on the analytical framework applied to the delay discounting task. The methodology utilizes a maximum likelihood estimation to determine a hyperbolic discounting parameter prior to fitting the drift diffusion model. This conditional two-stage procedure creates a structural discrepancy when compared to the accuracy-based coding utilized in the emotion recognition and memory tasks. Consequently, comparing the drift rate parameter across these disparate tasks lacks robust validity. A secondary major weakness relates to the neuroimaging mediation analysis. The sample size decreases substantially for the diffusion tensor imaging models, and the reported mediation effect of fractional anisotropy on the association between age and drift rate in the emotion recognition task did not survive false discovery rate correction. The minor weaknesses involve the data collection environment. The experimental tasks were administered online in unconstrained home settings, which introduces uncontrolled variables such as environmental distractors that directly influence the primary dependent variable of reaction time.

Scientific Content, Statistics, and Methodology

The overall scientific content is sound, yet the adaptive difficulty procedures applied to the emotion recognition and memory tasks require careful interpretation. The staircase algorithms were programmed to maintain participant accuracy between 66 and 70 percent. Modifying stimulus difficulty individually means the drift rate parameter measures evidence accumulation under heterogenous testing conditions rather than standardized objective difficulty. The statistical approach appropriately utilizes Spearman correlations for non-parametric assessment of monotonic associations. The authors properly applied the Benjamini-Hochberg false discovery rate procedure for multiple comparisons regarding their primary hypotheses. The methodology of excluding the slowest 5 percent of reaction times and trials faster than 350 milliseconds is a standard and acceptable practice to mitigate the impact of the unsupervised online testing environment. The tract-based spatial statistics correctly utilized permutation testing and threshold-free cluster enhancement, establishing rigorous criteria for the imaging data.

Specific Comments

Page 7, lines 174-181: The methodology permits a time gap of up to four years between online experimental task performance and neuroimaging acquisition. Significant microstructural white matter changes and cortical atrophy occur over a four-year period in cohorts ranging up to 95 years of age, which severely confounds the assumption of cross-sectional alignment between the behavioral and imaging datasets.

Page 10, lines 240-262: The delay discounting task presents trials immediately after a participant responds without a fixed inter-trial interval or fixation cross. Proceeding without a fixation period fails to control for residual attention or motor preparation from the preceding trial, introducing uncontrolled variance into the non-decision time parameter.

Page 12, lines 294-319: The memory task dynamically adjusts study time, delay time, and the total number of words based on ongoing performance accuracy. The manuscript lacks an analysis of how these specific adaptive variables independently influence the estimated drift rate, complicating the isolation of pure age-related cognitive slowing from task-induced strategic shifts.

Page 13, lines 342-352: Calculating a subjective value function before applying the drift diffusion model creates an inconsistent analytical foundation relative to the other assessments. This difference in response coding limits the ability to draw unified conclusions regarding information accumulation across the tested domains.

Page 24, lines 661-669: The mediation models evaluating global fractional anisotropy present a marginally significant unstandardized indirect effect that became non-significant after adjusting for multiple comparisons. The discussion surrounding white matter connectivity mediating the effects of age on drift rate must be explicitly described as statistically inconclusive.

Reviewer #2: I appreciate the considerable effort the authors have invested in revising the manuscript. The revision includes several useful additions, including more detailed task descriptions, multiple-comparison correction, reaction-time sensitivity analyses, parameter-recovery analyses, and a more explicit discussion of several limitations. These changes have improved the transparency and reproducibility of the study.

Nevertheless, after comparing the response letter with the revised manuscript and the newly reported analyses, I do not think that all of the central concerns raised in the previous round have been adequately resolved. In several instances, the response provides a rationale for retaining the original approach but does not fully address the underlying construct-validity or interpretational concern. There are also places where the response letter claims that a limitation has been acknowledged or that causal language has been removed, whereas the quoted revised text does not consistently reflect that change.

My principal remaining concerns are outlined below.

1. The interpretation of drift rate across the three tasks remains overly general

The authors provide a reasonable justification for applying the drift diffusion model to heterogeneous cognitive tasks. I agree that DDMs have been productively applied to perceptual, mnemonic, and value-based decisions, and that it can be informative to examine DDM parameters across different tasks within the same cohort.

However, this does not resolve the construct-validity issue raised in the previous review. The fact that the same mathematical model can be applied to different tasks does not establish that drift rate has the same psychological meaning across those tasks. In the present study, drift rate may reflect:

• perceptual-social evidence in the emotion-recognition task;

• mnemonic evidence, familiarity, or retrieval processes in the memory task; and

• subjective-value differences, decision conflict, or preference consistency in the delay-discounting task.

These can all be described as forms of evidence accumulation, but they should not automatically be treated as interchangeable measures of a unitary cognitive processing-speed construct.

The authors now state that their argument does not depend on the three operations being psychologically equivalent. This is an important concession, but it is not fully reconciled with the broader framing and conclusions of the manuscript, which continue to interpret drift rate as a relatively general measure of information-processing or cognitive processing speed. If the tasks are not assumed to be psychologically equivalent, the corresponding drift rates should instead be described consistently as task-specific indices of evidence accumulation.

A stronger claim about a common, task-general processing-speed construct would require relevant convergent evidence, such as substantial correlations among the task-specific drift rates, formal latent-variable modelling, or another analysis demonstrating shared variance. In the absence of such evidence, the study can still make a useful contribution, but its contribution should be framed as examining age-related associations with task-specific DDM parameters across heterogeneous decision contexts.

This issue is particularly important for delay discounting. The participant-specific discounting parameter (k) is estimated from the choices, and the same choices are subsequently classified as consistent or inconsistent with the preference function defined by that estimate. Drift rate in this model is therefore likely to reflect the consistency or certainty of choices relative to the fitted preference function, rather than cognitive processing speed in the same sense as in a perceptual or memory decision. The analogy between preference-consistent and objectively correct responses does not eliminate this conceptual difference.

The manuscript should therefore:

• avoid presenting the three drift-rate estimates as directly comparable measures of a single processing-speed construct;

• describe their psychological interpretation separately for each task;

• state explicitly that the evidence for a common age-related mechanism across tasks was exploratory and inconclusive; and

• revise the Abstract, Introduction, Discussion, and conclusions accordingly.

2. The implications of the adaptive task procedures remain insufficiently addressed

The revised Methods provide substantially more information about the adaptive procedures, which is helpful. However, the central interpretational problem remains.

The response letter states that the revised Discussion acknowledges that adaptive procedures “may nevertheless limit the direct comparability of drift rate estimates across individuals.” Yet the quoted text added to the manuscript does not contain this acknowledgement. Instead, it concludes that drift rate should be interpreted as evidence-accumulation efficiency “under task conditions individually adjusted to achieve comparable levels of performance.”

Comparable accuracy does not necessarily imply comparable stimulus difficulty or comparable latent decision processes. Participants may achieve similar accuracy while being exposed to different emotion intensities, list lengths, encoding durations, or retention conditions. Because drift rate is sensitive to stimulus discriminability and task difficulty, individual differences in the adaptive levels attained may directly influence the estimated drift rates.

This is especially problematic in the memory task, in which more than one task dimension appears to be adapted. It is therefore difficult to determine whether individual differences in drift rate reflect evidence-accumulation efficiency, memory ability, the task difficulty reached by the staircase, or some combination of these factors.

If the necessary task-level variables are available, the authors should report:

• the distribution of the mean or final difficulty levels;

• their associations with age;

• their associations with drift rate; and

• whether the age–drift-rate associations remain after accounting for achieved task difficulty.

If these analyses cannot be performed, the limitation should be stated directly and more strongly. The manuscript should not imply that equating average performance necessarily makes drift-rate estimates directly comparable across individuals.

3. The temporal mismatch has not been adequately addressed by the reported sensitivity analysis

It is useful that the difference in days between DTI acquisition and online task performance was included as a covariate in the mediation analysis. However, the additional sensitivity analysis does not directly test the effect of temporal mismatch.

Restricting the sample to participants who remained cognitively and neurologically healthy at a subsequent clinical evaluation addresses the possibility of overt intervening neurological or cognitive disorder. It does not establish that white matter integrity or processing speed remained stable during the three- to four-year interval between measurements. Normal age-related changes can occur even in individuals who remain clinically healthy.

Moreover, the manuscript’s statement that the pattern of associations was “generally consistent with the primary analyses” is not well supported by Appendix Table A3. For the association between the RT-based speed composite and drift rate, the estimates changed as follows:

• delay discounting: approximately (\rho=-.27) to (\rho=-.17);

• emotion recognition: approximately (\rho=-.09) to (\rho=+.08);

• memory: approximately (\rho=-.27) to (\rho=-.05).

Thus, two associations were substantially attenuated and one changed direction. This cannot be described solely as a loss of statistical significance caused by wider confidence intervals. The Results and Discussion should report this attenuation accurately.

Ideally, the authors should conduct sensitivity analyses using progressively narrower measurement intervals, even if the resulting estimates are imprecise, or model the temporal difference continuously in all analyses involving non-concurrent measures. At minimum, the manuscript must clearly distinguish between sensitivity to subsequent clinical status and sensitivity to measurement timing, as these are not equivalent.

The mediation sensitivity result reported for the reduced sample, (p=.006), should also be identified explicitly as either corrected or uncorrected. Its inferential status is currently unclear.

4. The mediation result remains overinterpreted and is described inconsistently

The authors report an uncorrected indirect-effect (p)-value of .036, which becomes .067 after multiple-comparison correction. Accordingly, this result does not meet the inferential criterion adopted in the revised manuscript.

Despite this, the manuscript continues to use statements such as:

• “Global FA showed a significant indirect effect”;

• “white matter microstructure changes have a significant indirect effect”;

• “this association is partially accounted for by changes in white matter microstructure”; and

• “global FA might partially account for the association.”

These formulations are not fully consistent with the corrected result. Some also continue to imply a mechanistic or causal chain, despite the cross-sectional design and temporal separation of the measures. The response letter states that causal language has been changed throughout the manuscript, but several of the examples presented as revised wording still retain mechanistic implications.

The Abstract formulation—“we show that this association … might be partially accounted for by global fractional anisotropy”—is also too strong. The phrase “we show” is difficult to reconcile with an exploratory effect that did not survive correction.

A more accurate formulation would be:

“An exploratory indirect association involving global FA was observed in the emotion-recognition task, but it did not remain statistically significant after correction for multiple comparisons.”

Similar wording should be used consistently in the Results and Discussion. The authors should avoid describing the effect as significant without immediately specifying that this refers only to the uncorrected result. They should also avoid “account for,” “mediate the effects of age,” or equivalent mechanistic language.

The result may be reported as hypothesis-generating, but it should not support substantive conclusions about white matter explaining age-related changes in drift rate.

5. The conclusions concerning differences between tasks should be further softened

The authors appropriately acknowledge that a significant association in two tasks and a nonsignificant association in a third task do not demonstrate a statistically significant difference between tasks. This is a welcome improvement.

Nevertheless, expressions such as “may be more evident in some cognitive domains than others” and “may be more apparent” still invite a between-task interpretation that was not formally tested. Given that the delay-discounting association was in the same direction as the other associations, and given the differences in task architecture, response coding, trial numbers, and adaptive procedures, the safest conclusion is that evidence for an association was obtained in two tasks but not in the third. The data do not establish that the population-level associations differ across tasks.

The manuscript should distinguish clearly between:

• evidence for an association within each task; and

• evidence that the magnitude of the association differs between tasks.

Only the former was tested.

6. The delay-discounting model requires further clarification

The authors appropriately acknowledge that the delay-discounting DDM is conditional on the first-stage estimate of (k), and they now discuss joint hierarchical modelling as a preferable future approach. This improves the manuscript.

However, two additional issues should be clarified.

First, the Methods indicate that (k) was estimated with bounds of (-10^{10}) and (10^{10}). A standard hyperbolic temporal-discounting parameter is ordinarily constrained to a theoretically meaningful non-negative range. Allowing very large negative values may create theoretically invalid discount functions and potential singularities in (V=A/(1+kD)). The authors should justify these bounds, report whether any negative estimates occurred, and preferably repeat the estimation using theoretically appropriate constraints.

Second, because (k) is estimated from the same choices that are subsequently coded as consistent or inconsistent, the resulting DDM parameter should be interpreted cautiously. It is not equivalent to a drift rate estimated from independently defined correct and incorrect responses. This difference needs to be reflected not only in the limitations but also in the substantive interpretation of the delay-discounting results.

7. The parameter-recovery analysis is useful but should not be overstated

The addition of a parameter-recovery analysis is welcome. Nevertheless, high correlations between empirical parameter estimates and estimates recovered from data simulated from those same fitted parameters do not provide a complete assessment of parameter recovery.

Correlations can be high when there is substantial between-participant variation even if estimates are systematically biased or imprecise. A more informative evaluation would also report measures such as bias, root mean squared error, regression slope and intercept, or interval coverage, ideally across repeated simulations.

If the authors retain the current analysis, it should be described more narrowly as evidence that the rank ordering of participants was reasonably recoverable under the fitted model and observed trial counts, rather than as comprehensive evidence of “good recovery.” The inability to assess test–retest reliability is understandable and should remain acknowledged.

8. Multiple-comparison correction is improved but requires clearer reporting

The introduction of FDR correction represents an important improvement. However, the correction procedure and the status of the reported p-values should be described more consistently.

The Methods should state clearly which families of tests were subjected to FDR correction and whether the p-values subsequently reported in the text, tables, and figures are adjusted unless explicitly identified otherwise. Standalone tables and figure captions should also provide sufficient information to be interpreted independently. For example, the Figure 1 caption should refer to an FDR-adjusted significance threshold rather than simply stating that associations were significant “at a p-value of 0.05.” Unadjusted p-values may be reported for transparency, but all inferential conclusions should be based on the adjusted values.

The following points also require clarification:

• whether the p-values reported for the sensitivity analyses are adjusted or unadjusted;

• whether the mediation sensitivity result of (p=.006) is adjusted or unadjusted;

• the exact tests included in each FDR family; and

• whether the ancillary mediation analyses involving boundary separation and non-decision time were included in any multiplicity correction.

In addition, the rationale for combining six preregistered behavioural associations and four exploratory mediation tests into a single ten-test FDR family is unclear. These tests address conceptually distinct hypotheses, and the mediation analyses appear to have been conducted conditionally, following evidence of associations in the preceding analyses. Combining them into one family may therefore allow the rank ordering of strong behavioural p-values to influence the adjustment of conceptually separate exploratory mediation results. The authors should either provide a clear statistical and conceptual justification for this combined family or apply separately defined correction families to the behavioural and mediation analyses. If the correction procedure is revised, all affected adjusted p-values and corresponding conclusions should be updated accordingly.

9. Issues that have been addressed adequately

Several previous concerns have been addressed sufficiently and, in my view, need not prevent publication after the remaining issues are resolved.

The authors have:

• substantially expanded the task descriptions;

• clarified the definition and clinical evaluation of neurologically healthy participants;

• explained the purpose of normalization to the young-adult reference sample;

• replaced longitudinal terminology with cross-sectional terminology;

• acknowledged the uneven age distribution and limited oldest-old sample;

• added descriptive presentation by age group;

• reported diffusion-specific quality-control procedures and motion statistics;

• provided reaction-time distributions before and after trimming;

• repeated the principal analyses without trimming the slowest 5% of trials; and

• justified the preregistered use of Spearman correlations as tests of monotonic associations.

I consider the explanation for not fitting nonlinear age trajectories broadly acceptable. A nonlinear exploratory analysis could still be informative, but it is not essential to the preregistered monotonic association hypothesis, provided that the manuscript no longer makes claims about developmental or aging trajectories.

Overall assessment

The manuscript has improved, and I recognize that the authors have made a serious effort to respond to a large number of comments. The study has meaningful strengths, including preregistration, a relatively large older-adult sample, explicit model-fit diagnostics, sensitivity analyses, and the integration of behavioural modelling with neuroimaging measures.

However, several central concerns have not yet received satisfactory answers. In particular, the manuscript still moves too readily from task-specific DDM parameters to a general cognitive processing-speed interpretation; the consequences of adaptive task difficulty are not adequately analysed or acknowledged; the temporal sensitivity analysis does not test temporal proximity and is described more positively than its results warrant; and the exploratory, non-significant-after-correction mediation effect continues to be presented using significant and partly mechanistic language.

Therefore, I would support a further substantial revision focused on aligning the claims with what the analyses can establish. The study may ultimately support a useful and appropriately nuanced account of age-related associations with task-specific DDM parameters. It does not currently provide convincing evidence for a unitary, task-general slowing of evidence accumulation or for white matter microstructure as a mechanism explaining that slowing.

**********

what does this mean?). If published, this will include your full peer review and any attached files.

If you choose “no”, your identity will remain anonymous but your review may still be made public.

**Do you want your identity to be public for this peer review?**  For information about this choice, including consent withdrawal, please see our Privacy Policy

Reviewer #1: **Yes:**  Wissem DHahbi

Reviewer #2: No

**********

[NOTE: If reviewer comments were submitted as an attachment file, they will be attached to this email and accessible via the submission site. Please log into your account, locate the manuscript record, and check for the action link "View Attachments". If this link does not appear, there are no attachment files.]

To ensure your figures meet our technical requirements, please review our figure guidelines: https://journals.plos.org/plosone/s/figures

You may also use PLOS’s free figure tool, NAAS, to help you prepare publication quality figures: https://journals.plos.org/plosone/s/figures#loc-tools-for-figure-preparation.

NAAS will assess whether your figures meet our technical requirements by comparing each figure against our figure specifications.

# Author comment: Author response to Decision Letter 2

**DOI:** 10.1371/journal.pone.0361899.r004

23 Sep 2026

Reviewer #1: General Comments

The authors investigate age-related differences in cognitive processing speed utilizing the drift diffusion model across an older adult cohort. The major weakness of the manuscript centers on the analytical framework applied to the delay discounting task. The methodology utilizes a maximum likelihood estimation to determine a hyperbolic discounting parameter prior to fitting the drift diffusion model. This conditional two-stage procedure creates a structural discrepancy when compared to the accuracy-based coding utilized in the emotion recognition and memory tasks. Consequently, comparing the drift rate parameter across these disparate tasks lacks robust validity. A secondary major weakness relates to the neuroimaging mediation analysis. The sample size decreases substantially for the diffusion tensor imaging models, and the reported mediation effect of fractional anisotropy on the association between age and drift rate in the emotion recognition task did not survive false discovery rate correction. The minor weaknesses involve the data collection environment. The experimental tasks were administered online in unconstrained home settings, which introduces uncontrolled variables such as environmental distractors that directly influence the primary dependent variable of reaction time.

Scientific Content, Statistics, and Methodology

The overall scientific content is sound, yet the adaptive difficulty procedures applied to the emotion recognition and memory tasks require careful interpretation. The staircase algorithms were programmed to maintain participant accuracy between 66 and 70 percent. Modifying stimulus difficulty individually means the drift rate parameter measures evidence accumulation under heterogenous testing conditions rather than standardized objective difficulty. The statistical approach appropriately utilizes Spearman correlations for non-parametric assessment of monotonic associations. The authors properly applied the Benjamini-Hochberg false discovery rate procedure for multiple comparisons regarding their primary hypotheses. The methodology of excluding the slowest 5 percent of reaction times and trials faster than 350 milliseconds is a standard and acceptable practice to mitigate the impact of the unsupervised online testing environment. The tract-based spatial statistics correctly utilized permutation testing and threshold-free cluster enhancement, establishing rigorous criteria for the imaging data.

Specific Comments

- Page 7, lines 174-181: The methodology permits a time gap of up to four years between online experimental task performance and neuroimaging acquisition. Significant microstructural white matter changes and cortical atrophy occur over a four-year period in cohorts ranging up to 95 years of age, which severely confounds the assumption of cross-sectional alignment between the behavioral and imaging datasets.

- Page 10, lines 240-262: The delay discounting task presents trials immediately after a participant responds without a fixed inter-trial interval or fixation cross. Proceeding without a fixation period fails to control for residual attention or motor preparation from the preceding trial, introducing uncontrolled variance into the non-decision time parameter.

- Page 12, lines 294-319: The memory task dynamically adjusts study time, delay time, and the total number of words based on ongoing performance accuracy. The manuscript lacks an analysis of how these specific adaptive variables independently influence the estimated drift rate, complicating the isolation of pure age-related cognitive slowing from task-induced strategic shifts.

- Page 13, lines 342-352: Calculating a subjective value function before applying the drift diffusion model creates an inconsistent analytical foundation relative to the other assessments. This difference in response coding limits the ability to draw unified conclusions regarding information accumulation across the tested domains.

- Page 24, lines 661-669: The mediation models evaluating global fractional anisotropy present a marginally significant unstandardized indirect effect that became non-significant after adjusting for multiple comparisons. The discussion surrounding white matter connectivity mediating the effects of age on drift rate must be explicitly described as statistically inconclusive.

We are grateful again to the reviewer for their careful and conscientious attention to the manuscript. As this reviewer indicated that previous comments have been addressed, we have not written a point-by-point response to these additional notes. However, our comments to Reviewer 2 indicate several changes made to the manuscript that also address Reviewer 1’s residual concerns. The key remaining issue described by Reviewer 1 concerns the relationship of findings from the delay discounting task to findings from the other two tasks, which is addressed in greater detail below. Their secondary concern is about the interpretation of the exploratory neuroimaging mediation analysis, also addressed in greater detail below. Regarding the home testing environment, we have described our work to address this concern to the extent possible.

Other specific comments are also addressed below, including sensitivity analyses addressing the time gap between experimental task performance and in-person neuroimaging measures and the influence of adaptive variables on drift rate. This reviewer also notes that the lack of a fixation cross or fixed inter-trial interval may introduce variance into the non-decision time parameter. In the revised manuscript, we have further clarified the manuscript’s preregistered focus on the drift rate parameter, and have restated results related to the non-decision time parameter to clarify that hypotheses regarding this parameter were not specifically assessed.

Reviewer #2: I appreciate the considerable effort the authors have invested in revising the manuscript. The revision includes several useful additions, including more detailed task descriptions, multiple-comparison correction, reaction-time sensitivity analyses, parameter-recovery analyses, and a more explicit discussion of several limitations. These changes have improved the transparency and reproducibility of the study.

Nevertheless, after comparing the response letter with the revised manuscript and the newly reported analyses, I do not think that all of the central concerns raised in the previous round have been adequately resolved. In several instances, the response provides a rationale for retaining the original approach but does not fully address the underlying construct-validity or interpretational concern. There are also places where the response letter claims that a limitation has been acknowledged or that causal language has been removed, whereas the quoted revised text does not consistently reflect that change.

My principal remaining concerns are outlined below.

1. The interpretation of drift rate across the three tasks remains overly general

The authors provide a reasonable justification for applying the drift diffusion model to heterogeneous cognitive tasks. I agree that DDMs have been productively applied to perceptual, mnemonic, and value-based decisions, and that it can be informative to examine DDM parameters across different tasks within the same cohort.

However, this does not resolve the construct-validity issue raised in the previous review. The fact that the same mathematical model can be applied to different tasks does not establish that drift rate has the same psychological meaning across those tasks. In the present study, drift rate may reflect:

• perceptual-social evidence in the emotion-recognition task;

• mnemonic evidence, familiarity, or retrieval processes in the memory task; and

• subjective-value differences, decision conflict, or preference consistency in the delay-discounting task.

These can all be described as forms of evidence accumulation, but they should not automatically be treated as interchangeable measures of a unitary cognitive processing-speed construct.

The authors now state that their argument does not depend on the three operations being psychologically equivalent. This is an important concession, but it is not fully reconciled with the broader framing and conclusions of the manuscript, which continue to interpret drift rate as a relatively general measure of information-processing or cognitive processing speed. If the tasks are not assumed to be psychologically equivalent, the corresponding drift rates should instead be described consistently as task-specific indices of evidence accumulation.

A stronger claim about a common, task-general processing-speed construct would require relevant convergent evidence, such as substantial correlations among the task-specific drift rates, formal latent-variable modelling, or another analysis demonstrating shared variance. In the absence of such evidence, the study can still make a useful contribution, but its contribution should be framed as examining age-related associations with task-specific DDM parameters across heterogeneous decision contexts.

This issue is particularly important for delay discounting. The participant-specific discounting parameter (k) is estimated from the choices, and the same choices are subsequently classified as consistent or inconsistent with the preference function defined by that estimate. Drift rate in this model is therefore likely to reflect the consistency or certainty of choices relative to the fitted preference function, rather than cognitive processing speed in the same sense as in a perceptual or memory decision. The analogy between preference-consistent and objectively correct responses does not eliminate this conceptual difference.

The manuscript should therefore:

• avoid presenting the three drift-rate estimates as directly comparable measures of a single processing-speed construct;

• describe their psychological interpretation separately for each task;

• state explicitly that the evidence for a common age-related mechanism across tasks was exploratory and inconclusive; and

• revise the Abstract, Introduction, Discussion, and conclusions accordingly.

We thank the reviewer for this comment and agree that application of the same mathematical model across heterogeneous tasks does not imply that drift rate has an identical psychological meaning across tasks. To directly examine the extent of shared variance, we have now conducted exploratory pairwise Spearman correlations between drift rate estimates from the three paradigms. Drift rates were correlated between the memory and emotion recognition tasks (ρ = 0.32, 95% CI [0.18, 0.46]) and between the emotion recognition and delay discounting tasks (ρ= 0.25, 95% CI [0.05, 0.43]), whereas the association between memory and delay discounting drift rates was smaller and not statistically significant (ρ = 0.12, 95% CI [-0.1, 0.32]; these two tasks had the smallest overlap in participants) (these analyses have been added to the Appendix Figure A7). Thus, these analyses provide some evidence of shared variance, but indeed do not support treating the three drift rate estimates as interchangeable measures of a unitary processing-speed construct.

We have revised the manuscript accordingly. Drift rate is now interpreted in relation to perceptual/social evidence in the emotion recognition task, mnemonic evidence in the memory task, and subjective-value-related evidence in the delay discounting task. We have also revised the broader framing and conclusions to describe the study as examining age-related associations with task-specific information processing across heterogeneous cognitive contexts.

- Page 1 lines 26-27: ‘Our results suggest age-related differences in information processing across cognitive contexts…’

- Page 5 line 129: ‘ … - emotion recognition, memory, and delay discounting - …’

- Page 5 lines 132-137: ‘We primarily evaluated the effects of age on the drift rate parameter, as it reflects the rate of information accumulation during decision-making. Although drift rate provides a common mathematical parameter of information accumulation across the three tasks, the nature of the information being accumulated varies with task: perceptual/social information in the emotion recognition task, mnemonic information in the memory task, and information related to subjective valuation in the delay discounting task.’

- Page 14 lines 407-410: ‘Additionally, we conducted exploratory pairwise Spearman correlations between drift rate estimates from the three tasks to assess the degree of correspondence in drift rate across task contexts. These are reported descriptively as effect sizes with 95% confidence intervals.’

- Page 17 lines 505-506: ‘Appendix Figure A7 shows the pairwise Spearman correlations between drift rate estimates from the three tasks.’

- Pages 23 lines 630-632: ‘… (reflecting slower information accumulation within a given task context) with evidence for this association observed in two of the three cognitive domains examined. ’

- Pages 26 lines 729-730: ‘…information processing across cognitive contexts and that these task-specific processes should be considered…’

- Page 28 lines 775-778: ‘Indeed, the modest and inconsistent pairwise associations observed between drift rates across tasks suggest that these parameters may be interpreted as task-specific indices of information accumulation rather than as interchangeable measures of a unitary processing-speed construct.’

- Page 29 lines 816-817: ‘…reflected in task-specific patterns of evidence accumulation across cognitive domains…’

2. The implications of the adaptive task procedures remain insufficiently addressed

The revised Methods provide substantially more information about the adaptive procedures, which is helpful. However, the central interpretational problem remains.

The response letter states that the revised Discussion acknowledges that adaptive procedures “may nevertheless limit the direct comparability of drift rate estimates across individuals.” Yet the quoted text added to the manuscript does not contain this acknowledgement. Instead, it concludes that drift rate should be interpreted as evidence-accumulation efficiency “under task conditions individually adjusted to achieve comparable levels of performance.”

Comparable accuracy does not necessarily imply comparable stimulus difficulty or comparable latent decision processes. Participants may achieve similar accuracy while being exposed to different emotion intensities, list lengths, encoding durations, or retention conditions. Because drift rate is sensitive to stimulus discriminability and task difficulty, individual differences in the adaptive levels attained may directly influence the estimated drift rates.

This is especially problematic in the memory task, in which more than one task dimension appears to be adapted. It is therefore difficult to determine whether individual differences in drift rate reflect evidence-accumulation efficiency, memory ability, the task difficulty reached by the staircase, or some combination of these factors.

If the necessary task-level variables are available, the authors should report:

• the distribution of the mean or final difficulty levels;

• their associations with age;

• their associations with drift rate; and

• whether the age–drift-rate associations remain after accounting for achieved task difficulty.

If these analyses cannot be performed, the limitation should be stated directly and more strongly. The manuscript should not imply that equating average performance necessarily makes drift-rate estimates directly comparable across individuals.

We thank the reviewer for this comment and agree that maintaining comparable accuracy across participants does not necessarily imply equivalent stimulus difficulty, and that variation in the adaptive levels attained may contribute to individual differences in drift rate.

We have now performed the suggested additional analyses for both tasks. Specifically, we examined the distributions of the final difficulty levels, their associations with age and drift rate, and whether the associations between age and drift rate remained after accounting for achieved task difficulty.

For the emotion recognition task, the association between final adaptive level and age was ρ = 0.21 (95% CI [0.08, 0.35]), while the association between final adaptive level and drift rate was ρ = −0.15 (95% CI [−0.29, −0.01]). For the memory task, the corresponding associations were ρ = 0.12 (95% CI [−0.03, 0.27]) and ρ = −0.24 (95% CI [−0.38, −0.10]), respectively. Lower values of the adaptive parameters correspond to greater task difficulty. Importantly, partial Spearman correlations controlling for task difficulty showed that the negative association between age and drift rate remained significant for both the emotion recognition (ρ = −0.16, p = .044) and memory (ρ = −0.16, p = .048) tasks.

Regarding the reviewer’s specific concern that multiple dimensions were adapted in the memory task, we have added to the task description to clarify that only two dimensions were independently adapted, word count and study time (delay time was adjusted linearly with study time to maintain a combined time of 30 seconds). Although both word count and study time were adjusted during the adaptive procedure, all participants reached the maximum word count. Word count therefore was not a source of between-participant variation in final achieved difficulty, and final study time, which continued to vary across participants, was used to characterize task difficulty in these analyses.

These additional analyses indicate that achieved task difficulty is related to drift rate, congruent with the reviewer’s concern that the adaptive levels attained could contribute to individual differences in drift rate estimates. However, the persistence of the age–drift rate associations after accounting for achieved task difficulty suggests that variation in final task difficulty alone does not account for these associations. We have added these analyses to the Methods and Results, included the distributions of final difficulty levels in Appendix Figure A9 and revised the Discussion accordingly. The following changes have been made to the manuscript:

- Page 11 lines 306-307: ‘Delay time was adjusted according to the increase or decrease in study time to keep the combined time at 30 seconds.’

- Page 14 lines 410-420: ‘As a further check, to assess the potential influence of the adaptive difficulty procedures of the emotion recognition and the memory tasks on drift rate, we examined the distributions of achieved task difficulty and its associations with age and drift rate for both tasks. These are reported as effect sizes with 95% confidence intervals. For the memory task, although both word count and study time were adjusted by the adaptive procedure, all participants reached the maximum word count. Therefore, individual differences in final achieved difficulty were characterized using final study time, with shorter study times indicating greater difficulty. In a sensitivity analysis, we performed partial Spearman correlations between age and drift rate while controlling for achieved task difficulty. These associations were adjusted for multiple comparisons using the Benjamini–Hochberg FDR procedure correcting for the same number of associations as in the main analyses).’

- Pages 19-20 lines 536-547: ‘Given the adaptive nature of the emotion recognition and memory tasks, we additionally examined whether the task difficulty achieved by participants was associated with age and drift rate. In both tasks, lower values of the adaptive parameter represented greater task difficulty. In the emotion recognition task, age was positively associated with the final adaptive level (ρ = 0.21, 95% CI [0.08,0.35]) whereas drift rate was negatively associated with the final adaptive level (ρ = -0.15, 95% CI [-0.29, -0.01]). In the memory task, the association between age and final adaptive level was ρ = 0.12 (95% CI [−0.03, 0.27]), while drift rate was negatively associated with the final adaptive level (ρ = −0.24, 95% CI [−0.38, −0.10]). In sensitivity analyses, partial Spearman correlations controlling for achieved task difficulty showed that the negative association between age and drift rate remained significant for both emotion recognition (ρ = -0.16, p = 0.044) and memory (ρ = -0.16, p = 0.048) tasks. The distributions of achieved task difficulty are displayed in Appendix Figure A9.’

- Pages 24-25 lines 672-681: ‘The additional analyses showed that participants with higher drift rates tended to reach more difficult task conditions in both tasks, confirming that achieved difficulty is associated with individual differences in drift rate estimates. Older age was also associated with reaching less difficult conditions in the emotion recognition task, although the confidence interval for the association between age and difficulty included zero in the memory task. Importantly, however, the negative associations between age and drift rate remained significant after accounting for achieved task difficulty in both tasks, suggesting that differences in achieved difficulty alone did not account for the observed age–drift rate associations. Nevertheless, because participants were exposed to different task conditions, direct comparison of drift rate estimates across individuals should be made with caution.’

3. The temporal mismatch has not been adequately addressed by the reported sensitivity analysis

It is useful that the difference in days between DTI acquisition and online task performance was included as a covariate in the mediation analysis. However, the additional sensitivity analysis does not directly test the effect of temporal mismatch.

Restricting the sample to participants who remained cognitively and neurologically healthy at a subsequent clinical evaluation addresses the possibility of overt intervening neurological or cognitive disorder. It does not establish that white matter integrity or processing speed remained stable during the three- to four-year interval between measurements. Normal age-related changes can occur even in individuals who remain clinically healthy.

Moreover, the manuscript’s statement that the pattern of associations was “generally consistent with the primary analyses” is not well supported by Appendix Table A3. For the association between the RT-based speed composite and drift rate, the estimates changed as follows:

• delay discounting: approximately (\rho=-.27) to (\rho=-.17);

• emotion recognition: approximately (\rho=-.09) to (\rho=+.08);

• memory: approximately (\rho=-.27) to (\rho=-.05).

Thus, two associations were substantially attenuated and one changed direction. This cannot be described solely as a loss of statistical significance caused by wider confidence intervals. The Results and Discussion should report this attenuation accurately.

Ideally, the authors should conduct sensitivity analyses using progressively narrower measurement intervals, even if the resulting estimates are imprecise, or model the temporal difference continuously in all analyses involving non-concurrent measures. At minimum, the manuscript must clearly distinguish between sensitivity to subsequent clinical status and sensitivity to measurement timing, as these are not equivalent.

The mediation sensitivity result reported for the reduced sample, (p=.006), should also be identified explicitly as either corrected or uncorrected. Its inferential status is currently unclear.

We thank the reviewer for this clarification and agree that our previous sensitivity analysis addressed the potential influence of intervening clinical neurological or cognitive conditions rather than the temporal mismatch between measurements itself. We have revised the manuscript to distinguish these two issues explicitly. We have also revised our description of the results from this analysis. In particular, associations between the RT-based speed composite and drift rate were attenuated in the restricted sample, with the emotion-recognition association also changing direction. We therefore no longer describe these results as generally consistent with the primary analyses or attribute the differences solely to reduced statistical power. We have also clarified the inferential status of the mediation result in the reduced sample: the reported p = 0.003 was FDR-corrected (note the change from 0.006 to 0.003 due to the correction for 4 associations instead of 10).

As suggested by the reviewer, to address measurement timing more directly, successive sensitivity analyses were performed utilizing progressively narrower time windows for linkage to in-person neuropsychological and neuroimaging testing, which are now displayed in Appendix Figure A10 and A11.

The following changes were made to the manuscript:

- Pages 15 lines 431-436: ‘To assess the potential influence of intervening changes in clinical status during the time gap between the online task evaluation and the RT-based speed composite and neuroimaging acquisitions, sensitivity analyses for hypothesis ii and exploratory hypothesis iii were performed including only participants for whom a subsequent longitudinal evaluation following completion of the online tasks was available and confirmed that they remained cognitively and neurologically healthy.’

- Page 15 lines 439-445: ‘To directly assess the potential influence of temporal mismatch between measurements, additional sensitivity analyses were performed utilizing progressively narrower time windows for linkage to in-person neuropsychological and neuroimaging testing. For analyses involving the RT-based speed composite, these time windows were ≤1 year, ≤2 years, and (as originally preregistered) ≤3 years; for analyses involving neuroimaging measures, these time windows were ≤1 year, ≤ 2 years, ≤3 years, and (as originally preregistered) ≤4 years. These are presented graphically with Confidence Intervals.’

- Page 20 lines 565-569: ‘In this sensitivity analysis, associations between the RT-based speed composite and drift rate were attenuated relative to the primary analyses for the delay discounting and memory tasks, while the association for the emotion recognition task was weak and changed direction. Confidence intervals were also wider, reflecting the substantially reduced sample sizes, and none of the associations were statistically significant.’

- Page 20 lines 569-574: ‘ Appendix Figure A10 demonstrates the sensitivity analysis focusing on temporal mismatch between task performance and in-person neuropsychological testing for the elements included in the RT-based speed composite. Because of restrictions on in-person evaluation during the COVID-19 pandemic, no participants had in-person evaluations within 1 year of the emotion recognition or memory tasks. and few had in-person evaluations within 2 years of these tasks.’

- Page 21 line 594: ‘…p=0.003, after correcting for multiple comparisons.’

- Page 21 lines 594-597: ‘Appendix Figure A11 demonstrates the sensitivity analysis focusing on temporal mismatch between task performance and in-person neuroimaging; the direction and magnitude of indirect effects were not substantially changed by the use of narrower time windows.’

- Page 25 lines 705-707: ‘However, associations between the RT-based speed composite and drift rate were attenuated or changed direction in the restricted sensitivity analysis, warranting caution in interpreting these findings, particularly given the temporal separation between assessments.’

4. The mediation result remains overinterpreted and is described inconsistently

The authors report an uncorrected indirect-effect (p)-value of .036, which becomes .067 after multiple-comparison correction. Accordingly, this result does not meet the inferential criterion adopted in the revised manuscript.

Despite this, the manuscript continues to use statements such as:

• “Global FA showed a significant indirect effect”;

• “white matter microstructure changes have a significant indirect effect”;

• “this association is partially accounted for by changes in white matter microstructure”; and

• “global FA might partially account for the association.”

These formulations are not fully consistent with the corrected result. Some also continue to imply a mechanistic or causal chain, despite the cross-sectional design and temporal separation of the measures. The response letter states that causal language has been changed throughout the manuscript, but several of the examples presented as revised wording still retain mechanistic implications.

The Abstract formulation—“we show that this association … might be partially accounted for by global fractional anisotropy”—is also too strong. The phrase “we show” is difficult to reconcile with an exploratory effect that did not survive correction.

A more accurate formulation would be:

“An exploratory indirect association involving global FA was observed in the emotion-recognition task, but it did not remain statistically significant after correction for multiple comparisons.”

Similar wording should be used consistently in the Results and Discussion. The authors should avoid describing the effect as significant without immediately specifying that this refers only to the uncorrected result. They should also avoid “account for,” “mediate the effects of age,” or equivalent mechanistic language.

The result may be reported as hypothesis-generating, but it should not support substantive conclusions about white matter explaining age-related changes in drift rate.

We thank the reviewer for this comment and agree that our previous wording remained too strong given that the indirect effect did not remain statistically significant after correction for multiple comparisons. We have therefore revised the manuscript throughout to clearly distinguish the uncorrected exploratory finding from the corrected result and have removed language implying that global FA accounts for or mediates the association between age and drift rate. Specifically, the indirect association involving global FA in the emotion recognition task is now reported as an exploratory finding that was observed at the uncorrected level (p = 0.036) but did not remain statistically significant following FDR correction (p = 0.134). We have also revised the Abstract, Results, Discussion, and Conclusion to avoid mechanistic or causal interpretations and to describe this finding as hypothesis-generating.

The following changes have been made to the manuscript:

- Page 1 lines 24-26: ‘…in an exploratory analysis, the association for the emotion recognition task was mediated by global fractional anisotropy but this did not survive multiple-comparison correction.’

- Page 21 lines 585-589: ‘An exploratory indirect association involving global FA was observed between age and drift rate in the emotion recognition task. Unstandardized indirect effects were computed for each of 10000 bootstrapped samples. The bootstrapped unstandardized indirect effect was -1.69e-3 (uncorrected p = 0.036) but did not remain statistically significant after correcting for multiple comparisons (p = 0.134).’

- Pages 23 lines 622-625: ‘…our exploratory analysis for the association between age and drift rate in the emotion recognition task produced evidence for mediation via global FA, although this did not remain statistically significant after correction for multiple comparisons.’

- Page 26 lines 713-714: ‘…identified an exploratory indirect association involving global FA in the association between age and drift rate in the emotion recognition task.’

- Page 26 line 715: ‘This finding is consistent with…’

- Page 26 lines 717-718: ‘…global FA may be particularly relevant for investigating the relationship between age and drift rate.’

- Page 26 lines 718: ‘However, given that the finding we report, …’

5. The conclusions concerning differences between tasks should be further softened

The authors appropriately acknowledge that a significant association in two tasks and a nonsignificant association in a third task do not demonstrate a statistically significant difference between tasks. This is a welcome improvement.

Nevertheless, expressions such as “may be more evident in some cognitive domains than others” and “may be more apparent” still invite a between-task interpretation that was not formally tested. Given that the delay-discounting association was in the same direction as the other associations, and given the differences in task architecture, response coding, trial numbers, and adaptive procedures, the safest conclusion is that evidence for an association was obtained in two tasks but not in the third. The data do not establish that the population-level associations differ across tasks.

The manuscript should distinguish clearly between:

• evidence for an association within each task; and

• evidence that the magnitude of the association differs between tasks.

Only the former was tested.

We thank the reviewer for this comment. We have now removed statements suggesting that the age–drift rate association may be “more evident” or “more apparent” in some cognitive domains than others. Throughout the manuscript, we now distinguish between evidence for an association within individual tasks and evidence for differences in the magnitude of associations across tasks. Specifically, we report that evidence for an association between age and drift rate was observed in the emotion recognition and memory tasks but not in the delay discounting task, while explicitly noting that between-task differences in the magnitude of these associations were not formally tested.

- Page 1 lines 26-27: ‘Our results suggest age-related differences in information processing within cognitive contexts…’

- Page 23 lines 630-632: ‘…(reflecting slower information accumulation within a given task context) with evidence for this association observed in two of the three cognitive domains examined.’

- Page 29 lines 816-817: ‘…reflected in task-specific patterns of evidence accumulation across cognitive domains…’

6. The delay-discounting model requires further clarification

The authors appropriately acknowledge that the delay-discounting DDM is conditional on the first-stage estimate of (k), and they now discuss joint hierarchical modelling as a preferable future approach. This improves the manuscript.

However, two additional issues should be clarified.

First, the Methods indicate that (k) was estimated with bounds of (-10^{10}) and (10^{10}). A standard hyperbolic temporal-discounting parameter is ordinarily constrained to a theoretically meaningful non-negative range. Allowing very large negative values may create theoretically invalid discount functions and potential singularities in (V=A/(1+kD)). The authors should justify these bounds, report whether any negative estimates occurred, and preferably repeat the estimation using theoretically appropriate constraints.

Second, because (k) is estimated from the same choices that are subsequently coded as consistent or inconsistent, the resulting DDM parameter should be interpreted cautiously. It is not equivalent to a drift rate estimated from independently defined correct and incorrect responses. This difference needs to be reflected not only in the limitations but also in the substantive interpretation of the delay-discounting results.

We thank the reviewer for raising this point. We agree that the hyperbolic discounting parameter is theoretically non-negative and that the broad bounds used in our original numerical optimization were unnecessarily permissive. We therefore inspected the estimated k values. Of the negative estimates, all but two were extremely small (roughly 10⁻¹⁵) and can be considered numerical approximations to zero. Two participants had negative estimates of k = −0.019. We have clarified the original optimization procedure and the occurrence of negative estimates in the Methods.

We also agree that the delay discounting task drift rate differs conceptually from that obtained from tasks with independently defined correct and incorrect responses. We have further clarified throughout the manuscript that responses in this task were classified according to consistency with each participant’s individually estimated discounting function, which was itself estimated from the same choice data. Accordingly, delay discounting drift rate is interpreted as evidence accumulation toward choices consistent with the individually estimated preference model, rather than evidence accumulation toward an independently defined correct response.

The following changes were made to the manuscript:

- Page 12 lines 346-350: ‘…to avoid imposing constraints on the numerical optimization. Although this allowed negative k estimates, inspection of the estimated values showed that 25 negative estimates were extremely small (on the order of 10-15-10-16) and were considered numerical approximations to zero, while two participants had negative estimates (k = −0.019).’

- Page 12 lines 355-357: ‘However, because this tendency was estimated from the same choices subsequently classified as consistent or inconsistent based on the estimated k, the resulting drift rate could differ from that obtained when response accuracy is defined independently of participants’ choices.’

- Page 24 lines 658-661: ‘Moreover, because k was estimated from the same choices that were subsequently classified as consistent or inconsistent, the resulting drift rate may not be equivalent to one estimated from independently defined correct and incorrect responses.’

7. The parameter-recovery analysis is useful but should not be overstated

The addition of a parameter-recovery analysis is welcome. Nevertheless, high correlations between empirical parameter estimates and estimates recovered from data simulated from those same fitted parameters do not provide a complete assessment of parameter recovery.

Correlations can be high when there is substantial between-participant variation even if estimates are systematically biased or imprecise. A more informative evaluation would also report measures such as bias, root mean squared error, regression slope and intercept, or interval coverage, ideally across repeated simulations.

If the authors retain the current analysis, it should be described more narrowly as evidence that the rank ordering of participants was reasonably recoverable under the fitted model and observed trial counts, rather than as comprehensive evidence of “good recovery.” The inability to assess test–retest reliability is understandable and should remain acknowledged.

We thank the reviewer for this clarification. We have now revised the Methods, Results, and figure caption to describe the analysis more narrowly and no longer characterize the observed correlations as demonstrating “good parameter recovery.” Instead, we interpret them as showing that the rank ordering of participants was well recovered under the fitted model and observed trial counts.

The following changes were made to the manuscript:

- Page 14 lines 392-393: ‘…, to assess preservation of the rank ordering of parameter values.’

- Page 17 lines 500-501: ‘…the rank ordering of the estimated DDM parameters was well preserved across all three tasks, …’

8. Multiple-comparison correction is improved but requires clearer reporting

The introduction of FDR correction represents an important improvement. However, the correction procedure and the status of the reported p-values should be described more consistently.

The Methods should state clearly which families of tests were subjected to FDR correction and whether the p-values subsequently reported in the text, tables, and figures are adjusted unless explicitly identified otherwise. Standalone tables and figure captions should also provide sufficient information to be interpreted independently. For example, the Figure 1 caption should refer to an FDR-adjusted significance threshold rather than simply stating that associations were significant “at a p-value of 0.05.” Unadjusted p-values may be reported for transparency, but all inferential conclusions should be based on the adjusted values.

The following points also require clarification:

• whether the p-values reported for the sensitivity analyses are adjusted or unadjusted;

• whether the mediation sensitivity result of (p=.006) is adjusted or unadjusted;

• the exact tests included in each FDR family; and

• whether the ancillary mediation analyses involving boundary separation and non-decision time were included in any multiplicity correction

In addition, the rationale for combining six preregistered behavioural associations and four exploratory mediation tests into a single ten-test FDR family is unclear. These tests address conceptually distinct hypotheses, and the mediation analyses appear to have been conducted conditionally, following evidence of associations in the preceding analyses. Combining them into one family may therefore allow the rank ordering of strong behavioural p-values to influence the adjustment of conceptually separate exploratory mediation results. The authors should either provide a clear statistical and conceptual justification for this combined family or apply separately defined correction families to the behavioural and mediation analyses. If the correction procedure is revised, all affected adjusted p-values and corresponding conclusions should be updated accordingly.

We thank the reviewer for this comment. We agree that combining the preregistered behavioral associations and exploratory mediation analyses into a single FDR family was not sufficiently justified given that these analyses address conceptually distinct hypotheses. We have therefore revised the multiple comparison procedure and applied Benjamini–Hochberg FDR correction separately to the four exploratory mediation analyses involving drift rate. All affected adjusted p-values and corresponding conclusions have been updated accordingly. We have also revised the Methods, tables and figure captions to explicitly identify the tests included in each correction family and whether reported p-values are adjusted or unadjusted.

Finally, given that we now report associations between boundary separation and non-decision time with age descriptively as effect sizes and 95% Confidence Intervals and do not associate them with p-values or significance-based inference, we have removed analyses involving these parameters from the mediation analyses.

The following changes were made to the manuscript:

- Page 14 lines 404-406: ‘These ancillary analyses were not included in the FDR correction, as they were treated as ancillary and are reported descriptively as effect sizes with 95% confidence intervals, without p-values or significance-based inference.’

- Page 15 lines 436-437: ‘All sensitivity analyses followed the same FDR adjustments for multiple comparisons as the main analysis.’

- Page 15 lines 437-438: ‘All p-values are reported after adjustment for multiple comparisons unless explicitly stated otherwise.’

- Page 19 lines 530-531 (Figure 1 legend): ‘…at an FDR-adjusted p-value of 0.05 using the Benjamini–Hochberg procedure…’

- Page 19 lines 534-535 (Table 3 legend): ‘P-values for drift rate are Benjamini–Hochberg FDR-adjusted.’

- Page 21 lines 578-579 (Table 4 legend): ‘P-values for drift rate are Benjamini–Hochberg FDR-adjusted.’

9. Issues that have been addressed adequately

Several previous concerns have been addressed sufficiently and, in my view, need not prevent publication after the remaining issues are resolved.

The authors have:

• substantially expanded the task descriptions;

• clarified the definition and clinical evaluation of neurologically healthy participants;

• explained the purpose of normalization to the young-adult reference sample;

• replaced longitudinal terminology with cross-sectional terminology;

• acknowledged the uneven age distribution and limited oldest-old sample;

• added descriptive presentation by age group;

• reported diffusion-specific quality-control procedures and motion statistics;

• provided reaction-time distributions before and after trimming;

• repeated the principal analyses without trimming the slowest 5% of trials; and

• justified the preregistered use of Spearman correlations as tests of monotonic associations.

I consider the explanation for not fitting nonlinear age trajectories broadly acceptable. A nonlinear exploratory analysis could still be informative, but it is not essential to the preregistered monotonic association hypothesis, provided that the manuscript no longer makes claims about developmental or aging trajectories.

Overall assessment

The manuscript has improved, and I recognize that the authors have made a serious effort to respond to a large number of comments. The study has meaningful strengths, including preregistration, a relatively large older-adult sample, explicit model-fit diagnostics, sensitivity analyses, and the integration of behavioural modelling with neuroimaging measures.

However, several central concerns have not yet received satisfactory answers. In particular, the manuscript still moves too readily from task-specific DDM parameters to a general cognitive processing-speed interpretation; the consequences of adaptive task difficulty are not adequately analysed or acknowledged; the temporal sensitivity analysis does not test temporal proximity and is described more positively than its results warrant; and the exploratory, non-significant-after-correction mediation effect continues to be presented using significant and partly mechanistic language.

Therefore, I would support a further substantial revision focused on aligning the claims with what the analyses can establish. The study may ultimately support a useful and appropriately nuanced account of age-related associations with task-specific DDM parameters. It does not currently provide convincing evidence for a unitary, task-general slowing of evidence accumulation or for white matter microstructure as a mechanism explaining that slowing.

Attachment Submitted filename: Response_to_Reviewers_auresp_2.docx

# Peer review: Decision Letter 2

**DOI:** 10.1371/journal.pone.0361899.r005

**Authors:** Pramod Yadav

5 Oct 2026

Age-related processes impacting cognitive processing speed across an older adult cohort: a drift diffusion model analysis

PLOS One

Dear Dr.  Sanches,

Thank you for submitting your manuscript to PLOS One. After careful consideration, we feel that it has merit but does not fully meet PLOS One’s publication criteria as it currently stands. Therefore, we invite you to submit a revised version (Minor) of the manuscript that addresses the points raised during the review process.

Please submit your revised manuscript by Nov 19 2026 11:59PM. If you will need more time than this to complete your revisions, please reply to this message or contact the journal office at plosone@plos.org. When you're ready to submit your revision, log on to https://www.editorialmanager.com/pone/ and select the 'Submissions Needing Revision' folder to locate your manuscript file.

Please include the following items when submitting your revised manuscript:

- A letter that responds to each point raised by the academic editor and reviewer(s). You should upload this letter as a separate file labeled 'Response to Reviewers'.
- A marked-up copy of your manuscript that highlights changes made to the original version. You should upload this as a separate file labeled 'Revised Manuscript with Track Changes'.
- An unmarked version of your revised paper without tracked changes. You should upload this as a separate file labeled 'Manuscript'.
- If you would like to make changes to your financial disclosure, please include your updated statement in your cover letter. Guidelines for resubmitting your figure files are available below the reviewer comments at the end of this letter.

If applicable, we recommend that you deposit your laboratory protocols in protocols.io to enhance the reproducibility of your results. Protocols.io assigns your protocol its own identifier (DOI) so that it can be cited independently in the future. For instructions see: https://journals.plos.org/plosone/s/submission-guidelines#loc-laboratory-protocols. Additionally, PLOS One offers an option for publishing peer-reviewed Lab Protocol articles, which describe protocols hosted on protocols.io. Read more information on sharing protocols at https://plos.org/protocols?utm_medium=editorial-email&utm_source=authorletters&utm_campaign=protocols.

As the corresponding author, your ORCID iD is verified in the submission system and will appear in the published article. PLOS supports the use of ORCID, and we encourage all coauthors to register for an ORCID iD and use it as well. Please encourage your coauthors to verify their ORCID iD within the submission system before final acceptance, as unverified ORCID iDs will not appear in the published article. *Only*  the individual author can complete the verification step; PLOS staff *cannot*  verify ORCID iDs on behalf of authors.

We look forward to receiving your revised manuscript.

Kind regards,

Pramod K. Yadav, Ph. D.

Academic Editor

PLOS One

Journal Requirements:

If the reviewer comments include a recommendation to cite specific previously published works, please review and evaluate these publications to determine whether they are relevant and should be cited. There is no requirement to cite these works unless the editor has indicated otherwise.

Please review your reference list to ensure that it is complete and correct. If you have cited papers that have been retracted, please include the rationale for doing so in the manuscript text, or remove these references and replace them with relevant current references. Any changes to the reference list should be mentioned in the rebuttal letter that accompanies your revised manuscript. If you need to cite a retracted article, indicate the article’s retracted status in the References list and also include a citation and full reference for the retraction notice.

[Note: HTML markup is below. Please do not edit.]

Reviewers' comments:

Reviewer’s Responses to Questions

**Comments to the Author**

**Reviewer 2**

Reviewer #2: (No Response)

**********

2. Is the manuscript technically sound, and do the data support the conclusions?

Reviewer #2: Yes

**********

3. Has the statistical analysis been performed appropriately and rigorously?

Reviewer #2: Yes

**********

4. Have the authors made all data underlying the findings in their manuscript fully available?

The PLOS Data policy

Reviewer #2: (No Response)

**********

5. Is the manuscript presented in an intelligible fashion and written in standard English?

Reviewer #2: Yes

**********

Reviewer #2: I appreciate the considerable effort the authors have invested in this revision. The additional analyses and the more careful interpretation have addressed the great majority of my previous concerns, and the manuscript is now substantially clearer, more transparent, and appropriately nuanced. In particular, the task-specific interpretation of drift rate, the analyses of adaptive task difficulty and temporal mismatch, and the revised multiple-comparison procedure represent important improvements. I have only two remaining points, both of which should be relatively straightforward to address.

1. Interpretation of the mediation analysis

The indirect association involving global FA was no longer statistically significant after correction for multiple comparisons: the uncorrected p-value was .036, whereas the FDR-adjusted p-value was .134. Nevertheless, the Abstract still states that the association “was mediated by global fractional anisotropy,” and the Discussion refers to “evidence for mediation.” These formulations remain stronger than warranted by an exploratory result that did not remain significant after correction, particularly given the cross-sectional design.

The white-matter finding need not be omitted entirely, as it remains an interesting, hypothesis-generating observation. However, it should be described consistently as an exploratory indirect association that was not statistically significant after correction. The Conclusion may mention it in this explicitly qualified form rather than stating more generally that age-related slowing “might be linked to age-related loss of white matter integrity.” The term “white matter integrity” should also be avoided or qualified, given the acknowledged limitations of interpreting FA as a direct measure of integrity.

2. Negative discounting-parameter estimates

Inspection of the estimated discounting parameters revealed two genuinely negative values (\(k=-.019\)), in addition to very small negative values that can reasonably be regarded as numerical approximations to zero. Because the hyperbolic discounting parameter is theoretically expected to be zero or positive, merely reporting the negative estimates does not fully resolve the issue.

The authors should preferably repeat the estimation while restricting \(k\) to non-negative values and confirm whether the classification of responses, the delay-discounting DDM estimates, and the substantive conclusions remain unchanged. At minimum, they should provide a sensitivity analysis excluding the two participants with clearly negative estimates.

Subject to these two targeted revisions, I believe the manuscript would be suitable for publication.

**********

what does this mean?). If published, this will include your full peer review and any attached files.

If you choose “no”, your identity will remain anonymous but your review may still be made public.

**Do you want your identity to be public for this peer review?**  For information about this choice, including consent withdrawal, please see our Privacy Policy

Reviewer #2: No

**********

[NOTE: If reviewer comments were submitted as an attachment file, they will be attached to this email and accessible via the submission site. Please log into your account, locate the manuscript record, and check for the action link "View Attachments". If this link does not appear, there are no attachment files.]

To ensure your figures meet our technical requirements, please review our figure guidelines: https://journals.plos.org/plosone/s/figures

You may also use PLOS’s free figure tool, NAAS, to help you prepare publication quality figures: https://journals.plos.org/plosone/s/figures#loc-tools-for-figure-preparation.

NAAS will assess whether your figures meet our technical requirements by comparing each figure against our figure specifications.

# Author comment: Author response to Decision Letter 3

**DOI:** 10.1371/journal.pone.0361899.r006

12 Oct 2026

Reviewer #2

I appreciate the considerable effort the authors have invested in this revision. The additional analyses and the more careful interpretation have addressed the great majority of my previous concerns, and the manuscript is now substantially clearer, more transparent, and appropriately nuanced. In particular, the task-specific interpretation of drift rate, the analyses of adaptive task difficulty and temporal mismatch, and the revised multiple-comparison procedure represent important improvements. I have only two remaining points, both of which should be relatively straightforward to address.

1. Interpretation of the mediation analysis

The indirect association involving global FA was no longer statistically significant after correction for multiple comparisons: the uncorrected p-value was .036, whereas the FDR-adjusted p-value was .134. Nevertheless, the Abstract still states that the association “was mediated by global fractional anisotropy,” and the Discussion refers to “evidence for mediation.” These formulations remain stronger than warranted by an exploratory result that did not remain significant after correction, particularly given the cross-sectional design.

The white-matter finding need not be omitted entirely, as it remains an interesting, hypothesis-generating observation. However, it should be described consistently as an exploratory indirect association that was not statistically significant after correction. The Conclusion may mention it in this explicitly qualified form rather than stating more generally that age-related slowing “might be linked to age-related loss of white matter integrity.” The term “white matter integrity” should also be avoided or qualified, given the acknowledged limitations of interpreting FA as a direct measure of integrity.

We thank the reviewer for this comment and agree that the previous wording remained too strong given that the exploratory indirect association involving global FA did not remain statistically significant after multiple-comparison correction. We have revised the Abstract, Discussion, and Conclusion to consistently describe this finding as an exploratory indirect association that did not remain statistically significant after correction, rather than as evidence of mediation. The finding is now presented explicitly as hypothesis-generating and as requiring further investigation.

The following changes were made to the manuscript:

- Page 1 lines 24-26: ‘in an exploratory analysis, an indirect association involving global fractional anisotropy was observed for the emotion recognition task, although this did not survive multiple-comparison correction.’

- Page 23 line 631: ‘…identified an indirect association involving global FA, although this did not remain statistically significant after correction for multiple comparisons.’

- Page 29 lines 823-826: ‘An exploratory indirect association involving global FA, although not statistically significant after multiple-comparison correction, suggests that the potential role of age-related differences in white matter microstructure warrants further investigation.’

2. Negative discounting-parameter estimates

Inspection of the estimated discounting parameters revealed two genuinely negative values (\(k=-.019\)), in addition to very small negative values that can reasonably be regarded as numerical approximations to zero. Because the hyperbolic discounting parameter is theoretically expected to be zero or positive, merely reporting the negative estimates does not fully resolve the issue.

The authors should preferably repeat the estimation while restricting \(k\) to non-negative values and confirm whether the classification of responses, the delay-discounting DDM estimates, and the substantive conclusions remain unchanged. At minimum, they should provide a sensitivity analysis excluding the two participants with clearly negative estimates.

Subject to these two targeted revisions, I believe the manuscript would be suitable for publication.

We thank the reviewer for this suggestion. We performed the requested sensitivity analysis excluding the two participants with clearly negative estimates of the discounting parameter (k = −0.019). The results were essentially unchanged. The Spearman correlation between age and drift rate was ρ = −0.097 (p = 0.27) after excluding these participants, compared with ρ = −0.098 (p = 0.25) in the full sample. Similarly, the correlation between the RT-based speed composite and drift rate was ρ = −0.27 (p = 0.005) after exclusion, similar to that observed in the full sample (ρ = −0.274, p = 0.003). Thus, exclusion of the two participants with clearly negative k estimates did not affect the substantive conclusions for the delay-discounting task.

We have added this sensitivity analysis to the manuscript:

- Page 14 lines 407-409: ‘Moreover, for the delay-discounting task, a sensitivity analysis for the associations of drift rate with age and the RT-based speed composite was performed after excluding the two participants with k = −0.019.’

- Page 18 lines 530-531: ‘Excluding the two participants with clearly negative k estimates did not noticeably alter the delay-discounting task results (ρ = −0.097, p = 0.27).’

- Page 20 lines 567-569: ‘Excluding the two participants with clearly negative k estimates did not noticeably alter the delay-discounting task results (ρ = −0.27, p = 0.005).’

Attachment Submitted filename: Response_to_Reviewers_auresp_3.docx

# Peer review: Decision Letter 3

**DOI:** 10.1371/journal.pone.0361899.r007

**Authors:** Pramod Yadav

19 Oct 2026

Age-related processes impacting cognitive processing speed across an older adult cohort: a drift diffusion model analysis

PONE-D-26-18379R3

Dear Dr. Maria Clara Sanches,

We’re pleased to inform you that your manuscript has been judged scientifically suitable for publication and will be formally accepted for publication once it meets all outstanding technical requirements.

Within one week, you’ll receive an e-mail detailing the required amendments. When these have been addressed, you’ll receive a formal acceptance letter and your manuscript will be scheduled for publication.

An invoice will be generated when your article is formally accepted. Please note, if your institution has a publishing partnership with PLOS and your article meets the relevant criteria, all or part of your publication costs will be covered. Please make sure your user information is up-to-date by logging into Editorial Manager at Editorial Manager® and clicking the ‘Update My Information' link at the top of the page. For questions related to billing, please contact billing support.

If your institution or institutions have a press office, please notify them about your upcoming paper to help maximize its impact. If they’ll be preparing press materials, please inform our press team as soon as possible -- no later than 48 hours after receiving the formal acceptance. Your manuscript will remain under strict press embargo until 2 pm Eastern Time on the date of publication. For more information, please contact onepress@plos.org.

Kind regards,

Pramod K. Yadav, Ph. D.

Academic Editor

PLOS One

Additional Editor Comments (optional):

Reviewers' comments:

Reviewer’s Responses to Questions

**Comments to the Author**

Reviewer #2: All comments have been addressed

**********

2. Is the manuscript technically sound, and do the data support the conclusions?

Reviewer #2: Yes

**********

3. Has the statistical analysis been performed appropriately and rigorously?

Reviewer #2: Yes

**********

4. Have the authors made all data underlying the findings in their manuscript fully available?

The PLOS Data policy

Reviewer #2: (No Response)

**********

5. Is the manuscript presented in an intelligible fashion and written in standard English?

Reviewer #2: Yes

**********

Reviewer #2: (No Response)

**********

what does this mean?). If published, this will include your full peer review and any attached files.

If you choose “no”, your identity will remain anonymous but your review may still be made public.

**Do you want your identity to be public for this peer review?**  For information about this choice, including consent withdrawal, please see our Privacy Policy

Reviewer #2: No

**********

# Editor report: Acceptance letter

**DOI:** 10.1371/journal.pone.0361899.r008

**Authors:** Pramod Yadav

PONE-D-26-18379R3

PLOS One

Dear Dr. Sanches,

I'm pleased to inform you that your manuscript has been deemed suitable for publication in PLOS One. Congratulations! Your manuscript is now being handed over to our production team.

At this stage, our production department will prepare your paper for publication. This includes ensuring the following:

* All references, tables, and figures are properly cited

* All relevant supporting information is included in the manuscript submission,

* There are no issues that prevent the paper from being properly typeset

You will receive further instructions from the production team, including instructions on how to review your proof when it is ready. Please keep in mind that we are working through a large volume of accepted articles, so please give us a few days to review your paper and let you know the next and final steps.

Lastly, if your institution or institutions have a press office, please let them know about your upcoming paper now to help maximize its impact. If they'll be preparing press materials, please inform our press team within the next 48 hours. Your manuscript will remain under strict press embargo until 2 pm Eastern Time on the date of publication. For more information, please contact onepress@plos.org.

You will receive an invoice from PLOS for your publication fee after your manuscript has reached the completed accept phase. If you receive an email requesting payment before acceptance or for any other service, this may be a phishing scheme. Learn how to identify phishing emails and protect your accounts at https://explore.plos.org/phishing.

If we can help with anything else, please email us at customercare@plos.org.

Thank you for submitting your work to PLOS One and supporting open access.

Kind regards,

PLOS One Editorial Office Staff

on behalf of

Dr. Pramod K. Yadav

Academic Editor

PLOS One
